# Luminescent Fe(III) Complex Sensitizes Aerobic Photon Upconversion and Initiates Photocatalytic Radical Polymerization

**DOI:** 10.1021/jacs.4c14248

**Published:** 2024-12-10

**Authors:** Pengyue Jin, Xinhuan Xu, Yongli Yan, Heinrich Hammecke, Cui Wang

**Affiliations:** † Department of Biology and Chemistry, Osnabrück University, Barbarastraße 7, Osnabrück 49076, Germany; ‡ Key Laboratory of Photochemistry, Institute of Chemistry, Chinese Academy of Sciences, Beijing 100190, China

## Abstract

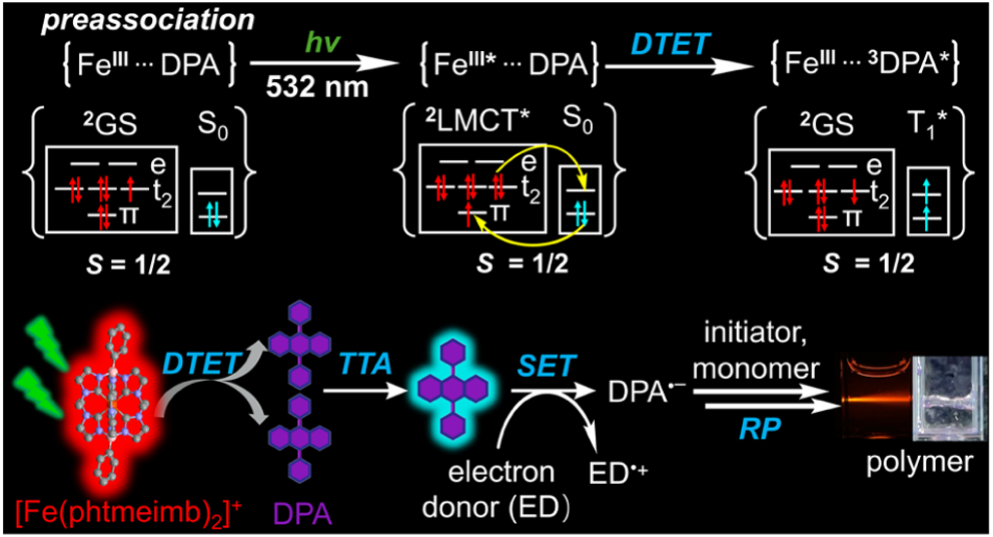

Light energy conversion often relies on photosensitizers with long-lived excited states, which are mostly made of precious metals such as ruthenium or iridium. Photoactive complexes based on highly abundant iron seem attractive for sustainable energy conversion, but this remains very challenging due to the short excited state lifetimes of the current iron complexes. This study shows that a luminescent Fe(III) complex sensitizes triplet–triplet annihilation upconversion with anthracene derivatives via underexplored doublet-triplet energy transfer, which is assisted by preassociation between the photosensitizer and the annihilator. In the presence of an organic mediator, the green-to-blue upconversion efficiency Φ_UC_ with 9,10-diphenylanthracene (DPA) as the annihilator achieves a 6-fold enhancement to ∼0.2% in aerated solution at room temperature. The singlet excited state of DPA, accessed via photon upconversion in the Fe(III)/DPA pair, allows efficient photoredox catalytic radical polymerization of acrylate monomers in a spatially controlled manner, whereas this process is kinetically hindered with the prompt DPA. Our study provides a new strategy of using low-cost iron and low-energy visible light for efficient polymer synthesis, which is a significant step for both fundamental research and future applications.

## Introduction

Sensitized triplet–triplet annihilation (TTA) upconversion (sTTA-UC) has become a popular biphotonic process used in bioimaging,^1,2^ photovoltaics,^3,4^ organic light-emitting diodes,^5^ and photocatalysis.^4,6−11^ This process converts two low-energy input photons into one higher-energy output photon under noncoherent light irradiation.^12−15^ Photoactive complexes made of platinum group metals including Pd(II), Pt(II),^16−22^ Ru(II), Ir(III),^23−28^ and Os(II)^9,29−33^ are favorable photosensitizers due to their long-lived triplet excited states, which enable efficient triplet–triplet energy transfer (TTET) to an annihilator, followed subsequently by TTA.^7,14,15^

Biphotonic sTTA-UC has become an attractive approach to drive energy-demanding photochemical reactions, because the low-energy visible irradiation light is less harmful and can avoid photodamage as well as inner-filter effects caused by high-energy UV photons.^4,6,11,34−45^ This is particularly true for photopolymerization, which is of interest, for example, for 3D printing that requires high spatial control.^11,46,47^ UC-driving photopolymerization reactions rely mostly on two-photon absorption that requires coherent laser excitation with high photon fluxes^48^ and lanthanide-doped upconversion nanoparticles.^46,47,49,50^ sTTA-UC is becoming an emerging technology for photopolymerization due to the high upconversion efficiency, the use of noncoherent low-power input light, and adjustable irradiation wavelength.^11,34,42,43,47,51−54^ However, photosensitizers used for this purpose are mostly made of heavy metals.^11,34,42,47,52,53^

Increasing efforts are recently made to develop heavy-atom-free photosensitizers for sTTA-UC.^55−60^ Photoactive first–row transition metal complexes are seen as further sustainable alternatives to those based on platinum group metals, but only a few attempts have been given so far for sTTA-UC, including Cr(0),^43^ Cr(III),^61^ Mn(I),^62^ Cu(I),^63,64^ and Zn(II).^65−68^ Even fewer efforts have been made to use these photosensitizers for polymerization reactions via sTTA-UC.^43,54^

**Figure 1:**
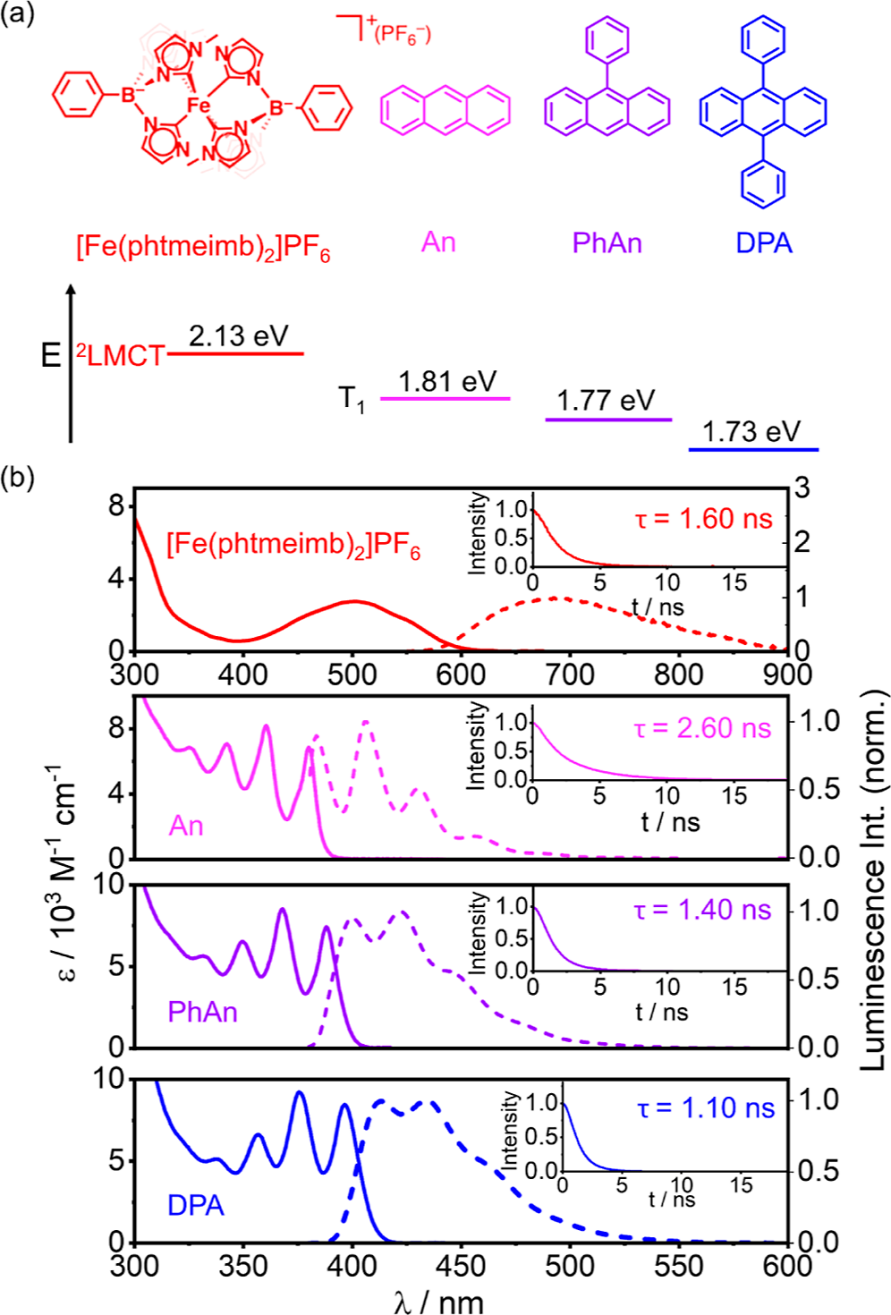
(a) Molecular structures of [Fe(phtmeimb)_2_]PF_6_ and anthracene-based annihilators, together with the ^2^LMCT excited state energy of the Fe(III) complex and the energetically lowest triplet state (T_1_) energy of the anthracenes (Supporting Information, Section 3). (b) UV–vis absorption (solid traces) and normalized luminescence spectra (dashed traces, excitation at 532 nm) of [Fe(phtmeimb)_2_]PF_6_ (40 μM) and the anthracenes in aerated DMSO at 20 °C. Inset: Normalized luminescence decay of the corresponding sample, excitation occurred with a pulsed LED at 455 nm for [Fe(phtmeimb)_2_]PF_6_ and at 390 nm for the anthracene-based annihilators (Supporting Information, Section 4).

Photosensitizers based on iron could be highly attractive for sTTA-UC due to their high natural abundance, low cost, and low-energy visible light absorption. However, this remains a significant challenge due to the extremely short excited state lifetimes for, e.g., 3d^6^ Fe(II) and 3d^5^ Fe(III) complexes, as a consequence of the excited state deactivation by the energetically low-lying metal-centered states.^69−73^ Over the past few years, remarkable progress has been achieved in elongating the lifetime of the doublet ligand-to-metal charge transfer (^2^LMCT) excited state in Fe(III) complexes.^74−78^ Among these Fe(III)-based luminophores, the [Fe(phtmeimb)_2_]PF_6_ complex with tridentate scorpionate ligands (Figure 1a) shows a long luminescence lifetime of 2.0 ns from the ^2^LMCT excited state in acetonitrile at room temperature,^75^ which enables symmetry-breaking charge separation and photoredox catalysis.^79−86^ However, examples of using Fe(III)-based photosensitizers for sTTA-UC remain scarce and underexplored.^78^ This is because (i) the doublet-triplet energy transfer (DTET) from the ^2^LMCT excited state of a Fe(III) complex to the triplet state of an organic annihilator is still poorly understood, and (ii) the up to multinanosecond lifetimes of the ^2^LMCT excited state severely limit the diffusion-controlled DTET in solution. The very recent proof-of-principle study shows that a luminescent Fe(III) carbene complex ([Fe(ImPP)_2_]^+^), which was prepared with improved synthesis to avoid a blue-emitting impurity,^87^ shows subnanosecond ^2^LMCT excited state lifetime and sensitizes red-to-green upconversion with an anthracene derivative.^78^ Further efforts seem necessary to understand the energy transfer mechanism with Fe(III)-based photosensitizers in photon upconversion and to make photoactive Fe(III) complexes suitable for energy conversion-based applications, such as photopolymerization.

In this work, we use the [Fe(phtmeimb)_2_]^+^ complex for sensitizing green-to-blue upconversion with three anthracene-based annihilators with various energy levels of the energetically lowest triplet state (T_1_), including anthracene (An), 9-phenylanthracene (PhAn), and 9,10-diphenylanthracene (DPA) (Figure 1a). The obtained luminescence quenching rate constants *k*_q_ are beyond the diffusion limit of the employed solvent due to the preassociation between [Fe(phtmeimb)_2_]^+^ and the anthracenes in their ground states. DTET was evidenced by femtosecond- and nanosecond-transient absorption spectroscopy, and the upconversion luminescence shows a delayed nature with a μs-scaled lifetime. Assisted by an organic mediator, the upconversion luminescence quantum yield Φ_UC_ for the Fe(III)/DPA pair is enhanced by a factor of 6.3 and reaches ∼0.2% in aerated DMSO at 20 °C. Driven by the Fe(III)/DPA upconversion pair, photocatalytic polymerization of acrylate monomers was efficiently initiated in a spatially controlled fashion with green irradiation light under aerobic conditions.

## Results and Discussion

DMSO is known as a deoxygenating solvent, which can be converted to sulfone by singlet oxygen that is generated by a triplet photosensitizer,^88^ and this has been used to solve the oxygen quenching issue of sTTA-UC solutions.^45,89^ Therefore, DMSO was used as the solvent throughout this study. The UV–vis absorption and luminescence spectra of [Fe(phtmeimb)_2_]^+^ in DMSO at 20 °C show an absorption band maximized at 502 nm, which is attributable to the LMCT transition with an extinction coefficient ε = 2214 M^–1^ cm^–1^ at 532 nm (excitation wavelength used in photon upconversion studies below), and a broad luminescence band centered at 670 nm from the ^2^LMCT excited state (Figure 1b), similar to those obtained in other polar solvents, such as acetonitrile, dichloromethane, and acetone.^75,81,82^ The ^2^LMCT excited state shows a photoluminescence quantum yield Φ_PL_ of 1.82% and a lifetime τ_0_ of 1.60 ns in aerated DMSO at 20 °C (Figure 1b, inset, Supporting Information, Figure S7 and S8), which corresponds to a radiative decay rate constant of *k*_r_ = Φ_PL_/τ_0_ = 1.1 × 10^7^ s^–1^, in line with the literature values obtained in dichloromethane or acetonitrile.^75,81,82^

### Doublet-Triplet energy transfer

**Figure 2:**
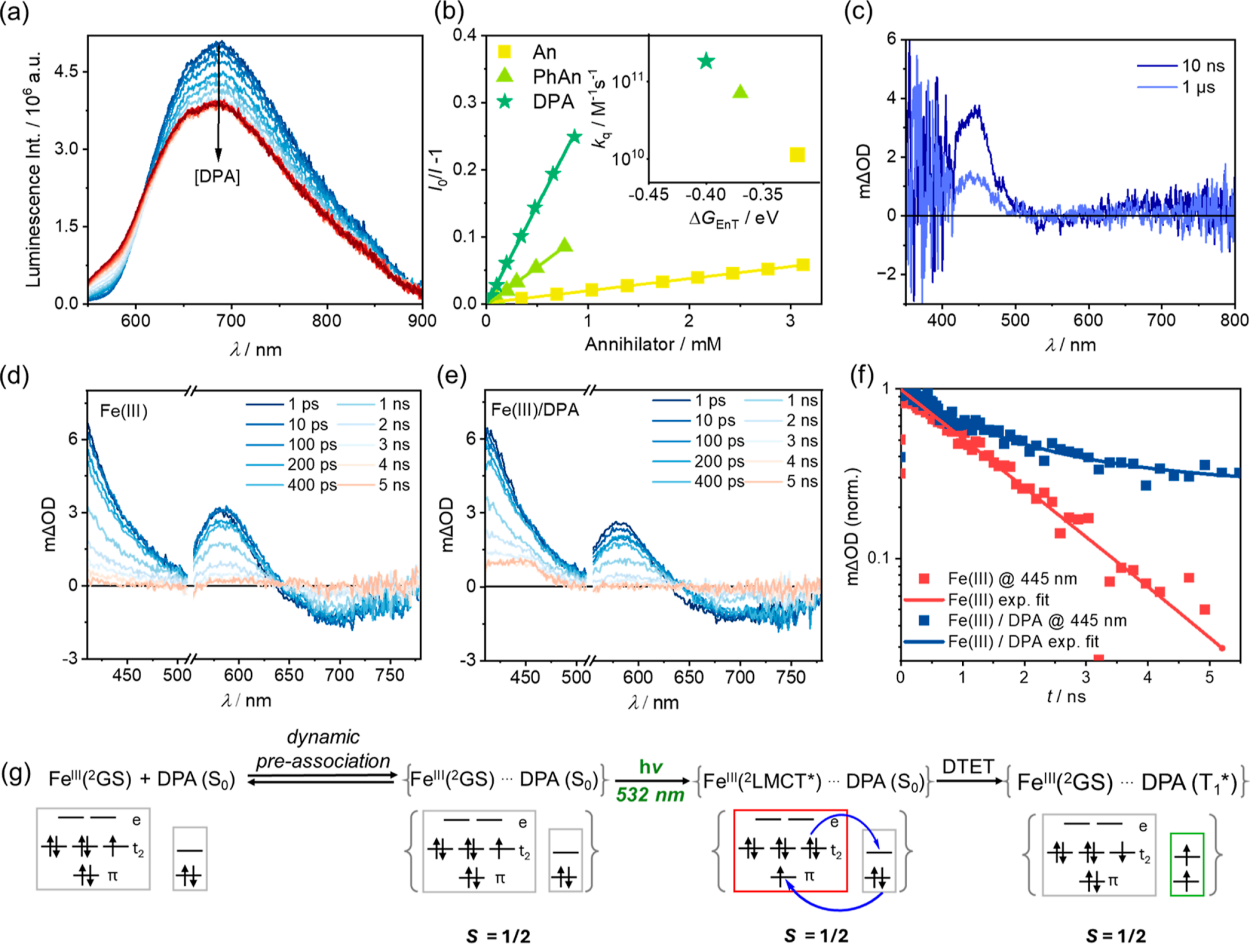
(a) Luminescence intensity-based Stern–Volmer quenching of [Fe(phtmeimb)_2_]PF_6_ (40 μM) by DPA with different concentrations (0–10 mM, blue to red) in deaerated DMSO at 20 °C. The rise of luminescence at ∼570 nm with increasing DPA concentration is attributed to the excimer fluorescence from DPA.^100−102^ (b) Stern–Volmer plots derived from the quenched luminescence intensity of [Fe(phtmeimb)_2_]PF_6_ (40 μM) by An (yellow squares), PhAn (light green triangles), and DPA (green stars) (Supporting Information, Figure S9–11); linear fitting of the data in (b) gives the quenching rate constant *k*_q_ (*k*_q_ = *K*_SV_/τ_0_, τ_0_ = 1.60 ns in aerated DMSO from the lifetime obtained in Figure 1b, inset). Inset: plot of the quenching rate constant *k*_q_ as a function of the free energy Δ*G*_EnT_ for DTET with different anthracenes. (c) UV–vis transient absorption spectra of [Fe(phtmeimb)_2_]PF_6_ (100 μM)/DPA (10 mM) in aerated DMSO at 20 °C with different time delays after excitation at 532 nm with a ns-pulsed laser (pulse energy ∼12 mJ). UV–vis transient absorption spectra of (d) [Fe(phtmeimb)_2_]PF_6_ (500 μM) and (e) [Fe(phtmeimb)_2_]PF_6_ (500 μM)/DPA (10 mM) in aerated DMSO at 20 °C with different time delays after excitation at 532 nm with a fs-pulsed laser (pulse energy ∼0.92 μJ). The wavelength region between 510 and 555 nm is removed due to the pump scattering. (f) Transient absorption decays of samples from (d) (red scatters) and (e) (blue scatters) recorded at 445 nm, together with their monoexponential (red solid traces) and biexponential (blue solid traces) fits. Excitation occurred with a fs-pulsed laser at 532 nm. (g) Reaction scheme of [Fe(phtmeimb)_2_]PF_6_ and DPA with their corresponding electronic microstates.

The ^2^LMCT excited state energy of [Fe(phtmeimb)_2_]^+^ is 2.13 eV,^75^ which is 0.32–0.40 eV above the calculated energy levels of the T_1_ state for the employed anthracenes (Figure 1a, Supporting Information, Section 3).^90^ Therefore, DTET from the ^2^LMCT state of the Fe(III) complex to the T_1_ of the employed anthracenes is thermodynamically feasible,^61,91^ and a reversed process is inhibited due to the large energy gaps. The addition of DPA quenches the luminescence intensity of [Fe(phtmeimb)_2_]^+^ in DMSO (Figure 2a), and the derived Stern–Volmer plot follows a linear fitting (Figure 2b). The quenching rate constant *k*_q_ derived from the linear regime increases in the order of Fe(III)/An < Fe(III)/PhAn < Fe(III)/DPA in DMSO, presumably due to the free energy Δ*G*_EnT_ dependence of the DTET rate constant (Figure 2b, inset, Table 1, Supporting Information, Section 5.3).^91^

**Table 1:** Photon Upconversion Parameters of the [Fe(phtmeimb)_2_]PF_6_/Anthracenes Pairs in the Presence and Absence of an Anthracene-Based Mediator^e^

	mediator	λ_em_^a^/nm	Δ*E*/eV	Φ_UC_^b^/%	τ_UC_^c^/μs	*k*_q_/M^–1^ s^–1^	Φ_DTET_^d^/%
Fe(III)/An^d^	none	407	–0.72	0.003	109	1.13 × 10^10^	12.3
Fe(III)/PhAn	none	420	–0.62	0.06	119	7.04 × 10^10^	14.5
	An			0.04	102		
Fe(III)/DPA	none	435	–0.52	0.03	110	1.82 × 10^11^	22.5 (22.9)
	An			0.19	108		
	PhAn			0.16	97		

a UC luminescence maximum.

b Upconversion luminescence quantum yield (Φ_UC_) of [Fe(phtmeimb)_2_]PF_6_ (40 μM)/annihilators (An 20 mM, PhAn 20 mM, DPA 10 mM) in the absence and presence of a given mediator (10 mM) in aerated DMSO at 20 °C. Excitation occurred with a 532 nm cw-laser at the maximal power density of 144 W cm^–2^. A 495 nm long pass filter was placed between the laser and the sample.

c Upconversion luminescence decays of the same samples used in the Φ_UC_ measurements at the UC luminescence maximum wavelength in deaerated DMSO at 20 °C.

d The DTET efficiency. The DTET efficiency is given by the equation: Φ_DTET_ = 1 – *I*/*I*_0_ = 1 – τ/τ_0_, *I*_0_ and *I* are the luminescence intensity in the absence and presence of the anthracene annihilators at the given concentrations (Supporting Information, Section 5), respectively; in parentheses is the DTET efficiency value determined from the transient absorption lifetimes of [Fe(phtmeimb)_2_]PF_6_ (500 μM) at 580 nm in the absence (τ_0_) and presence of DPA (10 mM) (τ) (Supporting Information, Section 7.1).

e Excitation occurred at 532 nm with a cw-laser.

For all the Fe(III)/anthracene pairs, the obtained *k*_q_ values are found in the region of 11.3–182 × 10^9^ s^–1^, which exceed the diffusion limit of DMSO at 20 °C (*k*_diff._ = 2.9 × 10^9^ s^–1^)^90^ by a factor of 4–63 (Table 1, Supporting Information, Section 5.1–5.3). This is likely attributed to preassociation, which occurs noncovalently between a photosensitizer and a quencher in their ground states prior to excitation.^92^ For the Fe(III)/anthracenes pairs, π–π stacking between the aromatic systems of the anthracenes and the phenyl moieties on the backbone of the Fe(III) complex seems viable. ^1^H NMR titration of the Fe(III) complex by DPA exemplarily evidences the π–π interactions, and the obtained association constants *K*_a_ follow a 1:1 model with values ranging from 149 to 295 M^–1^ for the phenyl moieties of the Fe(III) complex, in line with the *K*_a_ of 158 M^–1^ determined from the chemical shift of DPA (Supporting Information, Section 6). The magnitude of these association constants is found to be representative of weak π–π interactions occurring in a dynamic fashion.^93−96^ Ground-state preassociation is particularly beneficial for bimolecular reactions involving short excited state lifetimes because it circumvents the diffusion-controlled encounter and allows efficient bimolecular electron- and energy transfer.^77,92,97^ Assuming that the observed luminescence quenching of the Fe(III) complex is all attributed to the energy transfer to the annihilator, the maximal DTET efficiency would be 12.3% for An, 14.5% for PhAn, and 22.5% for DPA at the given concentrations (Table 1, Supporting Information, Table S2, Figure S9–11), and these values exceed the estimated DTET efficiency (∼9.6%) occurring at the diffusion limit (Supporting Information, Section 5.3).

To obtain deeper insight into the intermolecular DTET, the [Fe(phtmeimb)_2_]PF_6_ (500 μM)/DPA (10 mM) pair was studied on transient absorption spectroscopy equipped with a femtosecond-pulsed laser at 532 nm (Supporting Information, Section 7.1). In the absence of DPA, the initially formed excited state absorption signal of [Fe(phtmeimb)_2_]PF_6_ maximized at 580 nm (Figure 2d)^75,98^ follows a monoexponential decay with a time constant of 1.44 ns (Supporting Information, Figure S24a), which agrees well with the decay kinetics at 445 nm (1.55 ns, Figure 2f, red traces) and the previously obtained luminescence lifetime (Figure 1b, upper panel, Supporting Information, Figure S7b). In the presence of 10 mM DPA, the transient absorption signal at 580 nm decays faster with a time constant of 1.11 ns (Supporting Information, Figure S24a). This corresponds to a DTET efficiency of 22.9%, which is identical to the DTET efficiency obtained from steady-state luminescence quenching with the same concentration of DPA (Table 1). This agreement implies that DTET seems to account exclusively for the luminescence quenching, and the above-mentioned preassociation does not lead to luminescence quenching. For such a multistep process, in which the DTET occurs following the preassociation between the Fe(III) complex and the anthracenes, simple static or dynamic quenching does not readily apply (Supporting Information, Section 7.1).^92,99^ For the reported [Fe(ImPP)_2_]^+^-sensitized photon upconversion, the maximum DTET efficiency is estimated to be ∼4%, with the assumption that DTET occurs at the diffusion limit of the employed solvent.^78^ For our [Fe(phtmeimb)_2_]PF_6_/DPA (10 mM) pair, the higher DTET efficiency of ∼23% obtained from the excited state quenching studies is ascribed to the preassociation that narrows the bimolecular distance and the longer luminescence lifetime of [Fe(phtmeimb)_2_]^+^ than [Fe(ImPP)_2_]^+^. Selective excitation of the [Fe(phtmeimb)_2_]PF_6_ (500 μM)/DPA (10 mM) pair with fs-pulsed laser at 532 nm leads to the formation of the spectral signature of ^3^DPA* at ∼445 nm (Figure 2e),^61,90^ which decays significantly slower than the transient absorption signal of neat [Fe(phtmeimb)_2_]PF_6_ solution at the same wavelength and reaches a plateau around 3 ns after the laser excitation (Figure 2f). Excitation of the [Fe(phtmeimb)_2_]PF_6_/DPA pair with a 532 nm ns-pulsed laser confirms the spectral signature of ^3^DPA* (Figure 2c), which decays with a time constant of 2.4 μs under the measurement conditions (Supporting Information, Figure S29c).^81^ These spectroscopic investigations indicate DTET from the ^2^LMCT excited state of the Fe(III) complex to the T_1_ state of DPA.

DTET has been recently unambiguously evidenced with luminescent Cr(III) complexes with spin-flip doublet excited states,^61,103^ the above-mentioned [Fe(ImPP)_2_]^+^,^78^ and organic π-radical photosensitizers.^104,105^ For the [Fe(phtmeimb)_2_]PF_6_/DPA pair, the spin multiplicity of the Fe(III) complex remains doublet during the DTET, whereas the spin state of DPA changes from a singlet ground state (S_0_) to the triplet excited state (Figure 2g). This is in contrast to most triplet and doublet sensitizers, whose spin multiplicity is typically changed upon energy transfer to an acceptor (Supporting Information, Section 5.4). Importantly, the overall spin multiplicity of the donor–acceptor pair remains unchanged after the DTET, which makes the energy transfer a spin-allowed process according to the Wigner spin conservation rule.^106−108^ The overall spin multiplicity is often discussed in photoinduced electron transfer and charge recombination of a donor–acceptor pair caged in solvent molecules,^83,109,110^ but only very few examples have applied this concept for energy transfer.^108^ A representative example is the triplet-quartet energy transfer from a Re(I)-based energy donor (^3^MLCT → ^1^MLCT) to a Cr(III)-based energy acceptor (^4^A_2_ → ^4^T_2_), and this process is rationalized with the Förster theory.^108^ However, this seems not to be our case due to a lack of spectral overlap between the donor emission and the acceptor absorption, which is a crucial parameter for Förster-type energy transfer.^111,112^ Instead, the Dexter-type electron exchange mechanism seems plausible for the Fe(III)/anthracenes pairs (Figure 2g, blue arrows, Supporting Information, Section 5.4), similar to the DTET reported for Cr(III)/anthracene pairs.^61,103^ The preassociation between the Fe(III) complex and DPA makes the strong distance dependence of Dexter energy transfer and the short ^2^LMCT excited state lifetime less challenging. The spin-allowed and thermodynamically feasible DTET in the Fe(III)/anthracenes pairs provides an important fundamental basis for using photoactive Fe(III) complexes for energy transfer-based applications, such as photon upconversion.

### Sensitized TTA Upconversion

**Figure 3:**
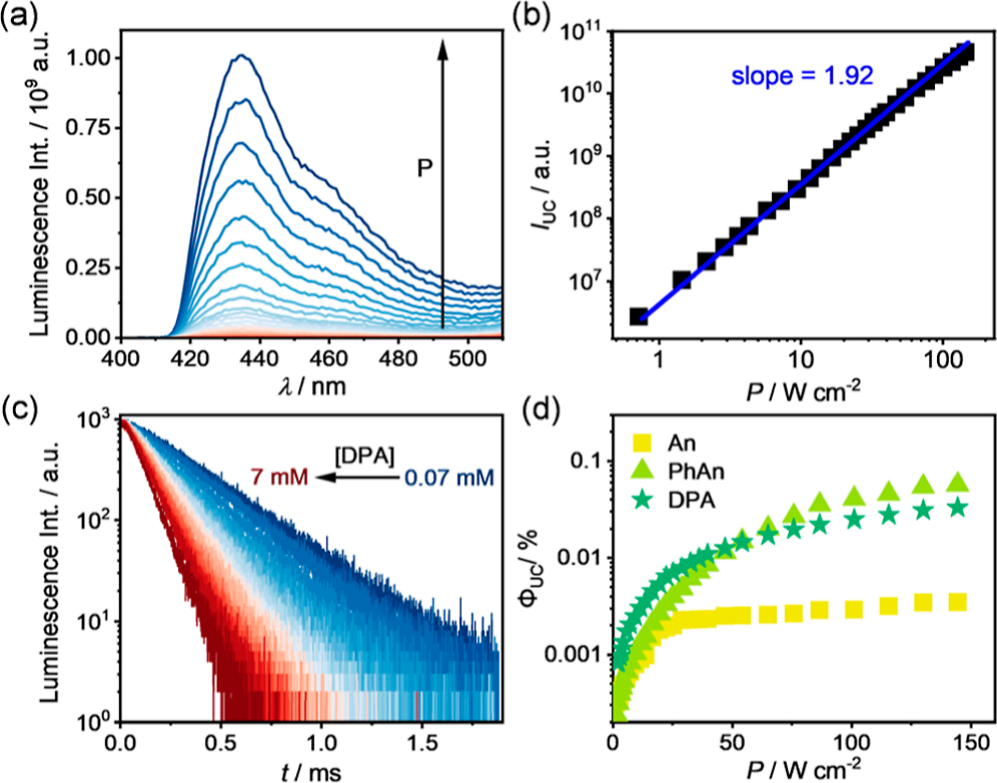
(a) Upconversion luminescence spectra of [Fe(phtmeimb)_2_]PF_6_ (40 μM)/DPA (10 mM) in aerated DMSO at 20 °C, excited with a green 532 nm cw-laser at different powers (1 mW to 200 mW). (b) Excitation power density dependence of the upconversion luminescence integral from 410 to 510 nm extracted from a) as a log–log plot. A linear fit of the plot gives a slope of 1.92, indicating a biphotonic nature of the upconversion. (c) Normalized upconversion luminescence decay at 430 nm recorded from a solution containing [Fe(phtmeimb)_2_]PF_6_ (40 μM) with different concentrations of DPA (0.07–7 mM) in aerated DMSO at 20 °C. Excitation occurred with the 532 nm laser (200 mW) with a pulse width of 250 μs. (d) Upconversion luminescence quantum yield (Φ_UC_) obtained with [Fe(phtmeimb)_2_]PF6 (40 μM) and different anthracenes [An (20 mM), PhAn (20 mM), DPA (10 mM)] in aerated DMSO at 20 °C as a function of the excitation power density from 0.72 to 144 W cm^–2^ using a tunable 532 nm cw-laser. For all upconversion luminescence measurements, a 495 nm long pass filter was placed between the laser and the samples.

Selective excitation of the Fe(III) (40 μM)/DPA (10 mM) pair in aerated DMSO at 532 nm leads to an upconversion luminescence from the S_1_ state of DPA with a maximum at 435 nm, which corresponds to a pseudo anti-Stokes shift Δ*E* of 0.52 eV, and a higher Δ*E* of 0.72 eV is reached with An as the annihilator (Table 1). The first fluorescence band of DPA at 413 nm becomes an emission shoulder due to the reabsorption phenomena that occurred at the high annihilator concentrations.^113−115^ In the absence of the Fe(III) complex, no upconversion luminescence is observed from the neat solutions of the anthracenes under identical measurement conditions (Supporting Information, Section 8.2). The upconversion luminescence from DPA is dramatically enhanced by increasing the excitation power density (Figure 3a). The integrated upconversion luminescence *I*_410–510_ follows a quadratic dependence on the excitation power density (slope of 1.92) due to the biphotonic nature of TTA (second-order reaction).^116^ No upconversion saturation is reached for the Fe(III)/DPA pair under our conditions (Figure 3b, Supporting Information, Section 8.5), despite the high excitation power densities used in this study, likely due to the low DTET efficiency and therefore low concentrations of ^3^DPA*.^117,118^

The upconversion luminescence of DPA decays with a time constant τ_UC_ ranging from 110 to 304 μs, which decreases at higher concentrations of DPA, as a consequence of more frequent encounters between the triplet excited DPA (Figure 3c, Supporting Information, Section 8.5).^117^ These τ_UC_ values exceed the prompt fluorescence lifetime of 1.10 ns for DPA by approximately 5 orders of magnitude (Supporting Information, Figures S6b and 42b). Using [Fe(phtmeimb)_2_]PF_6_ (40 μM in DMSO) as the reference, the reachable upconversion quantum yield Φ_UC_ was determined twice independently to 0.03% (relative to a theoretical limit of 50%)^119^ for the Fe(III) (40 μM)/DPA (10 mM) pair in aerated DMSO under our conditions. This is lower than the Φ_UC_ of 0.06% obtained from the Fe(III) (40 μM)/PhAn (20 mM) pair, presumably due to the lower concentration of DPA limited by the solubility in DMSO (Table 1, Supporting Information, and Section 8.3–8.5). These values are found in the same magnitude as the Φ_UC_ of ∼0.02% reported for the [Fe(ImPP)_2_]^+^-sensitized photon upconversion in deaerated solution at room temperature,^78^ even our Fe(III)/anthracene pairs show higher DTET efficiencies as mentioned previously. This is likely because our anthracene-based annihilators form excimers at such high concentrations (Figure 2a, Supporting Information, Section 8.2), which diminishes the upconversion quantum yield.^8,120^ The even lower Φ_UC_ of 0.003% obtained from the Fe(III) (40 μM)/An (20 mM) pair is attributable to the low fluorescence quantum yield and the photodimerization reaction of the singlet excited An.^61,90^ These generally low Φ_UC_ values are mainly attributed to the low DTET efficiency, as previously discussed.

### Mediator-Enhanced Photon Upconversion

**Figure 4:**
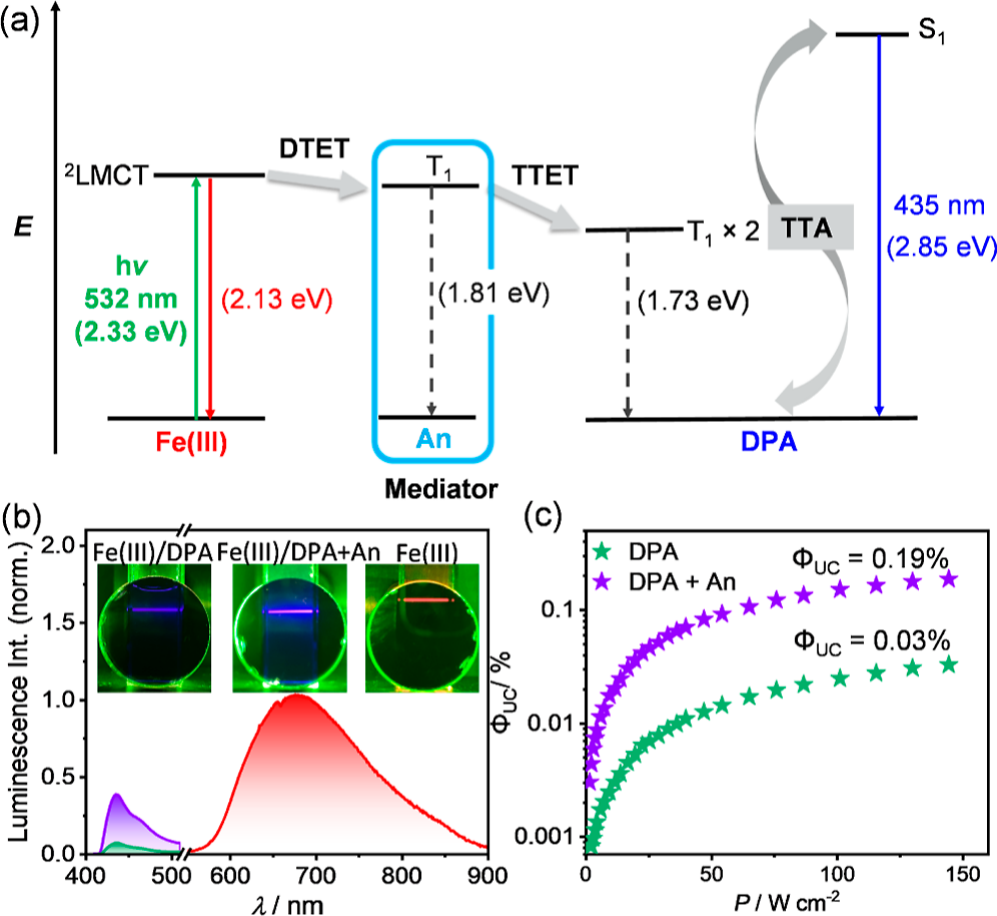
(a) Energy-level diagram for the sTTA-UC occurring in the [Fe(phtmeimb)_2_]PF_6_/DPA pair with An as the mediator. DTET: doublet-triplet energy transfer; TTET: triplet–triplet energy transfer; and TTA: triplet–triplet annihilation. (b) Upconversion luminescence spectra of the [Fe(phtmeimb)_2_]PF_6_ (40 μM)/DPA (10 mM) pair in the absence (green traces) and presence (purple traces) of An as the mediator in aerated DMSO at 20 °C; inset: corresponding photographs of the upconversion samples in the absence and presence of An. (c) Upconversion luminescence quantum yield (Φ_UC_) of [Fe(phtmeimb)_2_]PF_6_ (40 μM)/DPA (10 mM) in the absence and presence of An (10 mM) as the mediator in aerated DMSO at 20 °C as a function of the excitation power density (532 nm cw-laser). A 495 nm long pass filter was placed between the laser and the samples.

To enhance the energy transfer efficiency between the Fe(III) complex and DPA, a mediator based on an organic chromophore is introduced, which features a triplet state with a longer excited lifetime than the photosensitizer and an energy level between the ^2^LMCT and the T_1_ states. The mediator can be introduced either via a direct mixing fashion with the sensitizer/annihilator pair^121^ or via Coulombic interactions with the ionic sensitizer.^122,123^ Addition of An (10 mM) to the [Fe(phtmeimb)_2_]PF_6_ (40 μM)/DPA (10 mM) pair leads to significantly enhanced upconversion luminescence from DPA, and no fluorescence features from An are observed (Figure 4b, Supporting Information, Section 9.2). With the T_1_ state energy of 1.81 eV (Figure 1a, Supporting Information, Figure S3), An acts as an energy acceptor for the DTET from the Fe(III) complex and simultaneously as an energy donor with a long-lived T_1_ state for TTET to DPA (Figures 4a and S31). The μs-scaled lifetime of ^3^An* is favorable for a more efficient population of the T_1_ state of DPA than that sensitized solely by the Fe(III) complex, which leads to a maximal Φ_UC_ of 0.19% under our conditions (Figure 4c). This value exceeds the reachable Φ_UC_ in the absence of An by a factor of 6.3 under identical conditions, and the use of PhAn as a mediator improves the Φ_UC_ by a factor of 5.3 (Table 1, Supporting Information, Section 9.3). A Φ_UC_ of ∼0.2% is moderate, but this is comparable to those sensitized by Ru(II)^122^ and Os(II)^30,32^ complexes, and this exceeds the reported Φ_UC_ value sensitized by [Fe(ImPP)_2_]^+^ by one order of magnitude.^78^

For the [Fe(phtmeimb)_2_]PF_6_/PhAn pair, however, the presence of An as the mediator lowers the Φ_UC_ value (Table 1, Supporting Information, Section 9.1). This is because the reverse TTET from the T_1_ state of PhAn to that of An is thermodynamically feasible due to their small energy gap,^90^ as evidenced by the significantly elongated T_1_ lifetime of PhAn from 2.1 to 81.3 μs by the addition of An (Supporting Information, Figure S26c and 27c). As a competing deactivation pathway to TTA, strong reserve TTET accounts likely for the observed drop of the Φ_UC_ and τ_UC_ values (Table 1, Supporting Information, Figure S28, Section 8.4 and 9.1). Evidently, the energy level of the mediator is a crucial parameter for the upconversion performance.

The [Fe(phtmeimb)_2_]PF_6_/DPA pair in aerated DMSO shows high photorobustness, as evidenced by the ∼85% retaining upconversion luminescence after two-hour continuous irradiation with a 532 nm cw-laser at the maximal power density of 144 W cm^–2^, and this is not affected by the presence of the An mediator (Supporting Information, Section 10). The high photostability coupled with a moderate upconversion efficiency of ∼0.2% makes the luminescent Fe(III) complex promising for sustainable conversion of light energy.

### UC-Driving Photocatalytic Radical Polymerization

**Figure 5:**
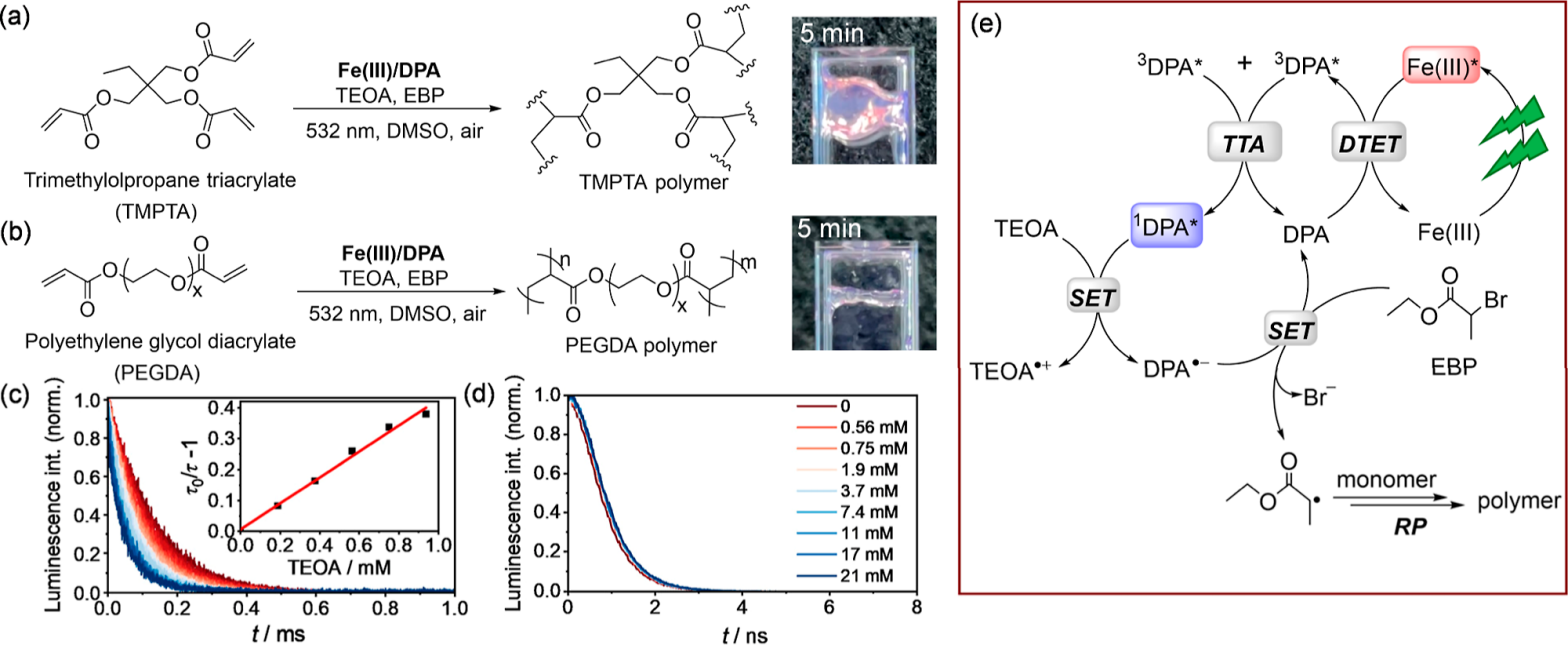
Photopolymerization reaction schemes of (a) trimethylolpropane triacrylate (TMPTA) and (b) polyethylene glycol diacrylate (PEGDA) with their respective polymerization images at the indicated irradiation time (right). (c) Normalized upconversion luminescence decays of the Fe(III) (70 μM)/DPA (7 mM) upconversion pair at 430 nm in the presence of triethanolamine (TEOA) with different concentrations in aerated DMSO at 20 °C. Excitation occurred with a 532 nm laser (200 mW) with a pulse width of 250 μs. Inset: linear Stern–Volmer plots derived from the lifetime data in (c), giving a quenching rate constant of *k*_q_ = 3.20 × 10^6^ M^–1^ s^–1^. (d) Normalized prompt fluorescence decays of DPA (10 μM) at 430 nm in the presence of TEOA with different concentrations. Excitation occurred with a 390 nm pulsed LED. (e) Plausible photocatalytic polymerization mechanism employing the Fe(III)/DPA upconversion pair under green light irradiation. DTET: doublet-triplet energy transfer; TTA: triplet–triplet annihilation; SET: single electron transfer; and RP: radical polymerization.

Acrylate polymers are widely used in industry and biomedicines due to their light transparency, elasticity, weatherability, and high biocompatibility.^124,125^ Photocatalytic radical polymerization allows the synthesis of these polymers in a spatially and temporally controlled manner by light,^126−128^ and using low-energetic visible light to drive these reactions is particularly attractive for 3D printing.^11,46,47^ DPA is a well-known blue emitter for sTTA-UC,^16,129^ but the utilization as a photoredox catalyst remains underexplored, and the extremely few examples require high-energy irradiation photons at ∼395 nm.^130,131^ DPA radical anion has a strong reducing power *E*_1/2_^0^ (DPA/DPA^•–^) = −1.94 V vs SCE in the ground state,^90^ which enables the reduction of an alkyl halide-based initiator, such as ethyl 2-bromopropionate (EBP) shown in Figure 5e.^132−134^ We decide to use our Fe(III)/DPA upconversion pair for green-light-driving photocatalytic radical polymerization of acrylate monomers, including trimethylolpropane triacrylate (TMPTA) and poly(ethylenglycol)diacrylate (PEGDA) (Figure 5a and b, Supporting Information, Section 11).

The DPA radical anion (DPA^•–^) can be formed via photoinduced electron transfer with an electron donor.^131^ The singlet excited state redox potential of DPA is estimated to be *E*^0^(^1^DPA*/DPA^•–^) = 1.06 V vs SCE (Supporting Information, Section 11.1), which allows exothermic reductive quenching by TEOA (*E*_1/2_^0^ (TEOA^•+^/TEOA) = 0.90 V vs SCE)^90^ with a driving force Δ*G*_ET_ = −0.16 eV. This is evidenced by the quenched upconversion luminescence lifetime of DPA by TEOA with a quenching rate constant *k*_q_ of 3.20 × 10^6^ M^–1^ s^–1^ (Figure 5c), which is 3 orders of magnitude below the diffusion limit of DMSO at 20 °C (*k*_diff._ = 2.9 × 10^9^ M^–1^ s^–1^).^90^ This low *k*_q_ value and the small driving force Δ*G*_ET_ fit qualitatively to the Rhem-Weller trend, in which the rate constants for electron transfer with a small driving force (Δ*G*_ET_ near 0) fall typically in the regime of ∼10^6^ to 10^7^ M^–1^ s^–1^.^45,109,110,135−137^ However, for the prompt fluorescence of DPA, no quenching of the decay kinetics was observed in the presence of TEOA (Figure 5d). This is because the upconversion luminescence of DPA decays ∼100,000 times slower than the prompt fluorescence of DPA (see previous discussion). For the ^1^DPA* accessed via sTTA-UC, the long luminescence lifetime of ∼130 μs enables a quenching efficiency of ∼62% at a TEOA concentration of 70 mM used below for photopolymerization (Figure 5c, Supporting Information, Section 11.1). This quenching process is kinetically hindered for the prompt ^1^DPA*, as a consequence of the short fluorescence lifetime (Figure 5d, Supporting Information, and Section 11.1). This agrees well with our previous finding that long excited state lifetimes boost the excited state reactivity for electron- or energy transfer with small driving forces and slow reaction rates.^45^

Figure 5e illustrates the proposed mechanism for photocatalytic polymerization using the Fe(III)/DPA upconversion pair and green irradiation light. In an aerated DMSO solution containing the acrylate monomer TMPTA or PEGDA, EBP as the initiator, TEOA as the additive, and the Fe(III)/DPA pair, irradiation with a 532 nm green laser (200 mW) forms the ^1^DPA* in the laser beam via sTTA-UC. Upon encounter with TEOA, the ^1^DPA* is reductively quenched, giving a TEOA radical cation and a DPA radical anion. The formed DPA^•–^ reduces subsequently the C–Br bond of the EBP initiator, which gives a bromide ion and a propagating alkyl radical for radical polymerization (Figure 5e, Supporting Information, Figure S51).^132,133^ Meanwhile, the catalytic cycle is closed upon recovering DPA to the ground state. The introduction of An as a mediator to the Fe(III)/DPA pair led to a significantly slower reaction rate, likely due to the photoredox noninnocent ^3^An* that induces unwanted side reactions (Supporting Information, Section 11.1). After irradiation of the reaction mixtures in a dark environment for 5 min with green light, the liquid solutions transformed into a freestanding gel for the TMPTA polymer (Figure 5a, right image) and a polymer stick along the light beam for the PEGDA polymer (Figure 5b, right image). The latter indicates a spatial control of the polymer synthesis, which is highly attractive for stereolithographic 3D printing.^2,11,47,53^ With a longer irradiation time of up to 60 min, the reaction mixture containing PEGDA was converted into a freestanding polymer gel (Supporting Information, Figure S54). Control experiments, in which either the Fe(III) complex, DPA, TEOA, or light was omitted, did not yield any polymers (Supporting Information, Section 11.2, 11.3, and Figure S53 and 54).

Visible light-mediated organophotocatalysts are receiving substantial attention for polymer synthesis,^134,138−141^ but they often rely on high-energy purple or blue irradiation light, whereas our Fe(III)/DPA upconversion pair benefits from the low-energy green irradiation light and high excited state reactivity toward efficient photopolymerizations (Supporting Information, Section 11.1). Current UC-driving photopolymerization relies mostly on a highly emissive upconverted state, which undergoes radiative or nonradiative energy transfer to activate a photoinitiator for polymerization.^11,43,46,49−53^ Only very few examples have used sTTA-UC for photoredox catalytic polymerization.^34,42,54^ The proof-of-principle experiment in Figure 5 performs a new strategy for initiating photopolymerization via sTTA-UC, which benefits from (i) the low-energy visible light irradiation, (ii) the photoredox catalytic properties of the singlet-excited annihilator, and (iii) the long-lived nature of the upconverted excited state with enhanced excited state reactivity. This makes a fundamentally important step toward efficient photochemical reactions via photon upconversion.

Examples of using photoactive first–row transition metal complexes for UC-driving photopolymerizations remain very few,^43,54^ as previously mentioned. A Cr(0) isocyanide complex was reported to sensitize red-to-blue upconversion with an anthracene derivative and initiate radical polymerization, in which a photoinitiator was activated by absorbing the upconversion luminescence.^43^ This approach performs a clean polymer synthesis with separated vessels for polymer reaction and for photon upconversion with red irradiation light, but the reaction requires multiple hours of laser irradiation. Another example involves a Zn(II) meso-tetraphenylporphyrin (ZnTPP) complex, which performs homomolecular photon upconversion in solution.^54^ The upconverted, highly reducing S_2_ excited state undergoes single-electron transfer with the monomers and initiates radical polymerizations in deaerated solutions with green light. Our Fe(III)/DPA upconversion pair, which allows rapid initiation of polymerization reactions under aerobic conditions (Supporting Information, Section 11.3), is seen furthermore as a competitive candidate for catalyzing future polymer synthesis.

## Conclusions

Photoactive iron complexes are attracting enormous attention for photoredox catalysis^77,81−86,142−144^ and photoinduced charge separation,^79,80^ but they remain underexplored for energy transfer and photon upconversion. This is mainly restricted by the short excited state lifetime, up to a few nanoseconds. Using the low-spin 3d^5^ [Fe(phtmeimb)_2_]PF_6_ as the photosensitizer, which features a luminescent ^2^LMCT excited state, we report here a few green-to-blue/purple upconversion pairs with anthracene-based annihilators. The Fe(III) complex and the anthracenes are preassociated in their ground states via dynamic π–π interactions, as evidenced by luminescence quenching studies and ^1^H NMR titration. Such preassociation is particularly beneficial for electron- or energy transfer involving a short-lived excited state.^77,92,97^

Assisted by the preassociation, Dexter-type electron exchange mechanism enables DTET from the ^2^LMCT excited state of the Fe(III) complex to the T_1_ state of the anthracenes, with perpetually unchanged overall spin multiplicity of the donor–acceptor pair according to the Wigner spin conservation rule (Figure 2).^106−108^ DTET from a photoactive Fe(III) complex to an organic chromophore remains fundamentally underexplored, and we try to uncover this aspect in this study with advanced spectroscopic techniques and spin discussions. Based on this DTET, an intramolecular “doublet-triplet reservoir” between the ^2^LMCT excited state of a photoactive Fe(III) complex and the T_1_ state of an attached organic chromophore is in principle feasible, which seems a highly promising strategy for extending the ^2^LMCT excited state lifetime of the photoactive Fe(III) complexes. Such a strategy has been successfully applied to many coordination compounds based on precious metals, such as Ru(II),^25,45,145−150^ Re(I),^151,152^ Os(II),^30,153,154^ Ir(III),^28,155^ Pt(II),^156^ and more abundant Cu(I),^157−159^ leading to essentially elongated triplet excited state lifetimes.

DTET from [Fe(phtmeimb)_2_]PF_6_ to different anthracenes allows consequently green-to-blue/purple upconversion with an efficiency Φ_UC_ ranging from 0.003% to 0.06% in aerated DMSO, and the delayed fluorescence shows a μs-scaled lifetime (Figure 3). For the Fe(III)/DPA pair, the addition of An as a mediator leads to a 6-fold enhancement of the Φ_UC_ to 0.19% in aerated DMSO at room temperature (Figure 4). This moderate upconversion efficiency and the high photorobustness promise the future of using the photoactive Fe(III) complex for sensitizing photon upconversion.

The Fe(III)/DPA upconversion pair furthermore enables efficient photopolymerization of acrylates with green light under aerobic conditions, in which the singlet excited state of DPA accessed via sTTA-UC undergoes single electron transfer and catalyzes the radical polymerizations of TMPTA and PEGDA, whereas this process is kinetically hindered with the prompt DPA (Figure 5). Our study offers an important strategy of using a photosensitizer based on low-cost iron for catalytically initiating radical polymerization with low irradiation energy. This is not merely an academic interest but does indeed bring iron a significant step forward in future applications.

## Supporting Information Available

Synthetic protocols and characterization data, description of equipment and methods, and supplementary spectroscopic data (PDF)

## References

[ref1] Dou Q.; Jiang L.; Kai D.; Owh C.; Loh X. J. Bioimaging and biodetection assisted with TTA-UC materials. Drug Discovery Today 2017, 22, 1400–1411. 10.1016/j.drudis.2017.04.003. 28433535

[ref2] Schloemer T.; Narayanan P.; Zhou Q.; Belliveau E.; Seitz M.; Congreve D. N. Nanoengineering Triplet-Triplet Annihilation Upconversion: From Materials to Real-World Applications. ACS Nano 2023, 17, 3259–3288. 10.1021/acsnano.3c00543. 36800310

[ref3] Richards B. S.; Hudry D.; Busko D.; Turshatov A.; Howard I. A. Photon Upconversion for Photovoltaics and Photocatalysis: A Critical Review. Chem. Rev. 2021, 121, 9165–9195. 10.1021/acs.chemrev.1c00034. 34327987

[ref4] Seo S. E.; Choe H.-S.; Cho H.; Kim H. i.; Kim J. H.; Kwon O. S. Recent advances in materials for and applications of triplet–triplet annihilation-based upconversion. J. Mater. Chem. C 2022, 10, 4483–4496. 10.1039/d1tc03551g.

[ref5] Kondakov D. Y. Triplet-triplet annihilation in highly efficient fluorescent organic light-emitting diodes: current state and future outlook. Philos. Trans. R. Soc. A 2015, 373, 20140321 10.1098/rsta.2014.0321. 25987574

[ref6] Glaser F.; Kerzig C.; Wenger O. S. Sensitization-initiated electron transfer via upconversion: mechanism and photocatalytic applications. Chem. Sci. 2021, 12, 9922–9933. 10.1039/D1SC02085D. 34349964 PMC8317647

[ref7] Zhou J.; Liu Q.; Feng W.; Sun Y.; Li F. Upconversion luminescent materials: advances and applications. Chem. Rev. 2015, 115, 395–465. 10.1021/cr400478f. 25492128

[ref8] Uji M.; Zähringer T. J. B.; Kerzig C.; Yanai N. Visible-to-UV Photon Upconversion: Recent Progress in New Materials and Applications. Angew. Chem., Int. Ed. 2023, 62, e202301506 10.1002/anie.202301506. 36882372

[ref9] Bharmoria P.; Bildirir H.; Moth-Poulsen K. Triplet-triplet annihilation based near infrared to visible molecular photon upconversion. Chem. Soc. Rev. 2020, 49, 6529–6554. 10.1039/D0CS00257G. 32955529

[ref10] Pérez-Ruiz R. Photon Upconversion Systems Based on Triplet-Triplet Annihilation as Photosensitizers for Chemical Transformations. Top. Curr. Chem. 2022, 380, 23 10.1007/s41061-022-00378-6. 35445872

[ref11] Sanders S. N.; Schloemer T. H.; Gangishetty M. K.; Anderson D.; Seitz M.; Gallegos A. O.; Stokes R. C.; Congreve D. N. Triplet fusion upconversion nanocapsules for volumetric 3D printing. Nature 2022, 604, 474–478. 10.1038/s41586-022-04485-8. 35444324

[ref12] Parker C. A.; Hatchard C. G. Sensitized Anti-Stokes Delayed Fluorescence. Proc. Chem. Soc. 1962, 386–387.

[ref13] Parker C. A.; Hatchard C. G.; Joyce T. A. Selective and Mutual Sensitization of Delayed Fluorescence. Nature 1965, 205, 1282–1284. 10.1038/2051282a0.

[ref14] Singh-Rachford T. N.; Castellano F. N. Photon upconversion based on sensitized triplet–triplet annihilation. Coord. Chem. Rev. 2010, 254, 2560–2573. 10.1016/j.ccr.2010.01.003.

[ref15] Gray V.; Moth-Poulsen K.; Albinsson B.; Abrahamsson M. Towards efficient solid-state triplet–triplet annihilation based photon upconversion: Supramolecular, macromolecular and self-assembled systems. Coord. Chem. Rev. 2018, 362, 54–71. 10.1016/j.ccr.2018.02.011.

[ref16] Fan C.; Wei L.; Niu T.; Rao M.; Cheng G.; Chruma J. J.; Wu W.; Yang C. Efficient Triplet-Triplet Annihilation Upconversion with an Anti-Stokes Shift of 1.08 eV Achieved by Chemically Tuning Sensitizers. J. Am. Chem. Soc. 2019, 141, 15070–15077. 10.1021/jacs.9b05824. 31469266

[ref17] Duan P.; Yanai N.; Kimizuka N. Photon upconverting liquids: matrix-free molecular upconversion systems functioning in air. J. Am. Chem. Soc. 2013, 135, 19056–19059. 10.1021/ja411316s. 24328197

[ref18] Zach P. W.; Freunberger S. A.; Klimant I.; Borisov S. M. Electron-Deficient Near-Infrared Pt(II) and Pd(II) Benzoporphyrins with Dual Phosphorescence and Unusually Efficient Thermally Activated Delayed Fluorescence: First Demonstration of Simultaneous Oxygen and Temperature Sensing with a Single Emitter. ACS Appl. Mater. Interfaces 2017, 9, 38008–38023. 10.1021/acsami.7b10669. 29016109

[ref19] Ogawa T.; Yanai N.; Monguzzi A.; Kimizuka N. Highly Efficient Photon Upconversion in Self-Assembled Light-Harvesting Molecular Systems. Sci. Rep. 2015, 5, 10882 10.1038/srep10882. 26057321 PMC4460878

[ref20] Dzebo D.; Moth-Poulsen K.; Albinsson B. Robust triplet-triplet annihilation photon upconversion by efficient oxygen scavenging. Photochem. Photobiol. Sci. 2017, 16, 1327–1334. 10.1039/c7pp00201g. 28726960

[ref21] Nishimura N.; Gray V.; Allardice J. R.; Zhang Z.; Pershin A.; Beljonne D.; Rao A. Photon Upconversion from Near-Infrared to Blue Light with TIPS-Anthracene as an Efficient Triplet–Triplet Annihilator. ACS Mater. Lett. 2019, 1, 660–664. 10.1021/acsmaterialslett.9b00287.

[ref22] Gharaati S.; Wang C.; Förster C.; Weigert F.; Resch-Genger U.; Heinze K. Triplet-Triplet Annihilation Upconversion in a MOF with Acceptor-Filled Channels. Chem.—Eur. J. 2020, 26, 1003–1007. 10.1002/chem.201904945. 31670422 PMC7027809

[ref23] Boutin P. C.; Ghiggino K. P.; Kelly T. L.; Steer R. P. Photon Upconversion by Triplet–Triplet Annihilation in Ru(bpy)_3_- and DPA-Functionalized Polymers. J. Phys. Chem. Lett. 2013, 4, 4113–4118. 10.1021/jz402311j.

[ref24] Wu W.; Ji S.; Wu W.; Shao J.; Guo H.; James T. D.; Zhao J. Ruthenium(II)-polyimine-coumarin light-harvesting molecular arrays: design rationale and application for triplet-triplet-annihilation-based upconversion. Chem.—Eur. J. 2012, 18, 4953–4964. 10.1002/chem.201101377. 22407570

[ref25] Kerzig C.; Wenger O. S. Sensitized triplet-triplet annihilation upconversion in water and its application to photochemical transformations. Chem. Sci. 2018, 9, 6670–6678. 10.1039/C8SC01829D. 30310600 PMC6115628

[ref26] Peng J.; Jiang X.; Guo X.; Zhao D.; Ma Y. Sensitizer design for efficient triplet-triplet annihilation upconversion: annihilator-appended tris-cyclometalated Ir(III) complexes. Chem. Commun. 2014, 50, 7828–7830. 10.1039/c4cc01465k. 24909317

[ref27] Sun J.; Zhong F.; Yi X.; Zhao J. Efficient enhancement of the visible-light absorption of cyclometalated Ir(III) complexes triplet photosensitizers with Bodipy and applications in photooxidation and triplet-triplet annihilation upconversion. Inorg. Chem. 2013, 52, 6299–6310. 10.1021/ic302210b. 23327589

[ref28] Li H.; Wang C.; Glaser F.; Sinha N.; Wenger O. S. Metal-Organic Bichromophore Lowers the Upconversion Excitation Power Threshold and Promotes UV Photoreactions. J. Am. Chem. Soc. 2023, 145, 11402–11414. 10.1021/jacs.3c02609. 37186558 PMC10214436

[ref29] Amemori S.; Sasaki Y.; Yanai N.; Kimizuka N. Near-Infrared-to-Visible Photon Upconversion Sensitized by a Metal Complex with Spin-Forbidden yet Strong S_0_-T_1_ Absorption. J. Am. Chem. Soc. 2016, 138, 8702–8705. 10.1021/jacs.6b04692. 27354325

[ref30] Liu D.; Zhao Y.; Wang Z.; Xu K.; Zhao J. Exploiting the benefit of S0--> T1 excitation in triplet-triplet annihilation upconversion to attain large anti-stokes shifts: tuning the triplet state lifetime of a tris(2,2’-bipyridine) osmium(II) complex. Dalton Trans. 2018, 47, 8619–8628. 10.1039/C7DT04803C. 29512677

[ref31] Sasaki Y.; Amemori S.; Kouno H.; Yanai N.; Kimizuka N. Near infrared-to-blue photon upconversion by exploiting direct S–T absorption of a molecular sensitizer. J. Mater. Chem. C 2017, 5, 5063–5067. 10.1039/C7TC00827A.

[ref32] Wei Y.; Zheng M.; Chen L.; Zhou X.; Liu S. Near-infrared to violet triplet-triplet annihilation fluorescence upconversion of Os(II) complexes by strong spin-forbidden transition. Dalton Trans. 2019, 48, 11763–11771. 10.1039/C9DT02276G. 31298244

[ref33] Wei Y.; Li Y.; Zheng M.; Zhou X.; Zou Y.; Yang C. Simultaneously High Upconversion Efficiency and Large Anti-Stokes Shift by Using Os(II) Complex Dyad as Triplet Photosensitizer. Adv. Opt. Mater. 2020, 8, 1902157 10.1002/adom.201902157.

[ref34] Ravetz B. D.; Pun A. B.; Churchill E. M.; Congreve D. N.; Rovis T.; Campos L. M. Photoredox catalysis using infrared light via triplet fusion upconversion. Nature 2019, 565, 343–346. 10.1038/s41586-018-0835-2. 30651612 PMC6338432

[ref35] Huang L.; Wu W.; Li Y.; Huang K.; Zeng L.; Lin W.; Han G. Highly Effective Near-Infrared Activating Triplet-Triplet Annihilation Upconversion for Photoredox Catalysis. J. Am. Chem. Soc. 2020, 142, 18460–18470. 10.1021/jacs.0c06976. 33074671

[ref36] Gisbertz S.; Reischauer S.; Pieber B. Overcoming limitations in dual photoredox/nickel-catalysed C–N cross-couplings due to catalyst deactivation. Nat. Catal. 2020, 3, 611–620. 10.1038/s41929-020-0473-6.

[ref37] Huang L.; Zeng L.; Chen Y.; Yu N.; Wang L.; Huang K.; Zhao Y.; Han G. Long wavelength single photon like driven photolysis via triplet triplet annihilation. Nat. Commun. 2021, 12, 122 10.1038/s41467-020-20326-6. 33402702 PMC7785739

[ref38] Ayare P. J.; Watson N.; Helton M. R.; Warner M. J.; Dilbeck T.; Hanson K.; Vannucci A. K. Molecular Z-Scheme for Solar Fuel Production via Dual Photocatalytic Cycles. J. Am. Chem. Soc. 2022, 144, 21568–21575. 10.1021/jacs.2c08462. 36394978

[ref39] Glaser F.; Wenger O. S. Red Light-Based Dual Photoredox Strategy Resembling the Z-Scheme of Natural Photosynthesis. JACS Au 2022, 2, 1488–1503. 10.1021/jacsau.2c00265. 35783177 PMC9241018

[ref40] Glaser F.; Wenger O. S. Sensitizer-controlled photochemical reactivity via upconversion of red light. Chem. Sci. 2022, 14, 149–161. 10.1039/D2SC05229F. 36605743 PMC9769107

[ref41] Jin J.; Yu T.; Chen J.; Hu R.; Yang G.; Zeng Y.; Li Y. Recent advances of triplet–triplet annihilation upconversion in photochemical transformations. Curr. Opin. Green Sustainable Chem. 2023, 43, 100841 10.1016/j.cogsc.2023.100841.

[ref42] Liang W.; Nie C.; Du J.; Han Y.; Zhao G.; Yang F.; Liang G.; Wu K. Near-infrared photon upconversion and solar synthesis using lead-free nanocrystals. Nat. Photonics 2023, 17, 346–353. 10.1038/s41566-023-01156-6.

[ref43] Wang C.; Wegeberg C.; Wenger O. S. First-Row d^6^ Metal Complex Enables Photon Upconversion and Initiates Blue Light-Dependent Polymerization with Red Light. Angew. Chem., Int. Ed. 2023, 62, e202311470 10.1002/anie.202311470. 37681516

[ref44] Bertrams M. S.; Hermainski K.; Morsdorf J. M.; Ballmann J.; Kerzig C. Triplet quenching pathway control with molecular dyads enables the identification of a highly oxidizing annihilator class. Chem. Sci. 2023, 14, 8583–8591. 10.1039/D3SC01725G. 37592982 PMC10430750

[ref45] Hammecke H.; Fritzler D.; Vashistha N.; Jin P.; Dietzek-Ivanšić B.; Wang C. 100 μs Luminescence Lifetime Boosts the Excited State Reactivity of a Ruthenium(II)-Anthracene Complex in Photon Upconversion and Photocatalytic Polymerizations with Red Light. Chem.—Eur. J. 2024, e202402679 10.1002/chem.202402679. 39298687

[ref46] Bagheri A.; Jin J. Photopolymerization in 3D Printing. ACS Appl. Polym. Mater. 2019, 1, 593–611. 10.1021/acsapm.8b00165.

[ref47] Wei L.; Yang C.; Wu W. Recent advances of stereolithographic 3D printing enabled by photon upconversion technology. Curr. Opin. Green Sustainable Chem. 2023, 43, 100851 10.1016/j.cogsc.2023.100851.

[ref48] Xing J. F.; Zheng M. L.; Duan X. M. Two-photon polymerization microfabrication of hydrogels: an advanced 3D printing technology for tissue engineering and drug delivery. Chem. Soc. Rev. 2015, 44, 5031–5039. 10.1039/C5CS00278H. 25992492

[ref49] Ding C.; Wang J.; Zhang W.; Pan X.; Zhang Z.; Zhang W.; Zhu J.; Zhu X. Platform of near-infrared light-induced reversible deactivation radical polymerization: upconversion nanoparticles as internal light sources. Polym. Chem. 2016, 7, 7370–7374. 10.1039/C6PY01727D.

[ref50] Wang K.; Pena J.; Xing J. Upconversion Nanoparticle-Assisted Photopolymerization. Photochem. Photobiol. 2020, 96, 741–749. 10.1111/php.13249. 32115706

[ref51] Caron A.; Noirbent G.; Gigmes D.; Dumur F.; Lalevée J. Near-Infrared PhotoInitiating Systems: Photothermal versus Triplet–Triplet Annihilation-Based Upconversion Polymerization. Macromol. Rapid Commun. 2021, 42, 2100047 10.1002/marc.202100047. 33719083

[ref52] Limberg D. K.; Kang J. H.; Hayward R. C. Triplet-Triplet Annihilation Photopolymerization for High-Resolution 3D Printing. J. Am. Chem. Soc. 2022, 144, 5226–5232. 10.1021/jacs.1c11022. 35285620

[ref53] Wong J.; Wei S.; Meir R.; Sadaba N.; Ballinger N. A.; Harmon E. K.; Gao X.; Altin-Yavuzarslan G.; Pozzo L. D.; Campos L. M.; et al. Triplet Fusion Upconversion for Photocuring 3D-Printed Particle-Reinforced Composite Networks. Adv. Mater. 2023, 35, e2207673 10.1002/adma.202207673. 36594431

[ref54] Awwad N.; Bui A. T.; Danilov E. O.; Castellano F. N. Visible-Light-Initiated Free-Radical Polymerization by Homomolecular Triplet-Triplet Annihilation. Chem. 2020, 6, 3071–3085. 10.1016/j.chempr.2020.08.019.

[ref55] Peng J.; Guo X.; Jiang X.; Zhao D.; Ma Y. Developing efficient heavy-atom-free photosensitizers applicable to TTA upconversion in polymer films. Chem. Sci. 2016, 7, 1233–1237. 10.1039/C5SC03245H. 29910879 PMC5975838

[ref56] Yanai N.; Kimizuka N. New Triplet Sensitization Routes for Photon Upconversion: Thermally Activated Delayed Fluorescence Molecules, Inorganic Nanocrystals, and Singlet-to-Triplet Absorption. Acc. Chem. Res. 2017, 50, 2487–2495. 10.1021/acs.accounts.7b00235. 28930435

[ref57] Zhang X.; Wang Z.; Hou Y.; Yan Y.; Zhao J.; Dick B. Recent development of heavy-atom-free triplet photosensitizers: molecular structure design, photophysics and application. J. Mater. Chem. C 2021, 9, 11944–11973. 10.1039/D1TC02535J.

[ref58] Li J. K.; Zhang M. Y.; Zeng L.; Huang L.; Wang X. Y. NIR-Absorbing B,N-Heteroarene as Photosensitizer for High-Performance NIR-to-Blue Triplet-Triplet Annihilation Upconversion. Angew. Chem., Int. Ed. 2023, 62, e202303093 10.1002/anie.202303093. 37070679

[ref59] Zähringer T. J. B.; Moghtader J. A.; Bertrams M. S.; Roy B.; Uji M.; Yanai N.; Kerzig C. Blue-to-UVB Upconversion, Solvent Sensitization and Challenging Bond Activation Enabled by a Benzene-Based Annihilator. Angew. Chem., Int. Ed. 2023, 62, e202215340 10.1002/anie.202215340. PMC10108172 36398891

[ref60] Ye K.; Imran M.; Chen X.; Zhao J. Triplet Photosensitizers and Their Applications in Triplet–Triplet Annihilation Upconversion. ACS Appl. Opt. Mater. 2024, 2, 1803–1824. 10.1021/acsaom.4c00016.

[ref61] Wang C.; Reichenauer F.; Kitzmann W. R.; Kerzig C.; Heinze K.; Resch-Genger U. Efficient Triplet-Triplet Annihilation Upconversion Sensitized by a Chromium(III) Complex via an Underexplored Energy Transfer Mechanism. Angew. Chem., Int. Ed. 2022, 61, e202202238 10.1002/anie.202202238. PMC9322448 35344256

[ref62] Herr P.; Kerzig C.; Larsen C. B.; Häussinger D.; Wenger O. S. Mn(I) complexes with metal-to-ligand charge transfer luminescence and photoreactivity. Nat. Chem. 2021, 13, 956–962. 10.1038/s41557-021-00744-9. 34341527

[ref63] McCusker C. E.; Castellano F. N. Efficient Visible to Near-UV Photochemical Upconversion Sensitized by a Long Lifetime Cu(I) MLCT Complex. Inorg. Chem. 2015, 54, 6035–6042. 10.1021/acs.inorgchem.5b00907. 26035640

[ref64] Fayad R.; Bui A. T.; Shepard S. G.; Castellano F. N. Photochemical Upconversion in Water Using Cu(I) MLCT Excited States: Role of Energy Shuttling at the Micellar/Water Interface. ACS Appl. Energy Mater. 2020, 3, 12557–12564. 10.1021/acsaem.0c02492.

[ref65] Durandin N. A.; Isokuortti J.; Efimov A.; Vuorimaa-Laukkanen E.; Tkachenko N. V.; Laaksonen T. Efficient photon upconversion at remarkably low annihilator concentrations in a liquid polymer matrix: when less is more. Chem. Commun. 2018, 54, 14029–14032. 10.1039/C8CC07592A. 30488910

[ref66] Felter K. M.; Fravventura M. C.; Koster E.; Abellon R. D.; Savenije T. J.; Grozema F. C. Solid-State Infrared Upconversion in Perylene Diimides Followed by Direct Electron Injection. ACS Energy Lett. 2020, 5, 124–129. 10.1021/acsenergylett.9b02361. 31956696 PMC6958839

[ref67] Kübler J. A.; Pfund B.; Wenger O. S. Zinc(II) Complexes with Triplet Charge-Transfer Excited States Enabling Energy-Transfer Catalysis, Photoinduced Electron Transfer, and Upconversion. JACS Au 2022, 2, 2367–2380. 10.1021/jacsau.2c00442. 36311829 PMC9597861

[ref68] Mahmood Z.; Rehmat N.; Ji S.; Zhao J.; Sun S.; Di Donato M.; Li M.; Teddei M.; Huo Y. Tuning the Triplet Excited State of Bis(dipyrrin) Zinc(II) Complexes: Symmetry Breaking Charge Transfer Architecture with Exceptionally Long Lived Triplet State for Upconversion. Chemistry 2020, 26, 14912–14918. 10.1002/chem.202001907. 32567099

[ref69] Liu Y.; Persson P.; Sundstrom V.; Warnmark K. Fe N-Heterocyclic Carbene Complexes as Promising Photosensitizers. Acc. Chem. Res. 2016, 49, 1477–1485. 10.1021/acs.accounts.6b00186. 27455191

[ref70] Dierks P.; Vukadinovic Y.; Bauer M. Photoactive iron complexes: more sustainable, but still a challenge. Inorg. Chem. Front. 2022, 9, 206–220. 10.1039/D1QI01112J.

[ref71] Sinha N.; Wenger O. S. Photoactive Metal-to-Ligand Charge Transfer Excited States in 3d^6^ Complexes with Cr^0^, Mn^I^, Fe^II^, and Co^III^. J. Am. Chem. Soc. 2023, 145, 4903–4920. 10.1021/jacs.2c13432. 36808978 PMC9999427

[ref72] Witas K.; Nair S. S.; Maisuradze T.; Zedler L.; Schmidt H.; Garcia-Porta P.; Rein A. S. J.; Bolter T.; Rau S.; Kupfer S.; et al. Beyond the First Coordination Sphere horizontal line Manipulating the Excited-State Landscape in Iron(II) Chromophores with Protons. J. Am. Chem. Soc. 2024, 146, 19710–19719. 10.1021/jacs.4c00552. 38990184 PMC11273614

[ref73] Reuter T.; Zorn D.; Naumann R.; Klett J.; Förster C.; Heinze K. A Tetracarbene Iron(II) Complex with a Long-lived Triplet Metal-to-Ligand Charge Transfer State due to a Triplet-Triplet Barrier. Angew. Chem., Int. Ed. 2024, 63, e202406438 10.1002/anie.202406438. 38946322

[ref74] Chábera P.; Liu Y.; Prakash O.; Thyrhaug E.; Nahhas A. E.; Honarfar A.; Essén S.; Fredin L. A.; Harlang T. C.; Kjær K. S.; et al. A low-spin Fe(III) complex with 100-ps ligand-to-metal charge transfer photoluminescence. Nature 2017, 543, 695–699. 10.1038/nature21430. 28358064

[ref75] Kjær K. S.; Kaul N.; Prakash O.; Chábera P.; Rosemann N. W.; Honarfar A.; Gordivska O.; Fredin L. A.; Bergquist K.-E.; Häggström L.; et al. Luminescence and reactivity of a charge-transfer excited iron complex with nanosecond lifetime. Science 2019, 363, 249–253. 10.1126/science.aau7160. 30498167

[ref76] Steube J.; Kruse A.; Bokareva O. S.; Reuter T.; Demeshko S.; Schoch R.; Arguello Cordero M. A.; Krishna A.; Hohloch S.; Meyer F.; et al. Janus-type emission from a cyclometalated iron(III) complex. Nat. Chem. 2023, 15, 468–474. 10.1038/s41557-023-01137-w. 36849804 PMC10070185

[ref77] Ye Y.; Garrido-Barros P.; Wellauer J.; Cruz C. M.; Lescouëzec R.; Wenger O. S.; Herrera J. M.; Jiménez J. R. Luminescence and Excited-State Reactivity in a Heteroleptic Tricyanido Fe(III) Complex. J. Am. Chem. Soc. 2024, 146, 954–960. 10.1021/jacs.3c11517. 38156951 PMC10786067

[ref78] Wellauer J.; Ziereisen F.; Sinha N.; Prescimone A.; Velic A.; Meyer F.; Wenger O. S. Iron(III) Carbene Complexes with Tunable Excited State Energies for Photoredox and Upconversion. J. Am. Chem. Soc. 2024, 146, 11299–11318. 10.1021/jacs.4c00605. 38598280 PMC11046485

[ref79] Kaul N.; Lomoth R. The Carbene Cannibal: Photoinduced Symmetry-Breaking Charge Separation in an Fe(III) N-Heterocyclic Carbene. J. Am. Chem. Soc. 2021, 143, 10816–10821. 10.1021/jacs.1c03770. 34264638 PMC8397313

[ref80] Zhang M.; Johnson C. E.; Ilic A.; Schwarz J.; Johansson M. B.; Lomoth R. High-Efficiency Photoinduced Charge Separation in Fe(III)carbene Thin Films. J. Am. Chem. Soc. 2023, 145, 19171–19176. 10.1021/jacs.3c05404. 37616472 PMC10485928

[ref81] Aydogan A.; Bangle R. E.; Cadranel A.; Turlington M. D.; Conroy D. T.; Cauët E.; Singleton M. L.; Meyer G. J.; Sampaio R. N.; Elias B.; et al. Accessing Photoredox Transformations with an Iron(III) Photosensitizer and Green Light. J. Am. Chem. Soc. 2021, 143, 15661–15673. 10.1021/jacs.1c06081. 34529421

[ref82] De Kreijger S.; Ripak A.; Elias B.; Troian-Gautier L. Investigation of the Excited-State Electron Transfer and Cage Escape Yields Between Halides and a Fe(III) Photosensitizer. J. Am. Chem. Soc. 2024, 146, 10286–10292. 10.1021/jacs.4c02808. 38569088

[ref83] Ripak A.; Vega Salgado A. K.; Valverde D.; Cristofaro S.; de Gary A.; Olivier Y.; Elias B.; Troian-Gautier L. Factors Controlling Cage Escape Yields of Closed- and Open-Shell Metal Complexes in Bimolecular Photoinduced Electron Transfer. J. Am. Chem. Soc. 2024, 146, 22818–22828. 10.1021/jacs.4c08158. 39078742

[ref84] de Groot L. H. M.; Ilic A.; Schwarz J.; Wärnmark K. Iron Photoredox Catalysis-Past, Present, and Future. J. Am. Chem. Soc. 2023, 145, 9369–9388. 10.1021/jacs.3c01000. 37079887 PMC10161236

[ref85] Ilic A.; Strucker B. R.; Johnson C. E.; Hainz S.; Lomoth R.; Wärnmark K. Aminomethylations of electron-deficient compounds-bringing iron photoredox catalysis into play. Chem. Sci. 2024, 15, 12077–12085. 10.1039/D4SC02612H. 39092117 PMC11290444

[ref86] Ilic A.; Schwarz J.; Johnson C.; de Groot L. H. M.; Kaufhold S.; Lomoth R.; Warnmark K. Photoredox catalysis via consecutive ^2^LMCT- and ^3^MLCT-excitation of an Fe(III/II)-N-heterocyclic carbene complex. Chem. Sci. 2022, 13, 9165–9175. 10.1039/D2SC02122F. 36093023 PMC9383194

[ref87] Johnson C. E.; Schwarz J.; Deegbey M.; Prakash O.; Sharma K.; Huang P.; Ericsson T.; Haggstrom L.; Bendix J.; Gupta A. K.; et al. Ferrous and ferric complexes with cyclometalating N-heterocyclic carbene ligands: a case of dual emission revisited. Chem. Sci. 2023, 14, 10129–10139. 10.1039/D3SC02806B. 37772113 PMC10530338

[ref88] Wan S.; Lu W. Reversible Photoactivated Phosphorescence of Gold(I) Arylethynyl Complexes in Aerated DMSO Solutions and Gels. Angew. Chem., Int. Ed. 2017, 56, 1784–1788. 10.1002/anie.201610762. 28079953

[ref89] Wan S.; Lin J.; Su H.; Dai J.; Lu W. Photochemically deoxygenating solvents for triplet–triplet annihilation photon upconversion operating in air. Chem. Commun. 2018, 54, 3907–3910. 10.1039/C8CC00780B. 29610790

[ref90] Montalti M.; Credi A.; Prodi L.; Gandolfi M. T. Handbook of Photochemistry; CRC Press, 2006.

[ref91] Farrán A.; Deshayes K. D. Free Energy Dependence of Intermolecular Triplet Energy Transfer: Observation of the Inverted Region. J. Phys. Chem. 1996, 100, 3305–3307. 10.1021/jp9531467.

[ref92] De Kreijger S.; Glaser F.; Troian-Gautier L. From Photons to Reactions: Key Concepts in Photoredox Catalysis. Chem Catal. 2024, 4, 101100 10.1016/j.checat.2024.101110.

[ref93] Rebek J. Molekulare Erkennung mit konkaven Modellverbindungen. Angew. Chem. 1990, 102, 261–272. 10.1002/ange.19901020306.

[ref94] Whitlock B.; Whitlock H. Effect of cavity size on supramolecular stability. J. Am. Chem. Soc. 1994, 116, 2301–2311. 10.1021/ja00085a009.

[ref95] Reek J. N. H.; Priem A. H.; Engelkamp H.; Rowan A. E.; Elemans J. A. A. W.; Nolte R. J. M. Binding Features of Molecular Clips. Separation of the Effects of Hydrogen Bonding and π–π Interactions. J. Am. Chem. Soc. 1997, 119, 9956–9964. 10.1021/ja970805f.

[ref96] Wang J. W.; Huang H. H.; Wang P.; Yang G.; Kupfer S.; Huang Y.; Li Z.; Ke Z.; Ouyang G. Co-facial π–π Interaction Expedites Sensitizer-to-Catalyst Electron Transfer for High-Performance CO_2_ Photoreduction. JACS Au 2022, 2, 1359–1374. 10.1021/jacsau.2c00073. 35783182 PMC9241016

[ref97] Pfund B.; Gejsnæs-Schaad D.; Lazarevski B.; Wenger O. S. Picosecond reactions of excited radical ion super-reductants. Nat. Commun. 2024, 15, 4738 10.1038/s41467-024-49006-5. 38834625 PMC11150445

[ref98] Rosemann N. W.; Lindh L.; Bolano Losada I.; Kaufhold S.; Prakash O.; Ilic A.; Schwarz J.; Warnmark K.; Chabera P.; Yartsev A.; et al. Competing dynamics of intramolecular deactivation and bimolecular charge transfer processes in luminescent Fe(iii) N-heterocyclic carbene complexes. Chem. Sci. 2023, 14, 3569–3579. 10.1039/D2SC05357H. 37006696 PMC10056060

[ref99] Genovese D.; Cingolani M.; Rampazzo E.; Prodi L.; Zaccheroni N. Static quenching upon adduct formation: a treatment without shortcuts and approximations. Chem. Soc. Rev. 2021, 50, 8414–8427. 10.1039/D1CS00422K. 34142693

[ref100] Sinha N.; Yaltseva P.; Wenger O. S. The Nephelauxetic Effect Becomes an Important Design Factor for Photoactive First-Row Transition Metal Complexes. Angew. Chem., Int. Ed. 2023, 62, e202303864 10.1002/anie.202303864. 37057372

[ref101] Nandi A.; Manna B.; Ghosh R. Interplay of exciton-excimer dynamics in 9,10-diphenylanthracene nanoaggregates and thin films revealed by time-resolved spectroscopic studies. Phys. Chem. Chem. Phys. 2019, 21, 11193–11202. 10.1039/C9CP01124B. 31099362

[ref102] Manna B.; Nandi A.; Nath S.; Agarwal N.; Ghosh R. Comparative studies of photophysics and exciton dynamics of different diphenylanthracene (DPA) nanoaggregates. J. Photochem. Photobiol., A 2020, 400, 112700 10.1016/j.jphotochem.2020.112700.

[ref103] Trippmacher S.; Demeshko S.; Prescimone A.; Meyer F.; Wenger O. S.; Wang C. Ferromagnetically coupled chromium(III) dimer shows luminescence and sensitizes photon upconversion. Chem.—Eur. J. 2024, 30, e202400856 10.1002/chem.202400856. 38523568

[ref104] Han J.; Jiang Y.; Obolda A.; Duan P.; Li F.; Liu M. Doublet-Triplet Energy Transfer-Dominated Photon Upconversion. J. Phys. Chem. Lett. 2017, 8, 5865–5870. 10.1021/acs.jpclett.7b02677. 29144138

[ref105] Wei Y.; An K.; Xu X.; Ye Z.; Yin X.; Cao X.; Yang C. Π-Radical Photosensitizer for Highly Efficient and Stable Near-Infrared Photon Upconversion. Adv. Opt. Mater. 2023, 12, 2301134 10.1002/adom.202301134.

[ref106] Wigner E. Nachr. Akad. Wiss. Goettingen. Math Physik, Kl, IIa 1927, 375.

[ref107] Lee A. R.; Enos C. S.; Brenton A. G. Collisional excitation of CO: a study of the wigner spin rule. Int. J. Mass Spectrom. Ion Processes 1991, 104, 49–62. 10.1016/0168-1176(91)85005-7.

[ref108] Guo D.; Knight T. E.; McCusker J. K. Angular Momentum Conservation in Dipolar Energy Transfer. Science 2011, 334, 1684–1687. 10.1126/science.1211459. 22194572

[ref109] Bürgin T. H.; Glaser F.; Wenger O. S. Shedding Light on the Oxidizing Properties of Spin-Flip Excited States in a Cr^III^ Polypyridine Complex and Their Use in Photoredox Catalysis. J. Am. Chem. Soc. 2022, 144, 14181–14194. 10.1021/jacs.2c04465. 35913126 PMC9376921

[ref110] Wang C.; Li H.; Bürgin T. H.; Wenger O. S. Cage escape governs photoredox reaction rates and quantum yields. Nat. Chem. 2024, 16, 1151–1159. 10.1038/s41557-024-01482-4. 38499849 PMC11230909

[ref111] Förster T. Zwischenmolekulare Energiewanderung und Fluoreszenz. Ann. Phys. 1948, 437, 55–75. 10.1002/andp.19484370105.

[ref112] Sahoo H. Förster resonance energy transfer – A spectroscopic nanoruler: Principle and applications. J. Photochem. Photobiol., C 2011, 12, 20–30. 10.1016/j.jphotochemrev.2011.05.001.

[ref113] Gray V.; Dzebo D.; Lundin A.; Alborzpour J.; Abrahamsson M.; Albinsson B.; Moth-Poulsen K. Photophysical characterization of the 9,10-disubstituted anthracene chromophore and its applications in triplet–triplet annihilation photon upconversion. J. Mater. Chem. C 2015, 3, 11111–11121. 10.1039/C5TC02626A.

[ref114] Panigrahi S. K.; Mishra A. K. Inner filter effect in fluorescence spectroscopy: As a problem and as a solution. J. Photochem. Photobiol., C 2019, 41, 100318 10.1016/j.jphotochemrev.2019.100318.

[ref115] Olesund A.; Johnsson J.; Edhborg F.; Ghasemi S.; Moth-Poulsen K.; Albinsson B. Approaching the Spin-Statistical Limit in Visible-to-Ultraviolet Photon Upconversion. J. Am. Chem. Soc. 2022, 144, 3706–3716. 10.1021/jacs.1c13222. 35175751 PMC8895402

[ref116] Monguzzi A.; Mezyk J.; Scotognella F.; Tubino R.; Meinardi F. Upconversion-induced fluorescence in multicomponent systems: Steady-state excitation power threshold. Phys. Rev. B: Condens. Matter Mater. Phys. 2008, 78, 195112 10.1103/PhysRevB.78.195112.

[ref117] Edhborg F.; Olesund A.; Albinsson B. Best practice in determining key photophysical parameters in triplet-triplet annihilation photon upconversion. Photochem. Photobiol. Sci. 2022, 21, 1143–1158. 10.1007/s43630-022-00219-x. 35441266

[ref118] Durandin N. A.; Isokuortti J.; Efimov A.; Vuorimaa-Laukkanen E.; Tkachenko N. V.; Laaksonen T. Critical Sensitizer Quality Attributes for Efficient Triplet–Triplet Annihilation Upconversion with Low Power Density Thresholds. J. Phys. Chem. C 2019, 123, 22865–22872. 10.1021/acs.jpcc.9b08026.

[ref119] Zhou Y.; Castellano F. N.; Schmidt T. W.; Hanson K. On the Quantum Yield of Photon Upconversion via Triplet–Triplet Annihilation. ACS Energy Lett. 2020, 5, 2322–2326. 10.1021/acsenergylett.0c01150.

[ref120] Ye C.; Gray V.; Mårtensson J.; Börjesson K. Annihilation Versus Excimer Formation by the Triplet Pair in Triplet-Triplet Annihilation Photon Upconversion. J. Am. Chem. Soc. 2019, 141, 9578–9584. 10.1021/jacs.9b02302. 31131601 PMC6608582

[ref121] Hou L.; Olesund A.; Thurakkal S.; Zhang X.; Albinsson B. Efficient Visible-to-UV Photon Upconversion Systems Based on CdS Nanocrystals Modified with Triplet Energy Mediators. Adv. Funct. Mater. 2021, 31, 2106198 10.1002/adfm.202106198.

[ref122] Glaser F.; Schmitz M.; Kerzig C. Coulomb interactions for mediator-enhanced sensitized triplet-triplet annihilation upconversion in solution. Nanoscale 2023, 16, 123–137. 10.1039/D3NR05265F. 38054748

[ref123] Schmitz M.; Bertrams M.-S.; Sell A. C.; Glaser F.; Kerzig C. Efficient Energy and Electron Transfer Photocatalysis with a Coulombic Dyad. J. Am. Chem. Soc. 2024, 146, 25799–25812. 10.1021/jacs.4c08551. 39227057

[ref124] Ajekwene K. Properties and Applications of Acrylates. In Acrylate Polymers for Advanced Applications; Serrano-Aroca Á., Deb S., Eds.; IntechOpen, 2020.

[ref125] Corsaro C.; Neri G.; Santoro A.; Fazio E. Acrylate and Methacrylate Polymers’ Applications: Second Life with Inexpensive and Sustainable Recycling Approaches. Materials 2022, 15, 282 10.3390/ma15010282. PMC8746205 35009430

[ref126] Zivic N.; Bouzrati-Zerelli M.; Kermagoret A.; Dumur F.; Fouassier J. P.; Gigmes D.; Lalevée J. Photocatalysts in Polymerization Reactions. ChemCatChem 2016, 8, 1617–1631. 10.1002/cctc.201501389.

[ref127] Pan X.; Tasdelen M. A.; Laun J.; Junkers T.; Yagci Y.; Matyjaszewski K. Photomediated controlled radical polymerization. Prog. Polym. Sci. 2016, 62, 73–125. 10.1016/j.progpolymsci.2016.06.005.

[ref128] Wu C.; Corrigan N.; Lim C. H.; Liu W.; Miyake G.; Boyer C. Rational Design of Photocatalysts for Controlled Polymerization: Effect of Structures on Photocatalytic Activities. Chem. Rev. 2022, 122, 5476–5518. 10.1021/acs.chemrev.1c00409. 34982536 PMC9815102

[ref129] Serevičius T.; Komskis R.; Adomėnas P.; Adomėnienė O.; Kreiza G.; Jankauskas V.; Kazlauskas K.; Miasojedovas A. n.; Jankus V.; Monkman A.; et al. Triplet–Triplet Annihilation in 9,10-Diphenylanthracene Derivatives: The Role of Intersystem Crossing and Exciton Diffusion. J. Phys. Chem. C 2017, 121, 8515–8524. 10.1021/acs.jpcc.7b01336.

[ref130] Hu A.; Chen Y.; Guo J. J.; Yu N.; An Q.; Zuo Z. Cerium-Catalyzed Formal Cycloaddition of Cycloalkanols with Alkenes through Dual Photoexcitation. J. Am. Chem. Soc. 2018, 140, 13580–13585. 10.1021/jacs.8b08781. 30289250

[ref131] Neumeier M.; Chakraborty U.; Schaarschmidt D.; de la Pena O’Shea V.; Perez-Ruiz R.; Jacobi von Wangelin A. Combined Photoredox and Iron Catalysis for the Cyclotrimerization of Alkynes. Angew. Chem., Int. Ed. 2020, 59, 13473–13478. 10.1002/anie.202000907. PMC7496134 32190960

[ref132] Allushi A.; Jockusch S.; Yilmaz G.; Yagci Y. Photoinitiated Metal-Free Controlled/Living Radical Polymerization Using Polynuclear Aromatic Hydrocarbons. Macromolecules 2016, 49, 7785–7792. 10.1021/acs.macromol.6b01752.

[ref133] Parkatzidis K.; Rolland M.; Truong N. P.; Anastasaki A. Tailoring polymer dispersity by mixing ATRP initiators. Polym. Chem. 2021, 12, 5583–5588. 10.1039/D1PY01044A.

[ref134] Theriot J. C.; Lim C.-H.; Yang H.; Ryan M. D.; Musgrave C. B. M.; Miyake G. M. Organocatalyzed atom transfer radical polymerization driven by visible light. Science 2016, 352, 1082–1086. 10.1126/science.aaf3935. 27033549

[ref135] Rehm D.; Weller A. Kinetik und Mechanismus der Elektronübertragung bei der Fluoreszenzlöschung in Acetonitril. Ber. Bunsen-Ges. Phys. Chem. 1969, 73, 834–839. 10.1002/bbpc.19690730818.

[ref136] Rehm D.; Weller A. Kinetics of Fluorescence Quenching by Electron and H-Atom Transfer. Isr. J. Chem. 1970, 8, 259–271. 10.1002/ijch.197000029.

[ref137] Rosspeintner A.; Angulo G.; Vauthey E. Bimolecular photoinduced electron transfer beyond the diffusion limit: the Rehm-Weller experiment revisited with femtosecond time resolution. J. Am. Chem. Soc. 2014, 136, 2026–2032. 10.1021/ja4118279. 24400958

[ref138] Miyake G. M.; Theriot J. C. Perylene as an Organic Photocatalyst for the Radical Polymerization of Functionalized Vinyl Monomers through Oxidative Quenching with Alkyl Bromides and Visible Light. Macromolecules 2014, 47, 8255–8261. 10.1021/ma502044f.

[ref139] Discekici E. H.; Anastasaki A.; Read de Alaniz J.; Hawker C. J. Evolution and Future Directions of Metal-Free Atom Transfer Radical Polymerization. Macromolecules 2018, 51, 7421–7434. 10.1021/acs.macromol.8b01401.

[ref140] Corbin D. A.; Miyake G. M. Photoinduced Organocatalyzed Atom Transfer Radical Polymerization (O-ATRP): Precision Polymer Synthesis Using Organic Photoredox Catalysis. Chem. Rev. 2022, 122, 1830–1874. 10.1021/acs.chemrev.1c00603. 34842426 PMC9815475

[ref141] Bortolato T.; Simionato G.; Vayer M.; Rosso C.; Paoloni L.; Benetti E. M.; Sartorel A.; Leboeuf D.; Dell’Amico L. The Rational Design of Reducing Organophotoredox Catalysts Unlocks Proton-Coupled Electron-Transfer and Atom Transfer Radical Polymerization Mechanisms. J. Am. Chem. Soc. 2023, 145, 1835–1846. 10.1021/jacs.2c11364. 36608266 PMC9881005

[ref142] Schwarz J.; Ilic A.; Johnson C.; Lomoth R.; Wärnmark K. High turnover photocatalytic hydrogen formation with an Fe(III) N-heterocyclic carbene photosensitiser. Chem. Commun. 2022, 58, 5351–5354. 10.1039/D2CC01016J. 35373799

[ref143] Woodhouse M. D.; McCusker J. K. Mechanistic Origin of Photoredox Catalysis Involving Iron(II) Polypyridyl Chromophores. J. Am. Chem. Soc. 2020, 142, 16229–16233. 10.1021/jacs.0c08389. 32914970

[ref144] Leis W.; Arguello Cordero M. A.; Lochbrunner S.; Schubert H.; Berkefeld A. A Photoreactive Iron(II) Complex Luminophore. J. Am. Chem. Soc. 2022, 144, 1169–1173. 10.1021/jacs.1c13083. 35025493

[ref145] Tyson D. S.; Castellano F. N.; Castellano F. N. Intramolecular Singlet and Triplet Energy Transfer in a Ruthenium(II) Diimine Complex Containing Multiple Pyrenyl Chromophores. J. Phys. Chem. A 1999, 103, 10955–10960. 10.1021/jp992983w.

[ref146] Tyson D. S.; Henbest K. B.; Bialecki J.; Castellano F. N. Excited State Processes in Ruthenium(II)/Pyrenyl Complexes Displaying Extended Lifetimes. J. Phys. Chem. A 2001, 105, 8154–8161. 10.1021/jp011770f.

[ref147] Ragazzon G.; Verwilst P.; Denisov S. A.; Credi A.; Jonusauskas G.; McClenaghan N. D. Ruthenium(II) complexes based on tridentate polypyridine ligands that feature long-lived room-temperature luminescence. Chem. Commun. 2013, 49, 9110–9112. 10.1039/c3cc45387a. 23989237

[ref148] Reichardt C.; Schneider K. R. A.; Sainuddin T.; Wächtler M.; McFarland S. A.; Dietzek B. Excited State Dynamics of a Photobiologically Active Ru(II) Dyad Are Altered in Biologically Relevant Environments. J. Phys. Chem. A 2017, 121, 5635–5644. 10.1021/acs.jpca.7b04670. 28678497

[ref149] Isakov D.; Giereth R.; Nauroozi D.; Tschierlei S.; Rau S. Two Emissive Long-Lived Excited States of an Imidazole-Functionalized Ruthenium Dipyridophenazine Complex. Inorg. Chem. 2019, 58, 12646–12653. 10.1021/acs.inorgchem.9b01372. 31532651

[ref150] Sell A. C.; Wetzel J. C.; Schmitz M.; Maijenburg A. W.; Woltersdorf G.; Naumann R.; Kerzig C. Water-soluble ruthenium complex-pyrene dyads with extended triplet lifetimes for efficient energy transfer applications. Dalton Trans. 2022, 51, 10799–10808. 10.1039/D2DT01157C. 35788236

[ref151] Yarnell J. E.; Wells K. A.; Palmer J. R.; Breaux J. M.; Castellano F. N. Excited-State Triplet Equilibria in a Series of Re(I)-Naphthalimide Bichromophores. J. Phys. Chem. B 2019, 123, 7611–7627. 10.1021/acs.jpcb.9b05688. 31405284

[ref152] Yarnell J. E.; Deaton J. C.; McCusker C. E.; Castellano F. N. Bidirectional ″ping-pong″ energy transfer and 3000-fold lifetime enhancement in a Re(I) charge transfer complex. Inorg. Chem. 2011, 50, 7820–7830. 10.1021/ic200974h. 21761837

[ref153] Sasaki Y.; Amemori S.; Yanai N.; Kimizuka N. Singlet-to-Triplet Absorption for Near-Infrared-to-Visible Photon Upconversion. Bull. Chem. Soc. Jpn. 2021, 94, 1760–1768. 10.1246/bcsj.20210114.

[ref154] Sasaki Y.; Yanai N.; Kimizuka N. Osmium Complex-Chromophore Conjugates with Both Singlet-to-Triplet Absorption and Long Triplet Lifetime through Tuning of the Heavy-Atom Effect. Inorg. Chem. 2022, 61, 5982–5990. 10.1021/acs.inorgchem.1c03129. 35080875

[ref155] Kazama A.; Imai Y.; Okayasu Y.; Yamada Y.; Yuasa J.; Aoki S. Design and Synthesis of Cyclometalated Iridium(III) Complexes-Chromophore Hybrids that Exhibit Long-Emission Lifetimes Based on a Reversible Electronic Energy Transfer Mechanism. Inorg. Chem. 2020, 59, 6905–6922. 10.1021/acs.inorgchem.0c00363. 32352765

[ref156] Wang P.; Koo Y. H.; Kim W.; Yang W.; Cui X.; Ji W.; Zhao J.; Kim D. Broadband Visible Light Harvesting N̂N Pt(II) Bisacetylide Complex with Bodipy and Naphthalene Diimide Ligands: Förster Resonance Energy Transfer and Intersystem Crossing. J. Phys. Chem. C 2017, 121, 11117–11128. 10.1021/acs.jpcc.7b02188.

[ref157] Kim D.; Rosko M. C.; Castellano F. N.; Gray T. G.; Teets T. S. Long Excited-State Lifetimes in Three-Coordinate Copper(I) Complexes via Triplet–Triplet Energy Transfer to Pyrene-Decorated Isocyanides. J. Am. Chem. Soc. 2024, 146, 19193–19204. 10.1021/jacs.4c04288. 38956456

[ref158] Doettinger F.; Yang Y.; Karnahl M.; Tschierlei S. Bichromophoric Photosensitizers: How and Where to Attach Pyrene Moieties to Phenanthroline to Generate Copper(I) Complexes. Inorg. Chem. 2023, 62, 8166–8178. 10.1021/acs.inorgchem.3c00482. 37200533 PMC10230506

[ref159] Yang Y.; Doettinger F.; Kleeberg C.; Frey W.; Karnahl M.; Tschierlei S. How the Way a Naphthalimide Unit is Implemented Affects the Photophysical and -catalytic Properties of Cu(I) Photosensitizers. Front. Chem. 2022, 10, 936863 10.3389/fchem.2022.936863. 35783217 PMC9247301

