# Supplementary material for: Luminescent Fe(III) Complex Sensitizes Aerobic Photon Upconversion and Initiates Photocatalytic Radical Polymerization
Source: J Am Chem Soc. 2024 Dec 10;146(51):35390–401. doi: 10.1021/jacs.4c14248 (PMC11673105; doi:10.1021/jacs.4c14248)
Supplement: Supplementary file 1 — ja4c14248_si_001.pdf [file ja4c14248_si_001.pdf]

Supporting Information

Luminescent Fe(III) complex sensitizes aerobic photon upconversion and initiates photocatalytic radical polymerization

Pengyue Jin,[a] Xinhuan Xu,[b] Yongli Yan,[b] Heinrich Hammecke,[a] Cui Wang [a]*

[a] Department of Biology and Chemistry, Osnabrück University, Barbarastraße 7, 49076 Osnabrück, Germany

[b] Key Laboratory of Photochemistry, Institute of Chemistry, Chinese Academy of Sciences, Beijing 100190, China.

Contents

1. Materials and methods 4
2. Synthesis and characterization 7
   2.1 Synthesis of $PhB(MeIm)_3(OTf)_2$ (L-OTf) 7
   2.2 Synthesis of $PhB(MeIm)_3(PF_6)_2$ (L-PF_6) 8
   2.3 Synthesis of [Fe(phtmeimb)$_2$]PF_6 8
3. Density functional theory calculations 9
4. Optical characterization of the sensitizer and the annihilators 11
   4.1 Anthracene (An) 11
   4.2 9-Phenylanthracene (PhAn) 11
   4.3 9,10-Diphenylanthracene (DPA) 12
   4.4 [Fe(phtmeimb)$_2$]PF_6 12
5. Stern-Volmer studies 14
   5.1 Fe(III) + An 14
   5.2 Fe(III) + PhAn 15
   5.3 Fe(III) + DPA 16
   5.4 Spin states in doublet-triplet energy transfer 18
6. NMR titration for the Fe(III)/DPA pair 19
7. Transient absorption (TA) measurements 26
   7.1 fs - TA spectroscopy of the Fe(III)/DPA pair 26
   7.2 ns - TA spectroscopy of the Fe(III)/An pair 27
   7.3 ns - TA spectroscopy of the Fe(III)/PhAn pair 28
   7.4 ns - TA spectroscopy of the Fe(III)/DPA pair 30
8. Photon upconversion studies 33
   8.1 Excitation power density dependence of [Fe(phtmeimb)$_2$]PF_6 33
   8.2 Control experiments 34
   8.3 Fe(III) + An 35
   8.4 Fe(III) + PhAn 38
   8.5 Fe(III) + DPA 41

9. sTTA-UC with mediator 45
9.1 Fe(III) + PhAn with An as the mediator 45
9.2 Fe(III) + DPA with An as the mediator 47
9.3 Fe(III) + DPA with PhAn as the mediator 49
10. Photostability 51
10.1 Fe(III) + DPA 51
10.2 Fe(III) + DPA with An as the mediator 52
11. Photocatalytic polymerizations 52
11.1 Reaction mechanism 52
11.2 Trimethylolpropane triacrylate (TMPTA) 57
11.3 Polyethylene glycol diacrylate (PEGDA) 58
12. NMR data 60
13. References 63

1. Materials and methods

Dry Toluene (99.8%, Fisher Chemical), dichloromethane (99.8%, Fisher Chemical), dry acetone (99.8%, Thermo Fisher Scientific), tetrahydrofuran (99.9%, Thermo Fisher Scientific), dry acetonitrile (99.9%, Thermo Fisher Scientific), dry dimethyl sulfoxide (DMSO, 99.7%), deuterated acetonitrile (CD_3CN, 99.8%, Eurisotope), and deuterated dimethyl sulfoxide (DMSO-d_6, 99.8% Apollo Scientific) were used as commercially received.

The anthracene annihilators were used as commercially received: anthracene (An, 99%, Thermo Fisher Scientific), 9-phenylanthracene (PhAn, 98%, BLDpharm), and 9,10-diphenylanthrace (DPA, 99.95%, Thermo Fisher Scientific).

All chemicals for synthesis and optical spectroscopy with high purity were used as received from commercial suppliers (Acros Organics, Alfa Aesar, Fischer Scientific and Sigma-Aldrich), unless indicated otherwise.

The solutions for UV-vis absorption, luminescence, and transient absorption measurements with ns-pulsed lasers were prepared in dry solvents under ambient aerobic conditions at room temperature and measured in long-neck quartz cuvettes (10 x 10 mm). For fs-transient absorption studies, the solutions were measured in 1 x 10 mm quartz cuvettes. For deaerated solutions, the samples were purged with argon for 30 minutes.

NMR characterization of the reaction products was performed with a Bruker Avance AMX-500 operating at 500 MHz proton frequencies in deuterated solvents (Deutero). ^{13}C-NMR were performed on the same instrument operating at 126 MHz. All chemical shifts are reported in ppm-values and were referenced to their respective residual proton NMR signal. All coupling constants *J* are given in Hertz (Hz), and the following abbreviations are used to describe their coupling patterns: s (singlet), d (doublet), t (triplet), q (quartet), m (multiplet) and combinations of these abbreviations.

NMR titration and determination of the binding constant. For typical ^{1}H NMR titration experiments, a 0.6 mL solution of the host ($[Fe(phtmeimb)_2]PF_6$, 1.0×10^{-3} M) in DMSO-d_6 was titrated with another solution containing the same concentration of host ($[Fe(phtmeimb)_2]PF_6$, 1.0×10^{-3} M) and the concentrated guest molecules (DPA, 1×10^{-2} M) in DMSO-d_6 via a 0 ~ 50 microliter injector. Upon each addition, the solution was manually stirred for 1 min before acquiring the spectrum, which allowed equilibrium to be reached between the host and guest. Binding isotherms for the NMR titration were calculated from a global proton shift analysis using the software BINDFIT to obtain a binding isotherm.[1] The fitting is based on the proton shift differences ($\delta - \delta_0$) between the host-only data and the data at varying guest concentrations, following literature-known models.[2] The proton signals used

in the fitting are assigned to different protons in $[Fe(phtmeimb)_2]PF_6$ and one proton from DPA was considered for the analysis.

UV-vis steady-state absorption spectra were recorded on a calibrated Varian Carry 6000 spectrophotometer using 10 x 10 mm cuvettes. Particularly, absorption spectra for the Φ_{UC} determination were collected with low measuring speed (5 nm / 1 s) to suppress the influence from the noises.

Steady-state and time-resolved luminescence measurements: Luminescence spectra and decays were measured on a Horiba Jobin-Yvon Fluorolog 6000 spectrometer, which is equipped with a xenon lamp and a flash lamp as the light sources, as well as a detector using time correlated single photon counting (TCSPC) technique and a photomultiplier tube (PMT) detector. For luminophores with relatively short luminescence lifetime (< 10 μs), external pulsed LEDs (390 nm or 455 nm, pulse width < 1.3 ns) were used as the light source.

Upconversion luminescence measurements: For measuring the upconversion (UC) luminescence spectra, a tunable 532 nm cw-laser (200 mW) (Lamda Beam, RGB Lasersystems) was used as the excitation light source. The 532 nm laser is focused on the sample position, and the round-shaped laser spot diameters were determined by the manufacture to be 0.42 mm at the sample position. For the upconversion luminescence lifetime measurements, a high throughput TCSPC controller (DeltaHub from Horiba Scientific) was used to trigger the cw-lasers with a pulse width of 250 μs.

Φ_{PL} determination: For the determination of the photoluminescence quantum yield Φ_{PL} for the $[Fe(phtmeimb)_2]PF_6$ in DMSO, $[Ru(bpy)_3]Cl_2$ in deaerated water was used as the reference sample. Φ_{PL} was calculated according to Eq. S1.

$$\Phi_{Fe} = \frac{A_{Ru}}{A_{Fe}} \cdot \frac{I_{Fe}}{I_{Ru}} \cdot \left(\frac{\eta_{DMSO}}{\eta_{water}}\right)^2 \cdot \Phi_{Ru} \qquad \text{(Eq. S1)}$$

In Eq. S1, A_{Ru} and A_{Fe} stand for the absorbances of the $[Ru(bpy)_3]Cl_2$ reference solution and the $[Fe(phtmeimb)_2]PF_6$ sample at the excitation wavelength, respectively. I_{Fe} and I_{Ru} represent the integrated luminescence intensities of the $[Fe(phtmeimb)_2]PF_6$ and the $[Ru(bpy)_3]Cl_2$ emission, respectively. η represents the refractive index of the applied solvents at room temperature. Φ_{Ru}, the luminescence quantum yield of $[Ru(bpy)_3]Cl_2$ in deaerated water at room temperature, equals 6.3%.[3] Both $[Ru(bpy)_3]Cl_2$ reference and $[Fe(phtmeimb)_2]PF_6$ samples were independently prepared and measured twice on different days.

Φ_{UC} determination: For the determination of upconversion luminescence quantum yields Φ_{UC} in DMSO, the $[Fe(phtmeimb)_2]PF_6$ (40 μM) in aerated DMSO was used as the reference sample. During the measurement, a 495 nm long pass filter was placed between the laser

and the sample to make the light clean and avoid direct excitation of annihilator. Φ_{UC} of the UC samples containing 40 μM $[Fe(phtmeimb)_2]PF_6$ and anthracene-based annihilators, including An, PhAn, and DPA, were determined according to Eq. S2.[4]

$$\Phi_{UC} = \frac{A_{Fe}}{A_{UC}} \cdot \frac{I_{UC}}{I_{Fe}} \cdot \Phi_{Fe} \qquad \text{(Eq. S2)}$$

In Eq. S2, A_{Fe} and A_{UC} stand for the absorbances of the reference solution and the UC sample at the excitation wavelength, respectively. I_{UC} and I_{Fe} represent the integrated intensities of the UC luminescence intensity and the reference emission, respectively. Φ_{Fe}, which equals the luminescence quantum yield of $[Fe(phtmeimb)_2]PF_6$ in aerated DMSO at room temperature, is determined to 1.82% (section 4.4). Both reference and UC samples were independently prepared and measured twice on different days.

Determination of the quenching rates: The apparent bimolecular quenching rate constant (k_q) was derived from the Stern-Volmer studies based on the steady-state luminescence of the photoexcited $[Fe(phtmeimb)_2]PF_6$ in the absence and in the presence of the anthracenes quenchers of various concentrations, as shown in Eq. S3.[5]

$$\frac{\boldsymbol{I_0}}{\boldsymbol{I}} = \mathbf{1} + \boldsymbol{K_{SV}} \cdot [\mathbf{Q}] = \mathbf{1} + \boldsymbol{k_q} \cdot \boldsymbol{\tau_0} \cdot [\mathbf{Q}] \quad \text{(Eq. S3)}$$

in Eq. S3, I_0 and I are the integrals of the luminescence intensity of $[Fe(phtmeimb)_2]PF_6$ in the absence and presence of the anthracene annihilators, K_{SV} is the Stern-Volmer constant, τ_0 is the lifetime of the excited state of $[Fe(phtmeimb)_2]PF_6$ without anthracene annihilator, and [Q] is the concentration of the anthracene annihilators.

UV-vis ns-transient absorption spectra and decay kinetics were measured on a LP920-KS apparatus from Edinburgh Instruments. For these experiments, a frequency-doubled pulsed Nd:YAG laser (Quantel Q-smart 450, ca. 10 ns pulse width) with a beam expander (GBE02-A from Thorlabs) in the beam path was used for excitation at 532 nm (pulse energy of ~12 mJ). Transient absorption spectra were recorded with an iCCD camera from Andor, and the decay kinetics at individual detection wavelengths were measured with a photomultiplier tube (PMT).

UV-vis fs-transient absorption spectra and decay kinetics were measured using a commercial HELIOS ultrafast spectroscopy system. Pulses at 800 nm, generated by a Coherent Astrella regenerative amplifier (25 fs, 1 kHz, 4 mJ), were used to pump an optical parametric amplifier (TOPAS.C, Light Conversion), producing excitation pulses at 532 nm. A mechanical chopper modulated the pump beam at a frequency of 500 Hz. For the probe beam, a small portion of the 800 nm output was directed to a sapphire crystal in the HELIOS system to generate a time-delayed white light continuum, spanning from 400 nm to 790 nm.

The samples were positioned at the overlap of the pump and probe beams. All samples were in solution and measured using 1 mm quartz cuvettes. Taking into account the instrument response function, the system achieved a temporal resolution of approximately 125 fs. All experiments were conducted at room temperature.

Density Functional Theory (DFT) calculations: DFT calculations have been performed using the Gaussian16 program, applying default procedures, integration grids, algorithms and parameters.[6] The geometries of the anthracene derivatives were optimized at the DFT/B3LYP/6-311G(d) level of theory.[7] The molecular frontier orbital was generated by Gaussian program and visualized by Gauss View 6.[8]

2. Synthesis and characterization

2.1 Synthesis of $PhB(MeIm)_3(OTf)_2$ (L-OTf)

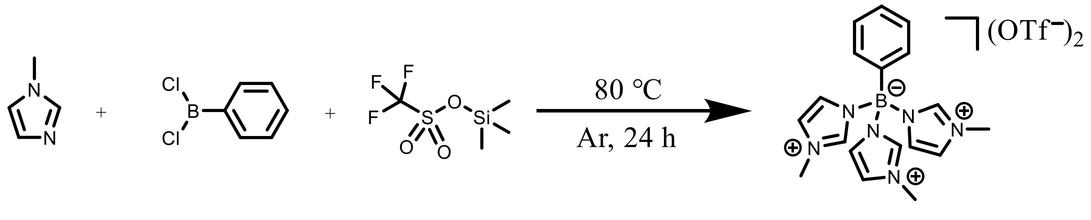

The $PhB(MeIm)_3(OTf)_2$ (L-OTf) was synthesized according to previously reported procedure.[9] 1-Methylimidazole (1.61 g, 19.7 mmol) was added dropwise to $PhBCl_2$ (1.00 g, 6.30 mmol) in toluene (15 mL) in a glovebox. After 15 min of stirring, a solution of trimethylsilyl trifluoromethanesulfonate (TMSOTf) (3.20 g, 14.5 mmol) in toluene (20 mL) was added to the solution. The reaction mixture was taken out of the glovebox and was heated at 80°C for 1 day and cooled to room temperature. An off-white precipitate was obtained by filtration under reduced pressure and extracted into dichloromethane and left to crystallize overnight at -20 °C. The white crystalline product was filtered, washed with cold dichloromethane, and dried in the oven for 2 h (3.25 g, 87.3%). The obtained ^{1}H and ^{13}C NMR signals are in agreement with the literature.[9]

^{1}H NMR (500 MHz, DMSO-d_6) δ 8.56 (s, 3H), 7.81 (t, J = 1.7 Hz, 3H), 7.49 – 7.37 (m, 6H), 7.17 – 7.08 (m, 2H), 3.85 (s, 9H).

^{13}C NMR (126 MHz, DMSO-d_6) δ 139.98, 133.16, 129.49, 128.89, 125.05, 124.42, 35.96.

2.2 Synthesis of PhB(MeIm)3(PF6)2 (L-PF6)

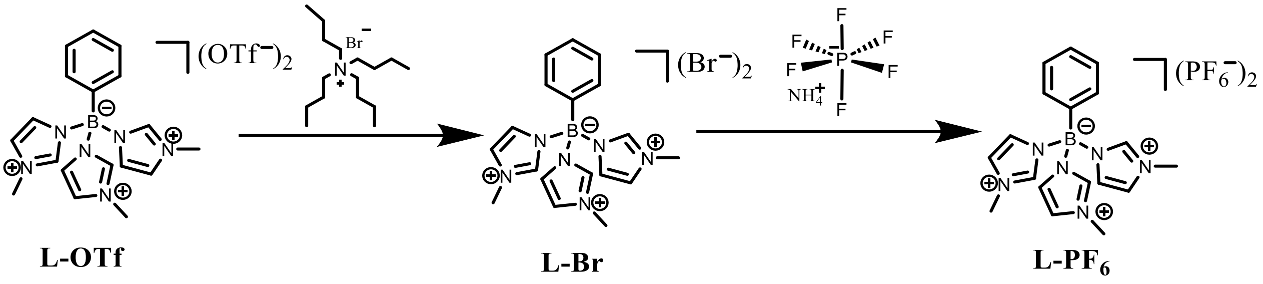

The PhB(MeIm)3(PF6)2 (L-PF6) was synthesized according to previously reported procedure.[10] L-OTf (0.790 g, 1.25 mmol) was dissolved in dry acetone (10 mL) and precipitated with tetra-n-butylammonium bromide (0.822 g, 2.55 mmol) for 10 minutes. The white precipitation was filtered under reduced pressure and washed with 30 mL acetone. The resulting bromide salt was dissolved in distilled water (10 mL) and precipitated with ammonium hexafluorophosphate (0.416 g, 2.55 mmol). The resulting precipitate was collected by filtration under reduced pressure, washed with distilled water and dried under vacuum to obtain a white solid (0.661 g, 84.7%). The obtained ^{1}H and ^{13}C NMR signals are in agreement with the literature.[10]

^{1}H NMR (500 MHz, Acetonitrile-d_3) δ 8.01 (s, 3H), 7.53 – 7.37 (m, 6H), 7.22 – 7.08 (m, 5H), 3.82 (d, J = 2.4 Hz, 9H).

^{13}C NMR (126 MHz, Acetonitrile-d_3) δ 140.62, 134.31, 130.89, 130.08, 126.12, 125.40, 37.00.

2.3 Synthesis of [Fe(phtmeimb)2]PF6

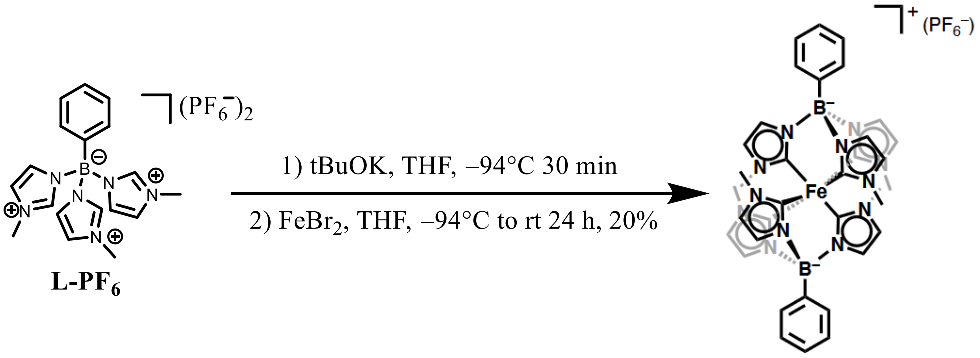

[Fe(phtmeimb)2]PF6 was synthesized by adapting the previously reported procedure.[10] L-PF6 (0.624 g, 1.00 mmol) was dried under low pressure at 80 °C overnight in a Schlenk tube connected to the house vacuum line. Dry THF (20 mL) was charged under Ar. The reaction mixture was cooled to - 94 °C (acetone / liquid N_2) under Argon and tBuOK (3.5 mL, 3.50mmol, 1 M in THF) was added dropwise. The resulting reaction mixture was stirred for 30 min at - 94 °C. The cooling bath was removed and a solution of anhydrous $FeBr_2$ (0.108 g, 0.500

mmol) in degassed dry THF (10 mL) was added by syringe to the Schlenk tube containing the in situ generated tris(imidazolylidene) ligand solution. The reaction mixture was placed to reach room temperature and stirred in the dark under argon for 24 h. The mixture was filtered over celite using glass sinter (porosity #4) and to obtain a dark red solution solvent. The solvent was removed under reduced pressure. The residue was purified by neutral aluminum oxide chromatography (Toluene : MeCN = 1:1) to afford rose-red solid (0.0867 g, 20.1%). The obtained ^{1}H and ^{13}C NMR signals are in agreement with the literature.[10]

^{1}H NMR (500 MHz, Acetonitrile-d_3) δ 14.39 (s, 4H), 10.25 (t, J = 6.5 Hz, 4H), 9.63 (t, J = 7.5 Hz, 2H), 5.06 (s, 18H), 1.27 (s, 6H), -11.72 (s, 6H).

^{13}C NMR (126 MHz, Acetonitrile-d_3) δ 161.83, 139.13, 131.44, 130.83, 58.40, 12.74, -27.43.

3. Density functional theory calculations

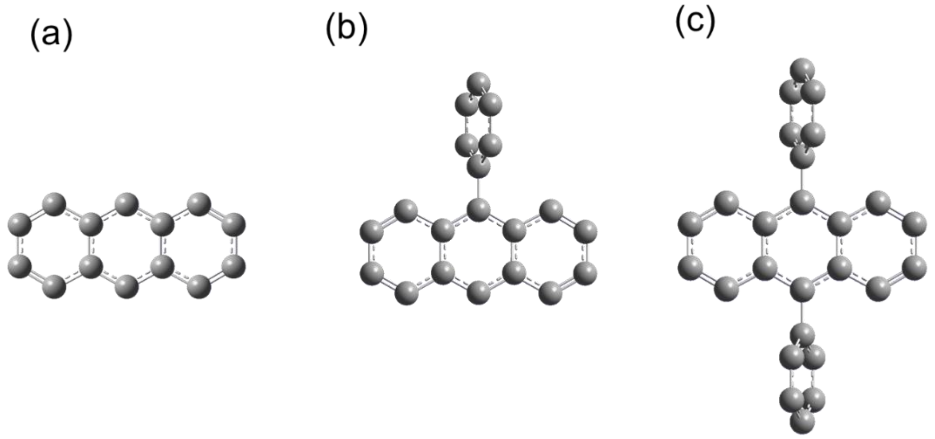

Figure S1. Geometry of the An (a), PhAn (b), and DPA (c) optimized at the DFT/B3LYP/6-311G(d) level at their optimized ground states in the gas phase. The H atoms in these structures are omitted for clarity.

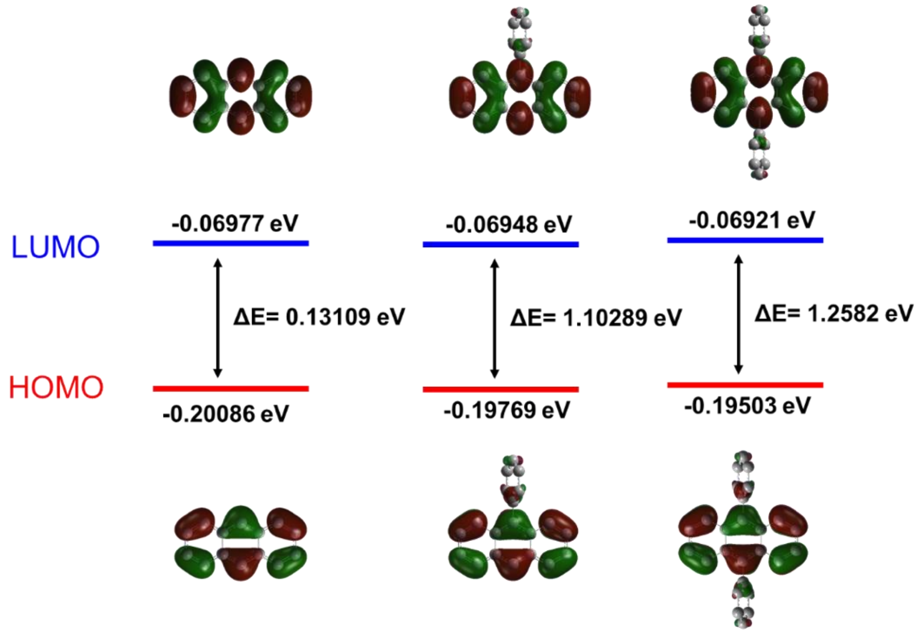

Figure S2. The energy levels of the HOMO (highest occupied molecular orbital) and LUMO (lowest unoccupied molecular orbital) and energy gaps for annihilators at the DFT/B3LYP/6-311G(d) level at their optimized ground states in the gas phase. The H atoms in these structures are omitted for clarity.

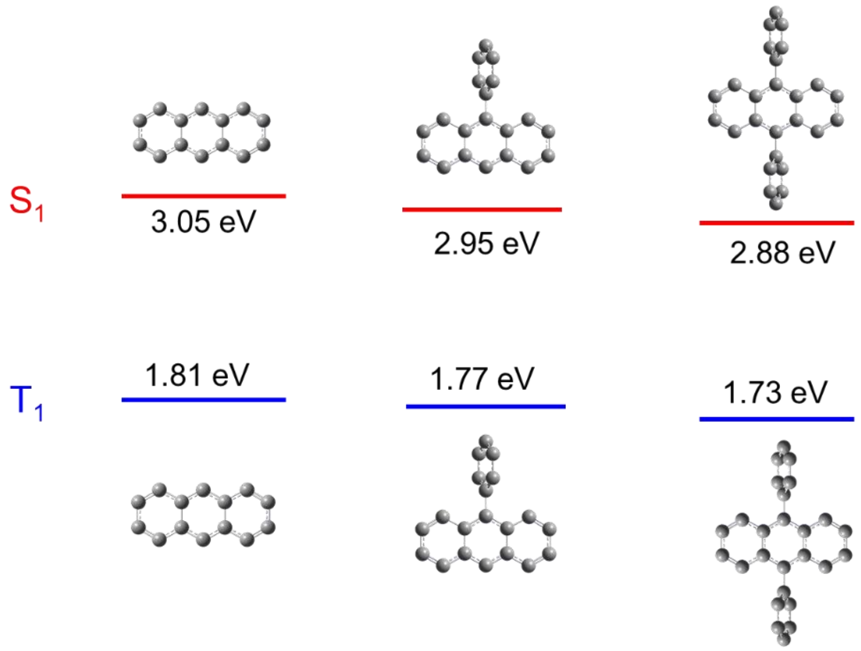

Figure S3. Geometries of the annihilators An (left), PhAn (middle), and DPA in the energetically lowest singlet state (S_1) and triplet state (T_1) at the DFT/B3LYP/6-311G(d) level in the gas phase. The H atoms in these structures are omitted for clarity.

4. Optical characterization of the sensitizer and the annihilators

4.1 Anthracene (An)

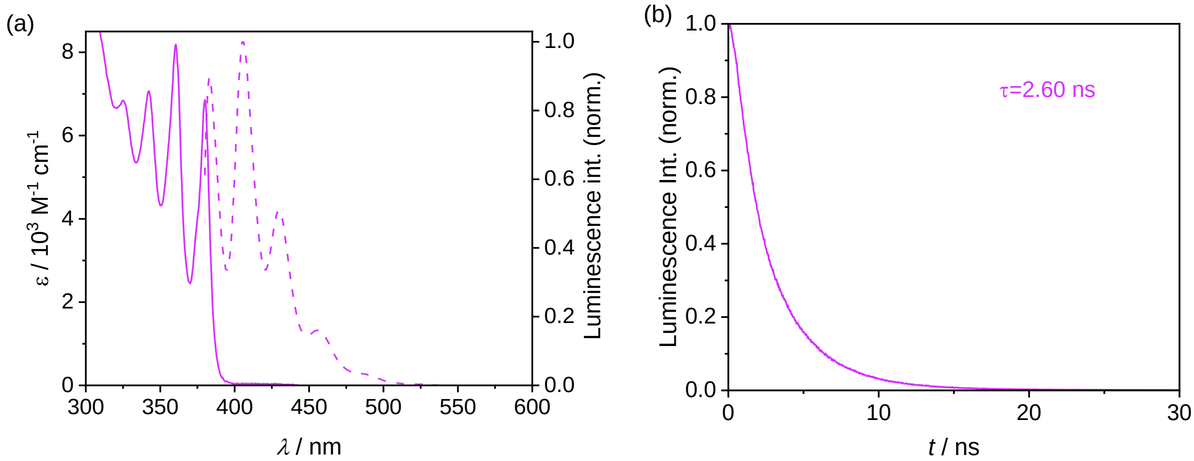

Figure S4. a) UV-vis absorption spectrum (solid traces) and normalized fluorescence spectrum (dash traces) of An (10 μM) in aerated DMSO at 20°C, along with b) the normalized luminescence decay of the sample from a) recorded at 407 nm. Excitation occurred with a pulsed LED at 390 nm. Mono-exponential fit of the fluorescence decay gives a lifetime of 2.60 ns of An in aerated DMSO at 20°C.

4.2 9-Phenylanthracene (PhAn)

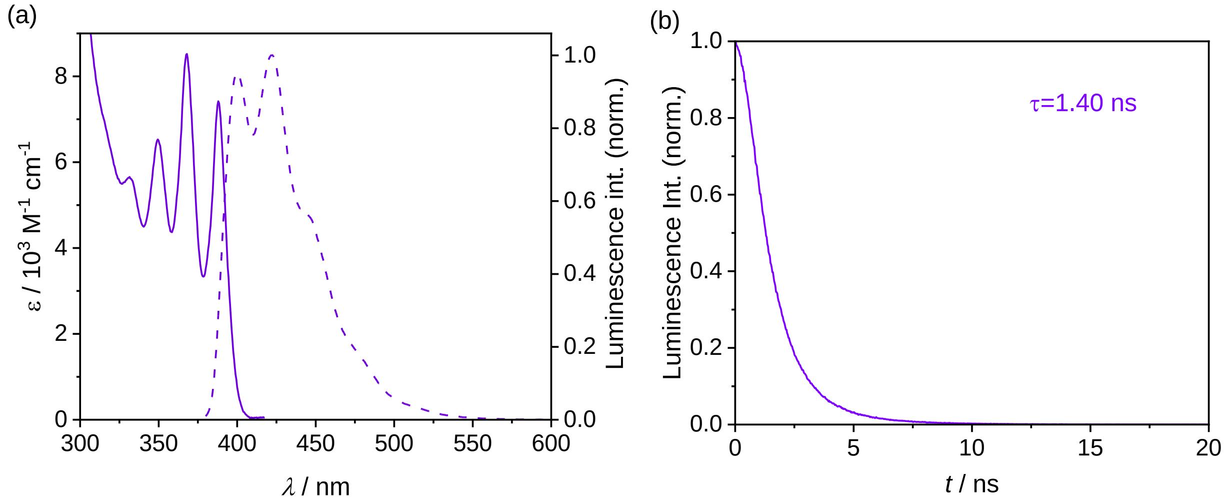

Figure S5. a) UV-vis absorption spectrum (solid traces) and normalized fluorescence spectrum (dash traces) of PhAn (10 μM) in aerated DMSO at 20°C, along with b) the normalized luminescence decay of the sample from a) recorded at 420 nm. Excitation occurred with a pulsed LED at 390 nm. Mono-exponential fit of the fluorescence decay gives a lifetime of 1.40 ns of PhAn in aerated DMSO at 20°C.

4.3 9,10-Diphenylanthracene (DPA)

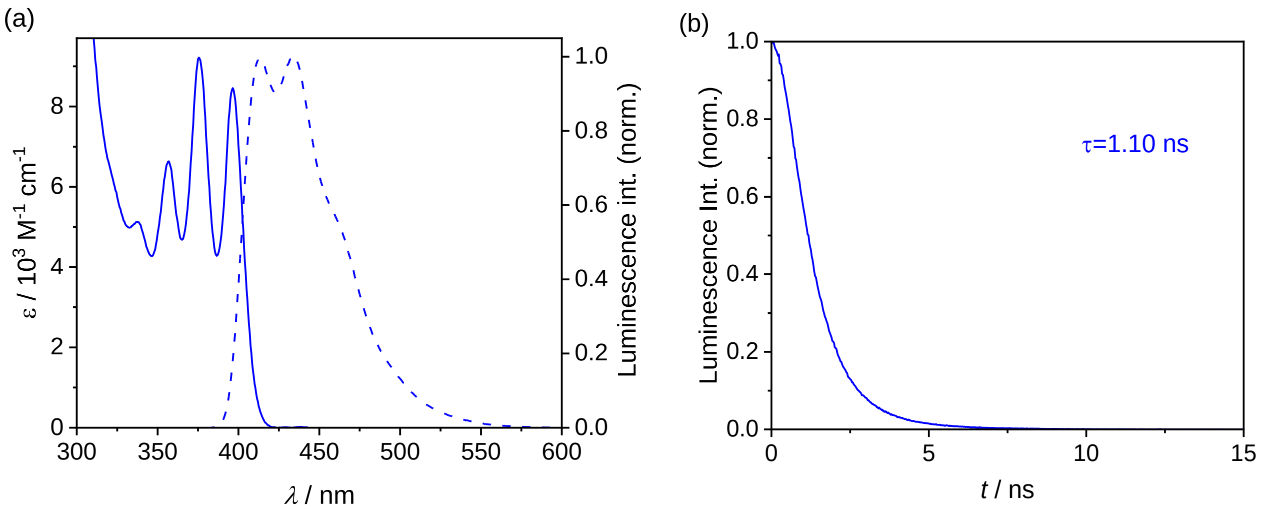

Figure S6. a) UV-vis absorption spectrum (solid traces) and normalized fluorescence spectrum (dash traces) of DPA (10 μM) in aerated DMSO at 20°C, along with b) the normalized luminescence decay of the sample from a) recorded at 430 nm. Excitation occurred with a pulsed LED at 390 nm. Mono-exponential fit of the fluorescence decay gives a lifetime of 1.10 ns of DPA in aerated DMSO at 20°C.

4.4 [Fe(phtmeimb)$_2$]PF$_6$

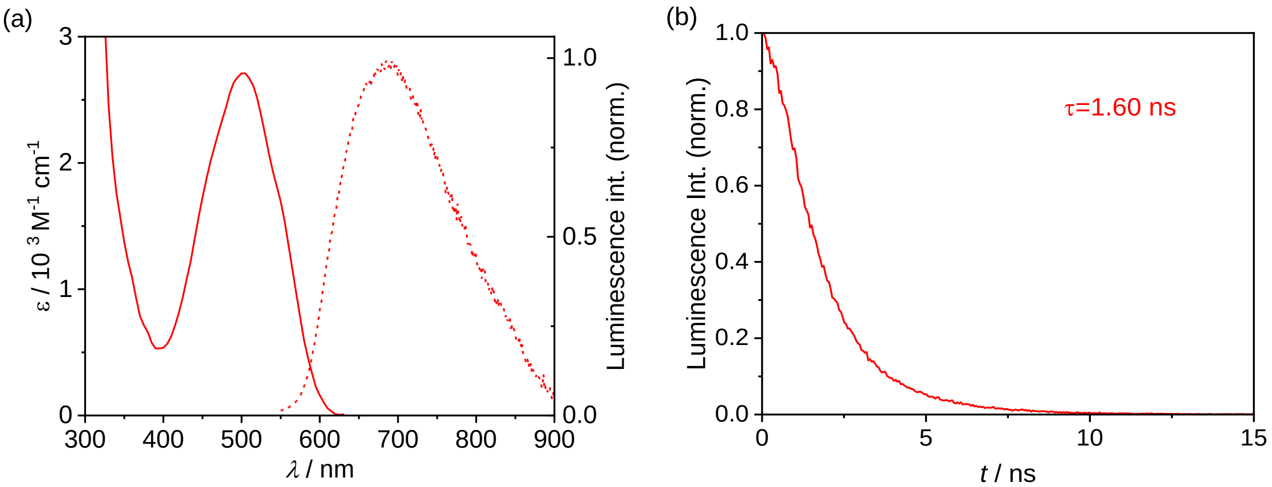

Figure S7. a) UV-vis absorption (solid traces) and normalized luminescence spectra (dotted traces, excitation at 532 nm) of [Fe(phtmeimb)$_2$]PF$_6$ (40 μM) in aerated DMSO at 20°C, along with b) normalized luminescence decays of the samples from a) recorded at 670 nm in aerated DMSO, excitation occurred with a 455 nm pulsed LED. The luminescence decay was fitted mono-exponentially, giving 1.60 ns in aerated DMSO at 20°C.

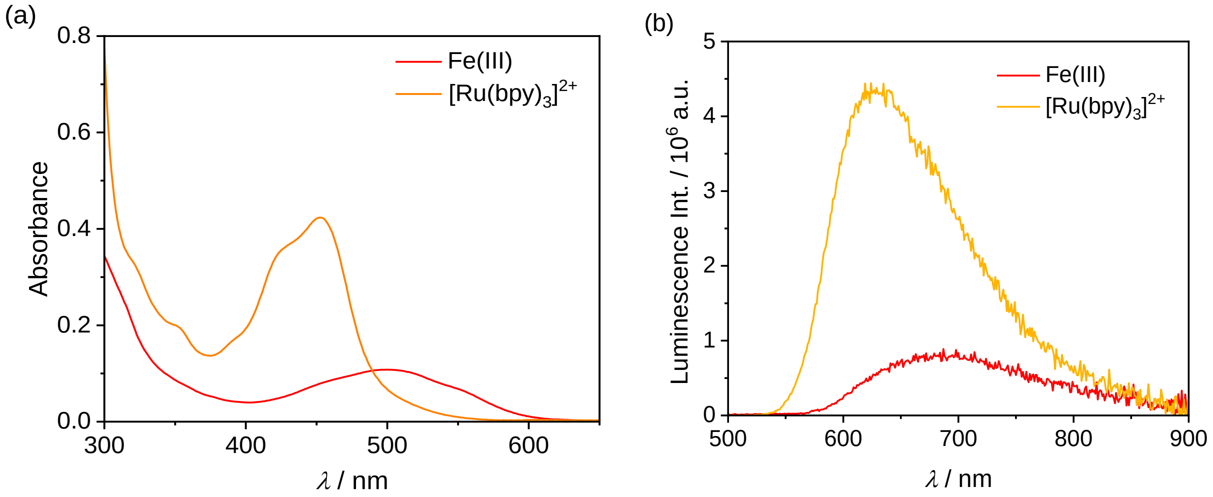

Figure S8. Determination of the photoluminescence quantum yield (Φ_{PL}) of $[Fe(phtmeimb)_2]PF_6$. a) UV-vis absorption and b) luminescence spectrum (excitation at 490 nm with a xenon lamp) of $[Fe(phtmeimb)_2]PF_6$ (40 μM) in aerated DMSO (red traces) and $[Ru(bpy)_3]Cl_2$ (40 μM) in deaerated water (orange traces) as the reference at 20°C. Φ_{PL} of the Fe(III) complex was determined according to Eq. S1 to 1.82% in aerated DMSO at 20°C.

5. Stern-Volmer studies

5.1 Fe(III) + An

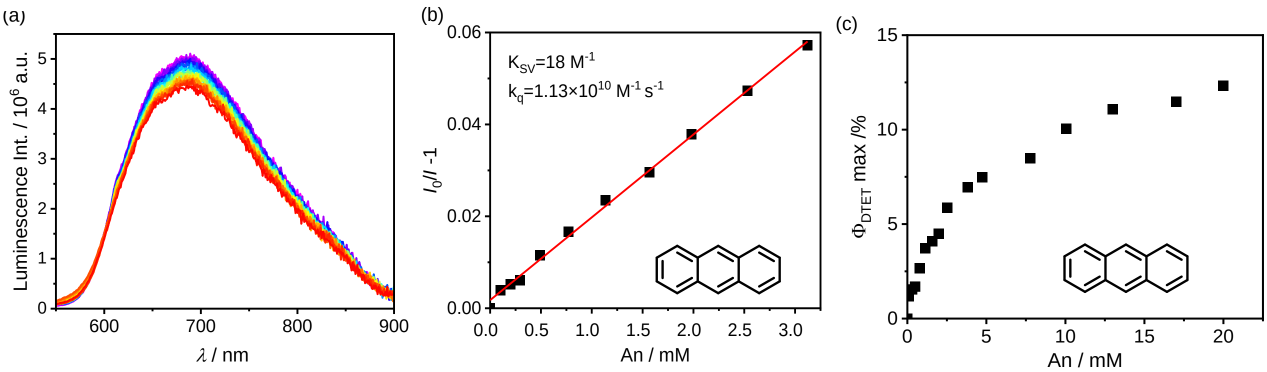

Figure S9. Luminescence intensity-based Stern-Volmer quenching of $[Fe(phtmeimb)_2]PF_6$ by An. a) Luminescence spectral changes of the Fe(III) complex (40 μM) upon titration with An (0 – 20 mM) in aerated DMSO at 20°C. b) Stern-Volmer plot in the initial linear regime and c) Φ_{DTET} derived from the quenched luminescence intensity from a) plotted as a function of the An concentration; linear fitting of the data in b) gives the Stern-Volmer constant K_{SV}, and the initial quenching rate constant k_q ($k_q = K_{SV} / \tau_0$, τ_0 = 1.60 ns from the lifetime obtained in Figure S7b). $\Phi_{DTET} = 1 - I/I_0$, I_0 and I are the luminescence intensity in the absence and presence of An in a), giving a DTET efficiency of 12.33% for the $[Fe(phtmeimb)_2]PF_6$ (40 μM)/An(20 mM) pair.

5.2 Fe(III) + PhAn

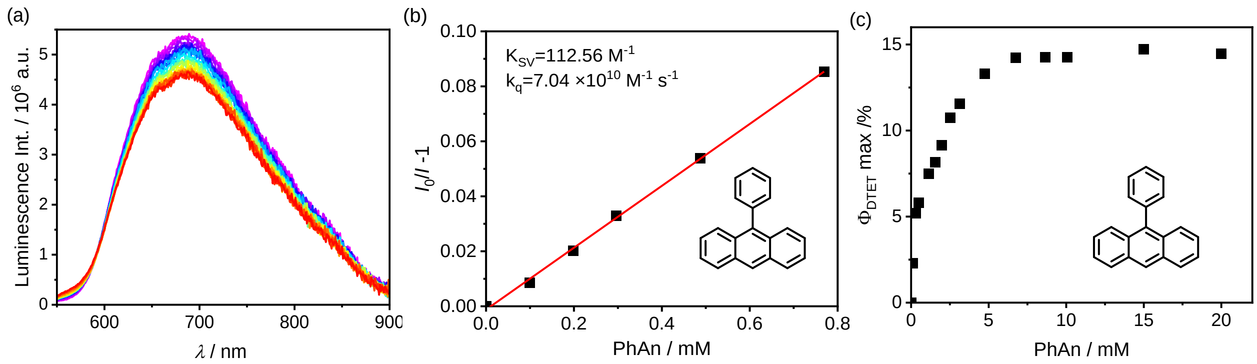

Figure S10. Luminescence intensity-based Stern-Volmer quenching of $[Fe(phtmeimb)_2]PF_6$ by PhAn. a) Luminescence spectral changes of the Fe(III) complex (40 μM) upon titration with PhAn (0 – 20 mM) in aerated DMSO at 20°C, b) Stern-Volmer plot in the initial linear regime and c) Φ_{DTET} derived from the quenched luminescence intensity from a), plotted as a function of the PhAn concentration; linear fitting of the data in b) gives the Stern-Volmer constant K_{SV}, and the initial quenching rate constant k_q ($k_q = K_{SV} / \tau_0$, τ_0 = 1.60 ns from the lifetime obtained in Figure S7b). $\Phi_{DTET} = 1 - I/I_0$, I_0 and I are the luminescence intensity in the absence and presence of PhAn in a), giving a DTET efficiency of 14.47% for the $[Fe(phtmeimb)_2]PF_6$ (40 μM)/PhAn(20 mM) pair.

5.3 Fe(III) + DPA

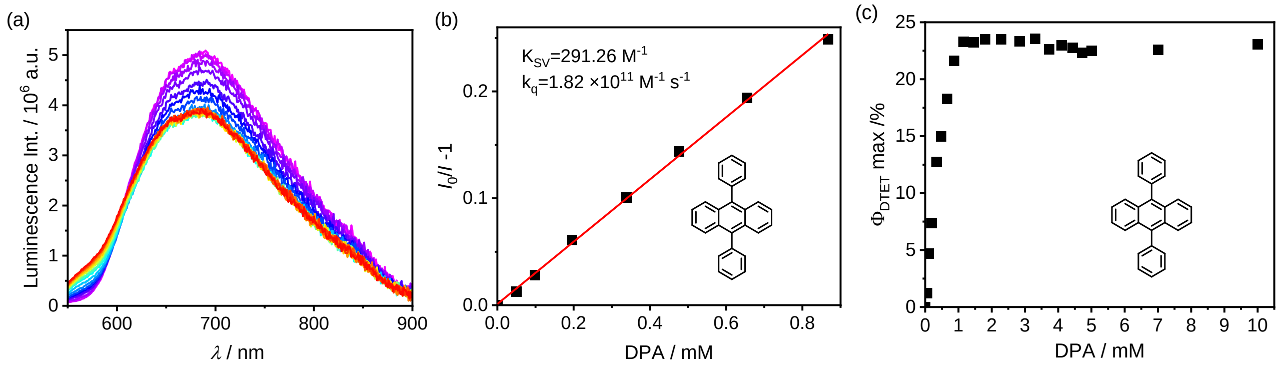

Figure S11. Luminescence intensity-based Stern-Volmer quenching of $[Fe(phtmeimb)_2]PF_6$ by DPA. a) Luminescence spectral changes of the Fe(III) complex (40 μM) upon titration with DPA (0 – 10 mM) in aerated DMSO at 20°C, b) Stern-Volmer plot in the initial linear regime and c) Φ_{DTET} derived from the quenched luminescence intensity from a), plotted as a function of the DPA concentration; linear fitting of the data in b) gives the Stern-Volmer constant K_{SV}, and the initial quenching rate constant k_q ($k_q = K_{SV} / \tau_0$, τ_0 = 1.60 ns from the lifetime obtained in Figure S7b). The rise of luminescence at ~ 570 nm with increasing DPA concentration is attributed to the excimer fluorescence from DPA.[11, 12] $\Phi_{DTET} = 1 - I/I_0$, I_0 and I are the fluorescence intensity values in the absence and presence of DPA in a), giving a DTET efficiency of 22.49% for the $[Fe(phtmeimb)_2]PF_6$ (40 μM)/DPA (10 mM) pair.

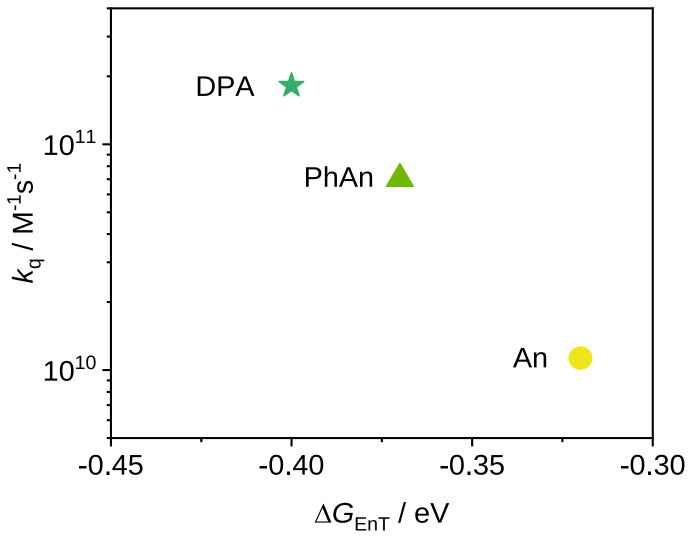

Figure S 12. Plot of the obtained initial luminescence quenching rate constants (k_q, Figure S9 – 11) as a function of the free energy ΔG_{EnT} for DTET from the ^{2}LMCT excited state of Fe(III) complex to the lowest triplet state (T_1) of the employed anthracenes.

The ^{2}LMCT excited state energy is 2.13 eV,[10] which is 0.32 – 0.40 eV above the energetically lowest triplet state (T_1) of the employed anthracenes.[13] Therefore, doublet-triplet energy transfer (DTET) from the ^{2}LMCT state to the T_1 of the anthracenes is thermodynamically feasible.[4, 14] Luminescence intensity-based Stern-Volmer studies of [Fe(phtmeimb)$_2$]PF$_6$ indicate the luminescence quenching by the anthracenes (An, PhAn, and DPA). The derived quenching rate constant k_q increases in the order of Fe(III)/An < Fe(III)/PhAn < Fe(III)/DPA in DMSO (Figure S12). This is because the lowest triplet state energy of the anthracenes decreases in this order, which leads to increasing the free energy ΔG_{EnT} for DTET and thus larger k_q values.[14] Luminescence lifetime-based Stern-Volmer analysis of [Fe(phtmeimb)$_2$]PF$_6$ could not be obtained, because our excitation source (455 nm pulsed LED) for the lifetime measurements of the Fe(III) complex prompts simultaneously the excimer of the anthracenes, and this makes the quantitative analysis of the Fe(III) luminescence decay kinetics under these measurement conditions challenging.

The Stern-Volmer plots derived from the quenched luminescence intensity in section 5.1 – 5.3 follow a linear fit with the quenching rate constant in the region of 11.3 ~ 182 × 10^9 s^{-1}. All these obtained k_q values exceed the diffusion rate constant of DMSO $k_{diff.}$ = 2.9 × 10^9 s^{-1} at 20°C by a factor of 4 ~ 63.[13] Assuming that the DTET occurs as a first-order reaction at the diffusion limit of DMSO and the quencher (Q) concentration is 20 mM (typically used annihilator concentration in our study), the DTET rate is calculated to v(DTET) = $k_{diff.}$ × [Q] = 6.60 × 10^7 M^{-1} s^{-1}. Considering the luminescence lifetime of the Fe(III) complex is 1.60 ns, the inherent excited state decay rate constant is $k_0 = 1 / \tau_0$= 6.25 × 10^8 s^{-1}, which is identical to the natural decay rate v_0 due to the zero-order decay kinetics. The maximal DTET efficiency is calculated Φ_{DTET} = v(DTET) /(v(DTET) + v_0) × 100% = 9.6% for a diffusion-controlled encounter. Supposing the apparent luminescence quenching is all attributable to the energy transfer, the DTET efficiency ($\Phi_{DTET} = 1 - I/I_0$) is determined to 12.3% for Fe(III) (40 μM)/An (20 mM), 14.5% for Fe(III) (40 μM) /PhAn (20 mM), and 22.5% for Fe(III) (40 μM) /DPA (10 mM). These values clearly exceed the maximal DTET efficiency at the diffusion limit. This is likely attributed to pre-association, which occurs non-covalently between a photosensitizer and a quencher in their ground states prior to excitation.[15] Pre-association can occur via coordinative bonding,[16, 17] coulombic interactions,[18-20] hydrogen bonding,[21] or π-π interactions.[22, 23] No clear change in the absorption features of the Fe(III) complex was observed in the presence of the employed anthracenes, which excludes the coordinative bonding. For the Fe(III)/anthracenes pairs, π-π interaction likely occurs between the rich aromatic systems of anthracenes and the phenyl moiety on the backbone of Fe(III) complex. NMR titrations of the exemplary Fe(III)/DPA pair evident the dynamic pre-association in their ground states via π-π interactions (section 6). Such pre-association is beneficial for electron- or energy transfer with an excited state featuring lifetime that is too

short for diffusion-controlled encounter.[15]

The halfwave redox potentials of the employed anthracenes (An, PhAn, and DPA) are found 1.09 – 1.22 V vs SCE,[13] while the excited redox potential of the $[Fe(phtmeimb)_2]PF_6$ (E^o(Fe(III)*/Fe(II))) is 1.37 V vs SCE.[10] Thermodynamically-allowed electron transfer from the anthracenes to the ^{2}LMCT excited state of the Fe(III) complex can not be excluded as an additional excited state quenching mechanism. However, no clear evidence was found for the formation of the radical species with nanosecond-transient absorption measurements (section 7), likely due to the less competitive driving forces ΔG_{ET} for electron transfer than those for energy transfer ΔG_{EnT}. Nonetheless, DTET from the ^{2}LMCT excited state of the Fe(III) complex to the T_1 states of the anthracenes remains the predominant reaction pathway, due to the strong driving force for DTET and the clear evidence for T_1-excited anthracenes obtained from femtosecond- and nanosecond-transient absorption measurements (section 7) and for delayed photon upconversion fluorescence (section 8).

5.4 Spin states in doublet-triplet energy transfer

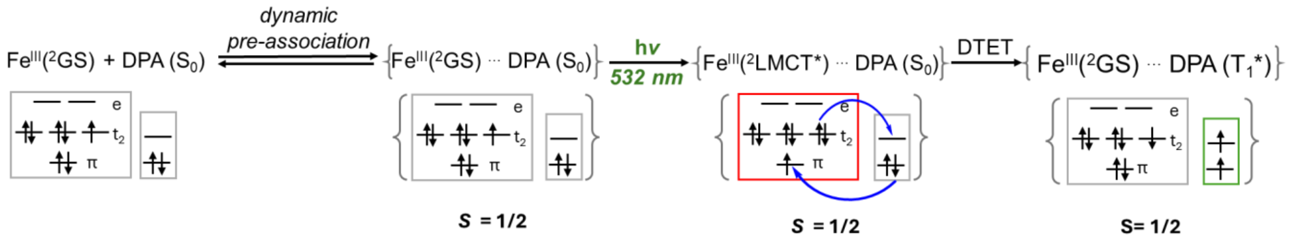

Figure S13. Schematic representation of the dynamic pre-association for Fe(III)/DPA pair in their ground states and the Dexter-type DTET process from the ^{2}LMCT-excited Fe(III) complex to the T_1-state of DPA with their electronic structures. DTET: doublet-triplet energy transfer.

$[Fe(phtmeimb)_2]PF_6$ has an octahedral geometry[10] with a low-spin d^5 configuration in the ground state (Figure S13, left). In the presence of DPA, π-π interactions likely occur between the rich aromatic systems of DPA and the phenyl moieties on the backbone of the Fe(III) complex, as evidenced by the binding between the Fe(III) complex and DPA determined from the NMR titration experiments (section 6). For the pre-associated pair, selective photoexcitation of the Fe(III) complex leads to ligand-to-metal charge transfer, giving the ^{2}LMCT excited state with the fully occupied t_2 orbitals and the half-filled ligand-centered π-orbital (Figure S13, middle part).[24] Dexter-type energy transfer from the ^{2}LMCT-excited Fe(III) complex to DPA occurs via electron exchange (blue arrows in Figure S13, middle part), giving the ground state (^{2}GS) Fe(III) complex and the T_1-excited DPA (Figure S13, right part). Importantly, the total spin S = ½ of the donor/acceptor pair remains constant after the DTET,

making this unusual process spin-allowed according to the Wigner spin conversion rule, which states that the total spin angular momentum (S) of the encounter partner remains unchanged during the transitions.[25-27] The spin multiplicity of the Fe(III) complex is conserved during DTET, whereas the spin of DPA changes from singlet to triplet. This is a very rare example for Dexter-type energy transfer, which typically involves the simultaneous change of the spin multiplicity for the donor and the acceptor, such as TTET from a photosensitizer with triplet metal-to-ligand charge transfer excited state (^{3}MLCT → ^{1}GS) to an organic chromophore ($S_0 \rightarrow T_1$) and DTET from a Cr(III)-based donor with spin-flipped excited states ($^2E/^2T_1 \rightarrow {}^4T_2$) to an organic chromophore ($S_0 \rightarrow T_1$).[4, 28] Nevertheless, the Dexter-type DTET in the Fe(III)/anthracenes pairs is spin-allowed and thermodynamically feasible (section 5.1-3), which provides important fundamental basis for using photoactive Fe(IIII) complex for energy transfer-based applications, such as photon upconversion (section 8).

6. NMR titration for the Fe(III)/DPA pair

For a deeper insight into the interactions between the Fe(III) complex and anthracenes, NMR titration was exemplarily performed with a Bruker Avance AMX-500 operating at 500 MHz proton frequencies by adding DPA (0 – 10 mM) into the DMSO-*d*$_6$ solution of [Fe(phtmeimb)$_2$]PF$_6$ (1 mM) at 293 K.

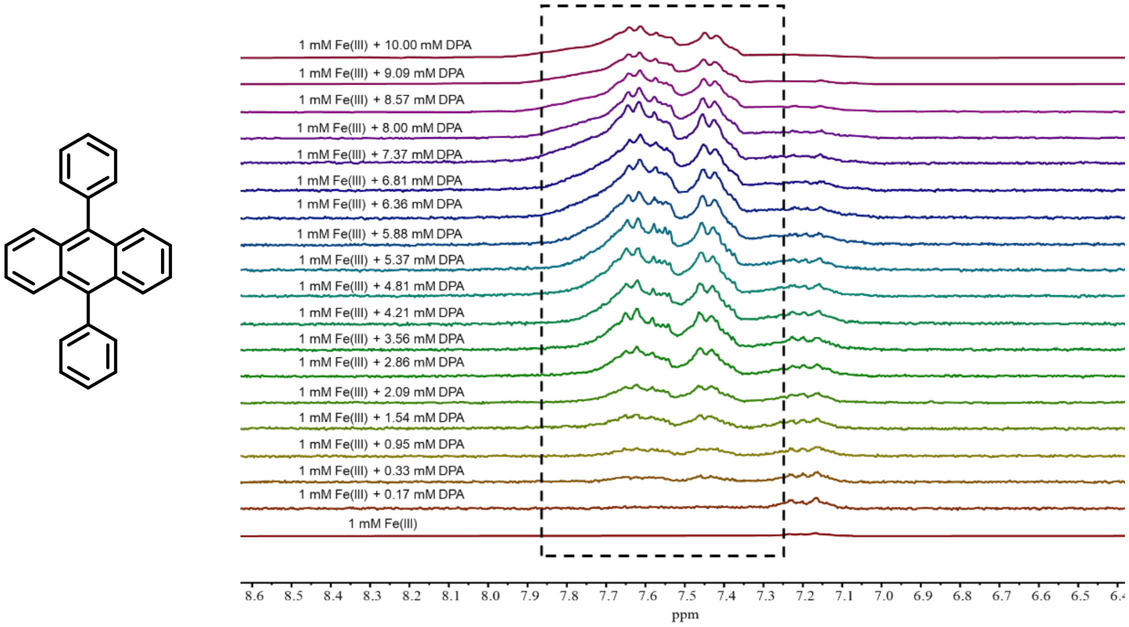

Figure S14. ^{1}H-NMR chemical shift of DPA by adding DPA (0-10 mM) into the DMSO-d$_6$ solution of [Fe(phtmeimb)$_2$]PF$_6$ (1 mM) at 293 K.

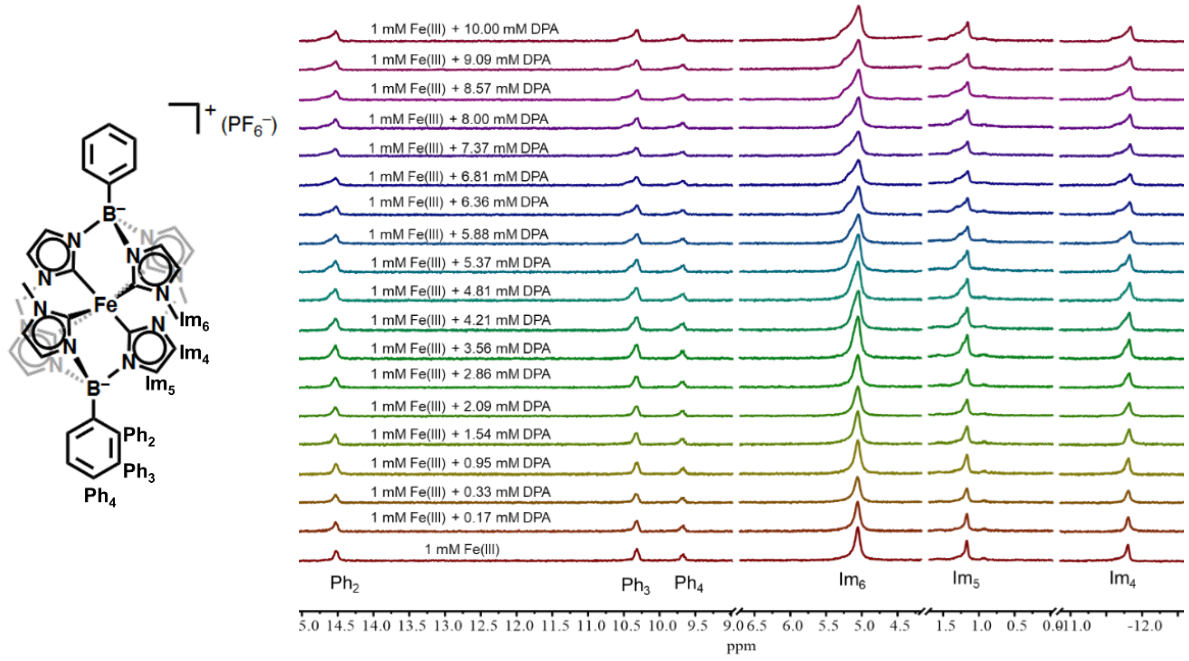

Figure S15. ^{1}H-NMR chemical shift of $[Fe(phtmeimb)_2]PF_6$ by adding DPA (0-10 mM) into the DMSO-d_6 solution of $[Fe(phtmeimb)_2]PF_6$ (1 mM) at 293 K.

Table S1. Summary of the ^{1}H-NMR chemical shifts of protons in $[Fe(phtmeimb)_2]PF_6$ and DPA upon adding DPA (0 – 10 mM) to the DMSO-d_6 solution of $[Fe(phtmeimb)_2]PF_6$ (1 mM) at 293 K.

DPA /mM	Ph_2 [a]	Ph_3 [a]	Ph_4 [a]	Im_6 [a]	Im_5 [a]	Im_4 [a]	DPA_1 [b]
0	14.528627	10.314524	9.668493	5.063667	1.171169	-11.799530	0
0.17	14.529295	10.315568	9.667749	5.060973	1.169879	-11.804055	7.442234
0.33	14.529963	10.316612	9.666632	5.058952	1.167944	-11.809404	7.435496
0.95	14.530631	10.318187	9.665888	5.057604	1.166654	-11.811461	7.434526
4.54	14.532970	10.320266	9.665144	5.056257	1.165364	-11.817632	7.433563
2.09	14.534640	10.322355	9.662539	5.054910	1.164074	-11.818455	7.432599
2.86	14.535308	10.323921	9.661795	5.052889	1.162139	-11.819689	7.430191
3.56	14.535977	10.324965	9.661051	5.051542	1.160849	-11.821335	7.429227
4.21	14.535977	10.326009	9.660188	5.048847	1.160801	-11.822158	7.428263
4.81	14.535977	10.321311	9.659934	5.046826	1.159558	-11.823392	7.427302
5.37	14.535977	10.318178	9.65919	5.045479	1.158268	-11.825038	7.425855
5.88	14.535977	10.316612	9.658446	5.044132	1.156978	-11.825861	7.424891
6.36	14.535977	10.315568	9.657702	5.042784	1.155043	-11.826684	7.422964
6.81	14.535977	10.313479	9.657381	5.041437	1.155043	-11.828741	7.423928
7.37	14.535977	10.311913	9.656957	5.039416	1.154398	-11.830386	7.421519
8.00	14.535977	10.310869	9.656257	5.038069	1.153108	-11.834301	7.420555
8.57	14.535977	10.310119	9.655733	5.036721	1.15214	-11.839849	7.419592
9.09	14.535977	10.309825	9.655414	5.035732	1.151495	-11.840667	7.417665
10.00	14.535977	10.306170	9.655019	5.034561	1.150205	-11.842443	7.416942

[a] The signals chosen for the fitting are the protons of $[Fe(phtmeimb)_2]PF_6$ defined in Figure S14, which could participate in the π-π interaction with DPA.

[b] The proton signals on DPA, due to the broadening of the peak, the most obvious hydrogen proton in the multi-peaks is selected for the fitting.

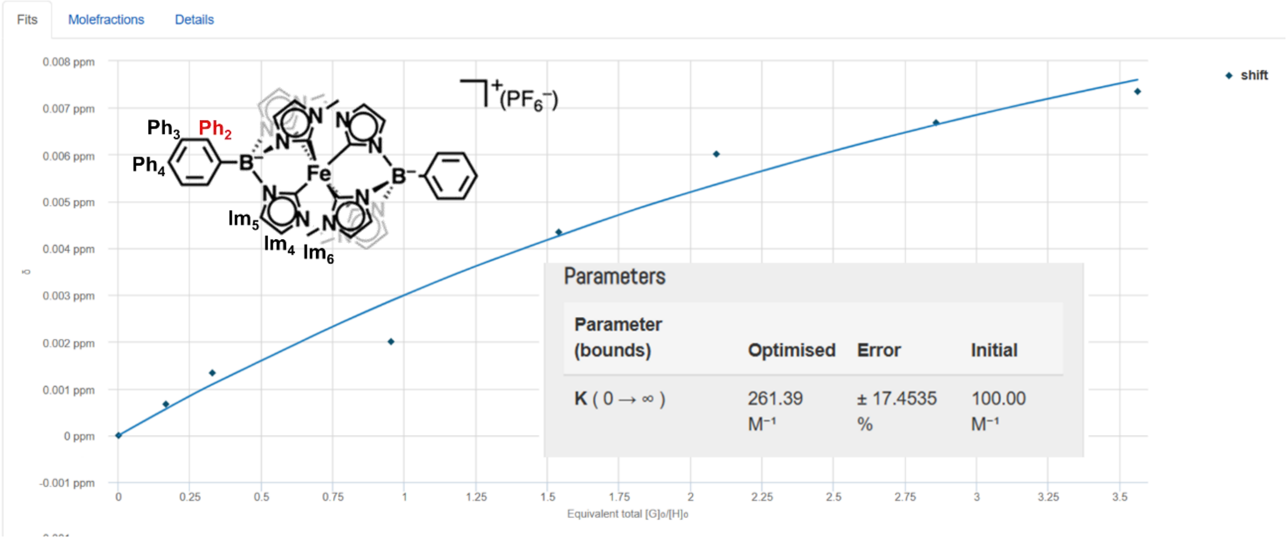

Figure S16. Nonlinear least-squares fitting results based on the chemical shifts of the Ph_2 proton of the Fe(III) complex (red label in the Fe(III) complex structure) in the presence of DPA (0 – 10 mM) following a 1:1 stoichiometry. This gives a binding constant of 261 M^{-1} for the Ph_2 proton with DPA.

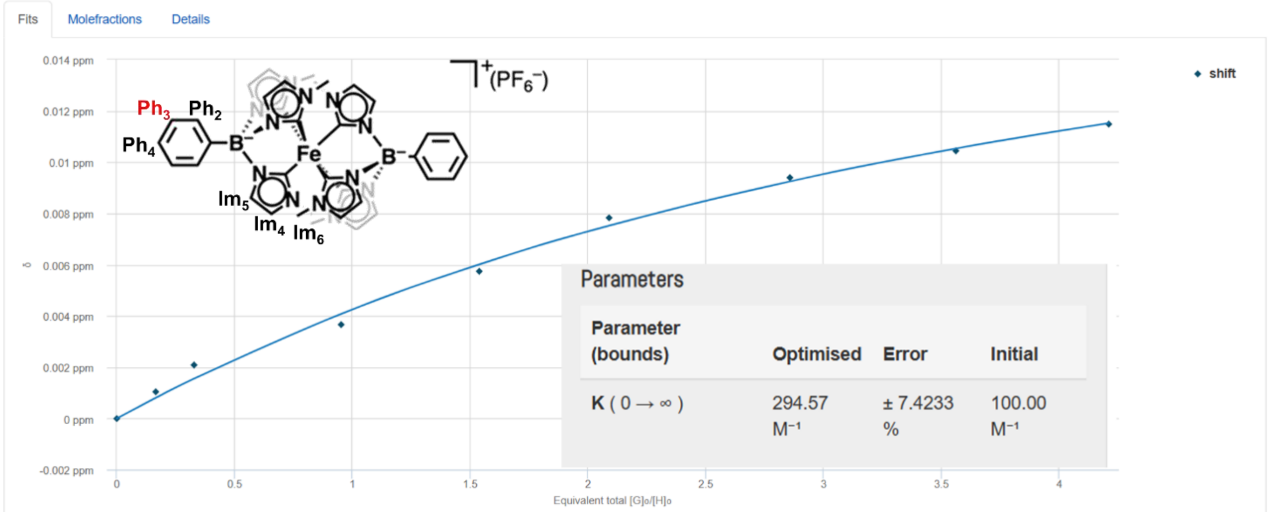

Figure S17. Nonlinear least-squares fitting results based on the chemical shifts of the Ph_3 proton of the Fe(III) complex (red label in the Fe(III) complex structure) in the presence of DPA (0 – 10 mM) following a 1:1 stoichiometry. This gives a binding constant of 295 M^{-1} for the Ph_3 proton with DPA.

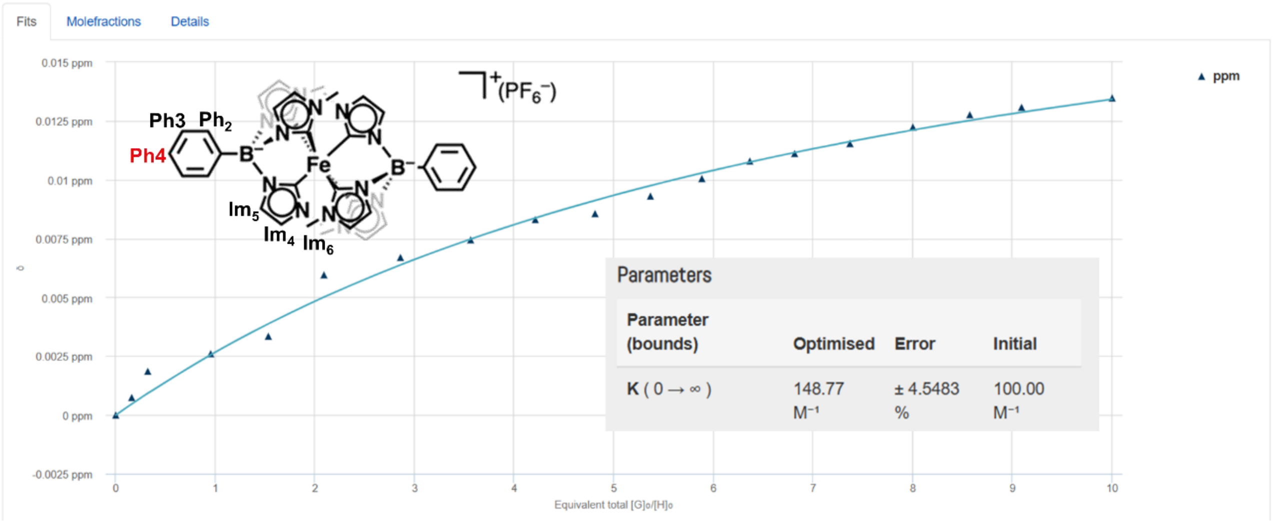

Figure S18. Nonlinear least-squares fitting results based on the chemical shifts of the Ph_4 proton of the Fe(III) complex (red label in the Fe(III) complex structure) in the presence of DPA (0 – 10 mM) following a 1:1 stoichiometry. This gives a binding constant of 149 M^{-1} for the Ph_4 proton with DPA.

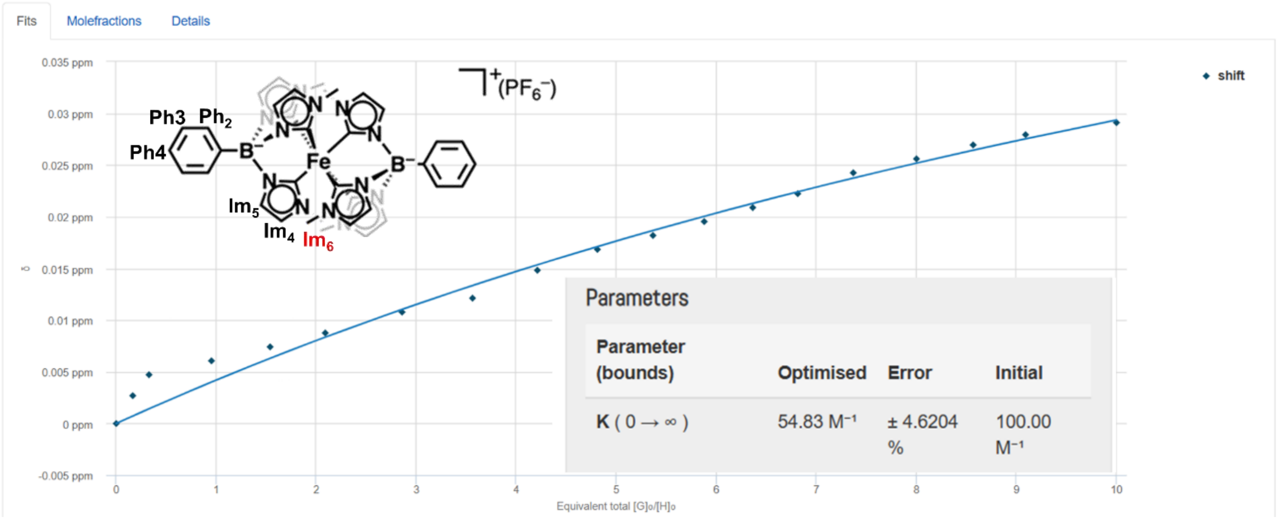

Figure S19. Nonlinear least-squares fitting results based on the chemical shifts of the Im_6 proton of the Fe(III) complex (red label in the Fe(III) complex structure) in the presence of DPA (0 – 10 mM) following a 1:1 stoichiometry. This gives a binding constant of 55 M^{-1} for the Im_6 proton with DPA.

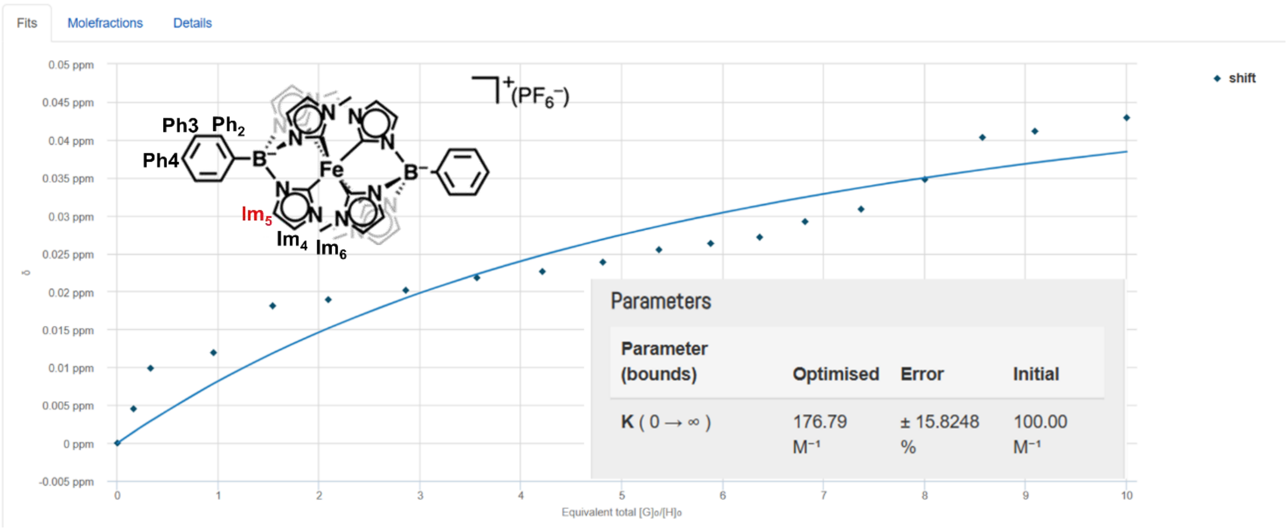

Figure S20. Nonlinear least-squares fitting results based on the chemical shifts of the Im_5 proton of the Fe(III) complex (red label in the Fe(III) complex structure) in the presence of DPA (0 – 10 mM) following a 1:1 stoichiometry. This gives a binding constant of 177 M^{-1} for the Im_5 proton with DPA.

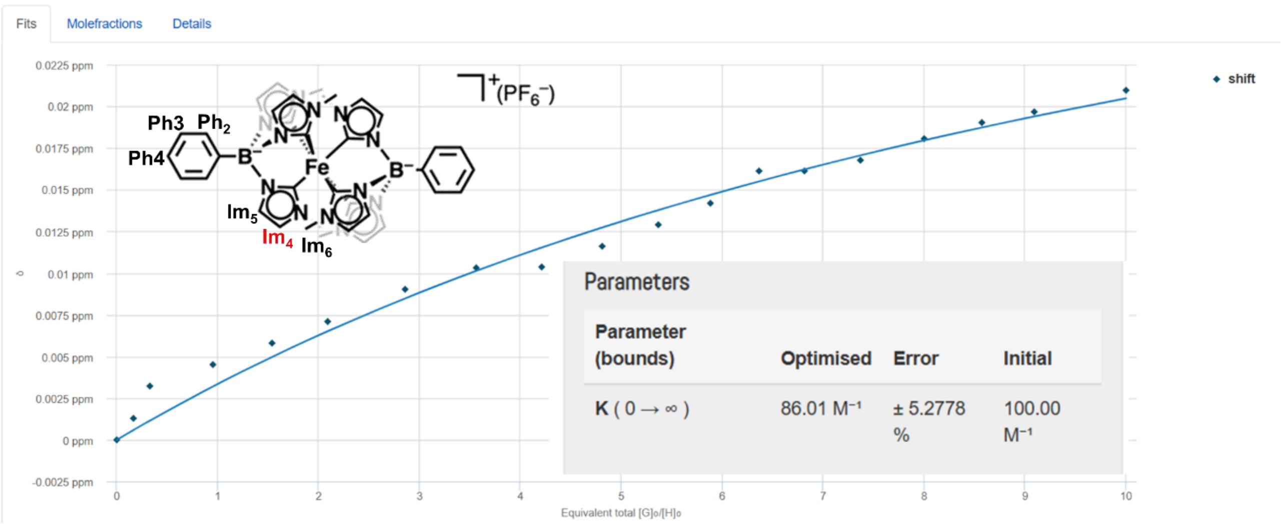

Figure S21. Nonlinear least-squares fitting results based on the chemical shifts of the Im_4 proton of the Fe(III) complex (red label in the Fe(III) complex structure) in the presence of DPA (0 – 10 mM) following a 1:1 stoichiometry. This gives a binding constant of 86 M^{-1} for the Im_4 proton with DPA.

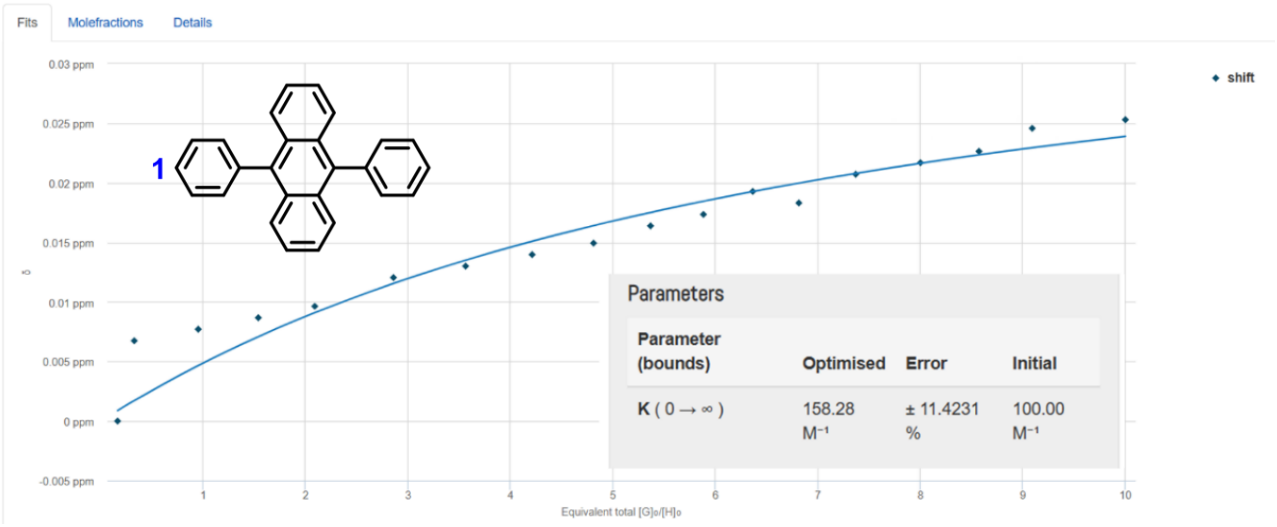

Figure S22. Nonlinear least-squares fitting results based on the chemical shifts of the DPA_1 proton of the Fe(III) complex (red label in the Fe(III) complex structure) in the presence of DPA (0 – 10 mM) following a 1:1 stoichiometry. This gives a binding constant of 158 M^{-1} for the DPA_1 proton with DPA.[29]

The ^{1}H-NNR titration experiment[1] was conducted to study the interactions between the [Fe(phtmeimb)$_2$]PF_6 and DPA in their ground states. By adding DPA with increasing concentrations to the DMSO-d_6 solution of [Fe(phtmeimb)$_2$]PF_6, both the proton peaks of DPA and the Fe(III) complex exhibit slight but recognizable changes (Figure S14 and 15, Table S1). Using a 1:1 binding model for the Fe(III)/DPA pair, nonlinear least-squares fitting of the chemical shifts gives a binding constant K ranges from 148 to 295 M^{-1} for the phenyl moiety of the Fe(III) complex, and a K of 177 M^{-1} was obtained for the close-standing Im_5 proton on the imidazole ring. These binding constants are found in the same regime as that obtained with the proton chemical shift from DPA (K = 158 M^{-1}). Unlike the host-guest interactions/ inclusion interactions that show typically significant changes in the chemical shift and large binding constants,[30-33] the magnitudes of these binding constants correspond to weak π-π interactions between the Fe(III) complex and DPA that occur in a reversible/dynamic manner, as previously reported in the literature.[1, 34] No observable change in the absorption features of the Fe(III) complex was detected in the presence of the anthracenes, pointing to the absence of coordinative interactions.

7. Transient absorption (TA) measurements

7.1 fs - TA spectroscopy of the Fe(III)/DPA pair

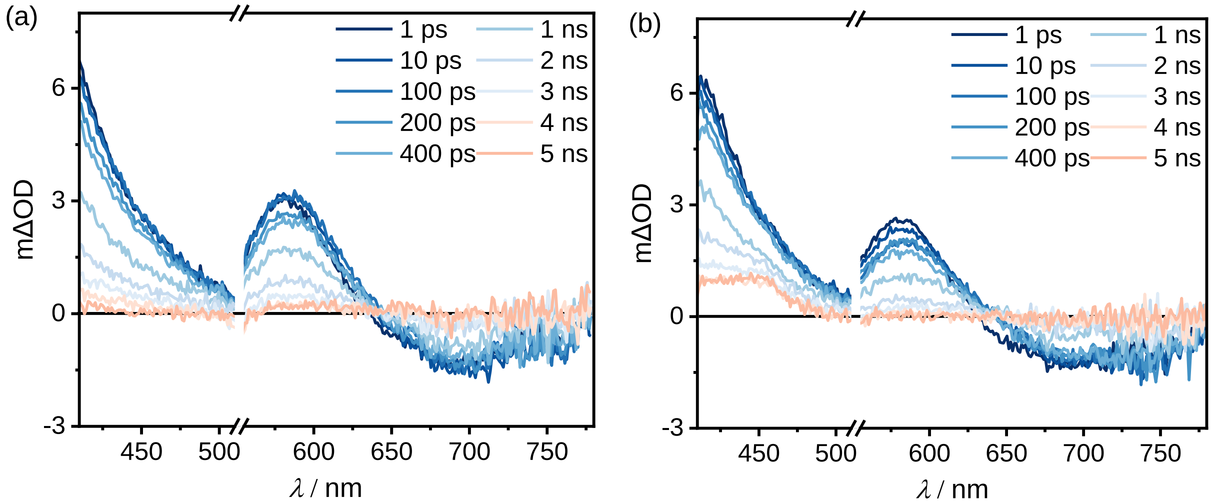

Figure S23. UV-vis transient absorption spectra of a) [Fe(phtmeimb)$_2$]PF$_6$ (500 μM) and b) [Fe(phtmeimb)$_2$]PF$_6$ (500 μM)/DPA (10 mM) in aerated DMSO at 293 K with different time delays after excitation at 532 nm with a fs-pulsed laser (pulse energy ~ 0.92 μJ). The wavelength region between 510 nm and 555 nm is removed due to the pump scattering.

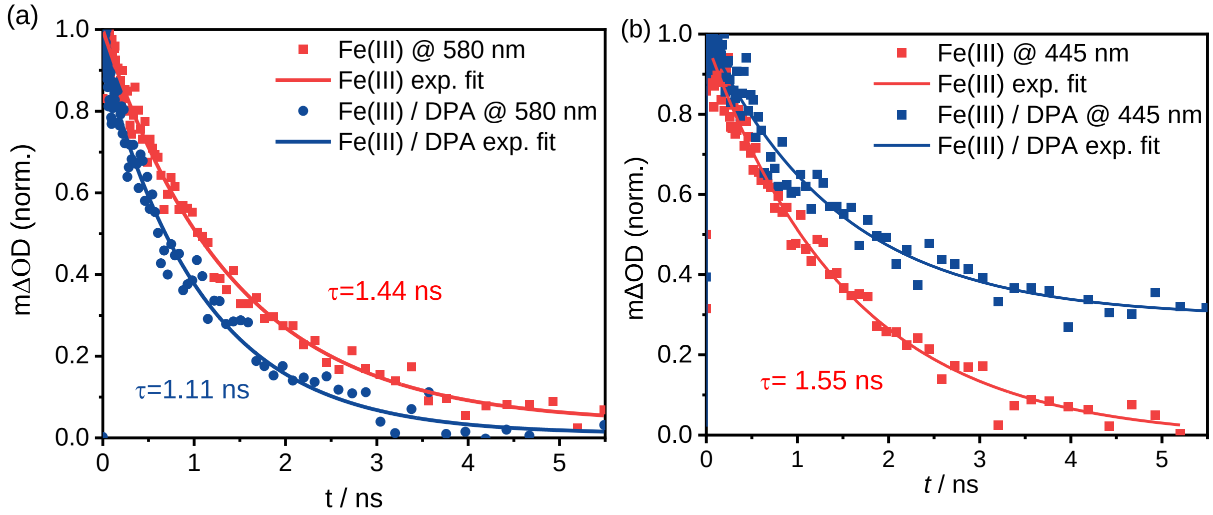

Figure S24. Transient absorption decays of [Fe(phtmeimb)$_2$]PF$_6$ (500 μM) (red traces) and [Fe(phtmeimb)$_2$]PF$_6$ (500 μM)/DPA (10 mM) (blue traces) in aerated DMSO recorded at 580 nm (a) and 445 nm (b), together with their mono-exponential or bi-exponential fits. Excitation occurred with a fs-pulsed laser at 532 nm (pulse energy ~ 0.92 μJ). $\Phi_{DTET} = 1 - \tau/\tau_0$, τ_0 and τ are the lifetimes of [Fe(phtmeimb)$_2$]PF$_6$ in the absence and presence of DPA at 580 nm, giving a DTET efficiency of 22.92% for the [Fe(phtmeimb)$_2$]PF$_6$ (40 μM)/DPA(10 mM) pair.

Upon selective excitation of the [Fe(phtmeimb)$_2$]PF$_6$ (500 μM) at 532 nm, the initially formed excited state absorption signal of [Fe(phtmeimb)$_2$]PF$_6$ at 580 nm[10, 35] follows a mono-exponential decay with a time constant of 1.44 ns (Figure S23a and S24a), which agrees well with that obtained at 445 nm (1.55 ns) (Figure S24b) and the previously obtained luminescence lifetime (Figure S7b). Selective excitation of the [Fe(phtmeimb)$_2$]PF$_6$ (500 μM)/DPA (10 mM) pair with fs-pulsed laser at 532 nm showed that the transient absorption signal at 580 nm decays faster with a time constant of 1.11 ns (Figure S23b and 24a). This corresponds to a DTET efficiency of 22.92%, which is identical to the DTET efficiency obtained from the luminescence intensity quenching of DPA (Section 5.3, Table S2). This agreement indicates that the excited state quenching is exclusively caused by the DTET and the above-mentioned pre-association does not lead to luminescence quenching. In this multistep process, pre-association of the Fe(III) complex and DPA occurs in their ground state, followed by DTET in the pre-associated association complex. Such quenching process is often called "static" to emphasize that it does not rely on diffusion.[15] But this can not be assigned to the classical "static quenching", in which the luminescence lifetime of the chromophore is not affected. For such multistep process, the simple cases of static or dynamic quenching do not readily apply, similar to the bimolecular quenching events occurring in nanoconfiments.[36] Meanwhile, the gradual formation of the spectral signature of the triplet excited state of DPA (^{3}DPA*) at around 450 nm (Figure S23b),[4, 13] which decays significantly slower than the transient absorption signal of [Fe(phtmeimb)$_2$]PF$_6$ and reaches a plateau around 3 ns after the laser excitation (Figure S24b, blue traces). This provides an insight into the kinetics for the DTET from the ^{2}LMCT excited state of the Fe(III) complex to ^{3}DPA*.

7.2 ns - TA spectroscopy of the Fe(III)/An pair

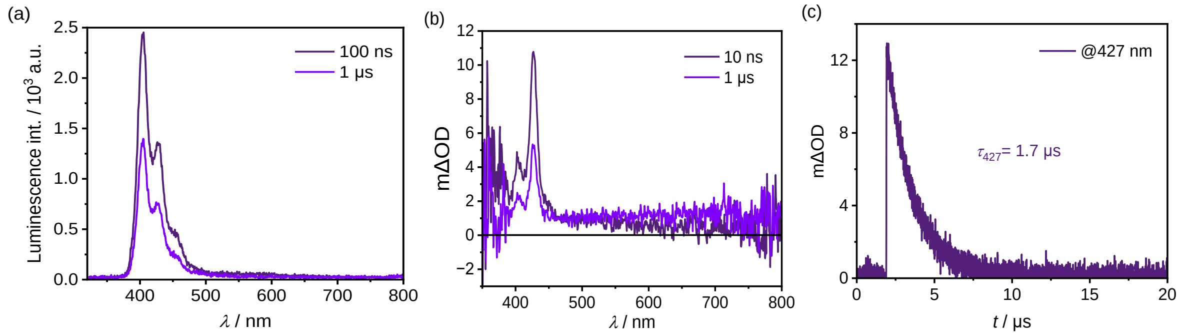

Figure S25. a) Luminescence spectra and b) UV-vis transient absorption spectra of [Fe(phtmeimb)$_2$]PF$_6$ (100 μM)/An (10 mM) in aerated DMSO at 293 K with different time delays after excitation at 532 nm with ns-pulsed laser (pulse energy ~ 12 mJ). c) Transient absorption decay at 427 nm of the sample from b). Mono-exponential fit of the decay gives a lifetime of 1.7 μs.

Selective excitation of the $[Fe(phtmeimb)_2]PF_6$/An pair in aerated DMSO at 532 nm gives the upconversion luminescence with a maximum at 407 nm from the singlet excited state (S_1) of An, which is still observable 1 µs after laser excitation, due to the delayed nature of upconversion luminescence (Figure S25 a). UV-vis nanosecond-transient absorption spectra show the finger-structured features of the T_1-excited state of An maximized at 427 nm (Figure S25 b),[13] which follows a mono-exponential decay with a time constant of 1.7 µs (Figure S25 c). This reflects the inherent long lifetime of the T_1 state of An and evidences the occurrence of DTET.[13]

7.3 ns - TA spectroscopy of the Fe(III)/PhAn pair

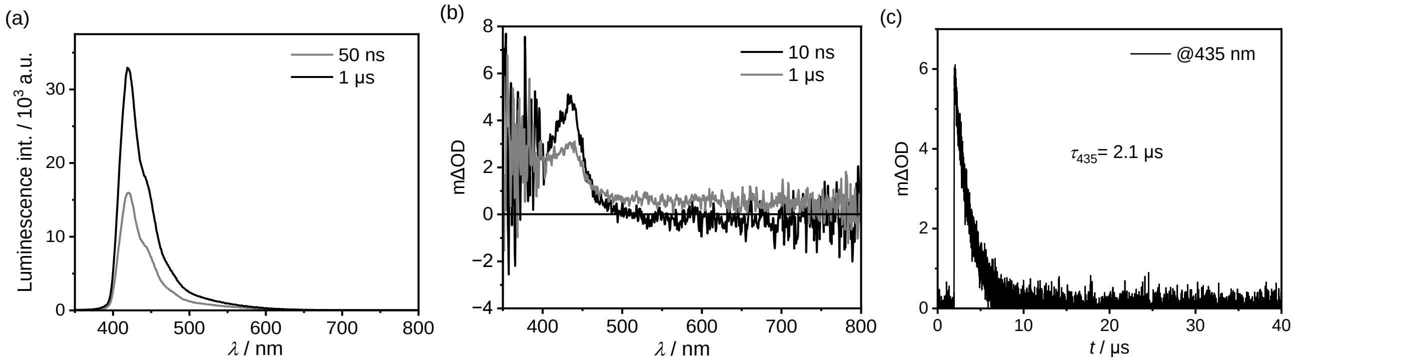

Figure S26. a) Luminescence spectra and b) UV-vis transient absorption spectra of $[Fe(phtmeimb)_2]PF_6$ (100 µM)/PhAn (10 mM) in aerated DMSO at 293 K with different time delays after excitation at 532 nm with ns-pulsed laser (pulse energy ~ 12 mJ). c) Transient absorption decay at 435 nm of the sample from b). Mono-exponential fit of the decay gives a lifetime of 2.1 µs.

Selective excitation of the $[Fe(phtmeimb)_2]PF_6$/PhAn pair in aerated DMSO at 532 nm gives the upconversion luminescence with a maximum at 420 nm from the singlet excited state (S_1) of PhAn, which is observable 1 µs after laser excitation due to the delayed nature of upconversion luminescence (Figure S26 a). UV-vis nanosecond-transient absorption spectra show the spectral feature of the T_1-excited state of PhAn maximized at 435 nm (Figure S26 b),[13] which follows a mono-exponential decay with a time constant of 2.1 µs (Figure S26 c). This corresponds to the inherent long lifetime of the T_1 state of PhAn and evidences the occurrence of DTET.[13]

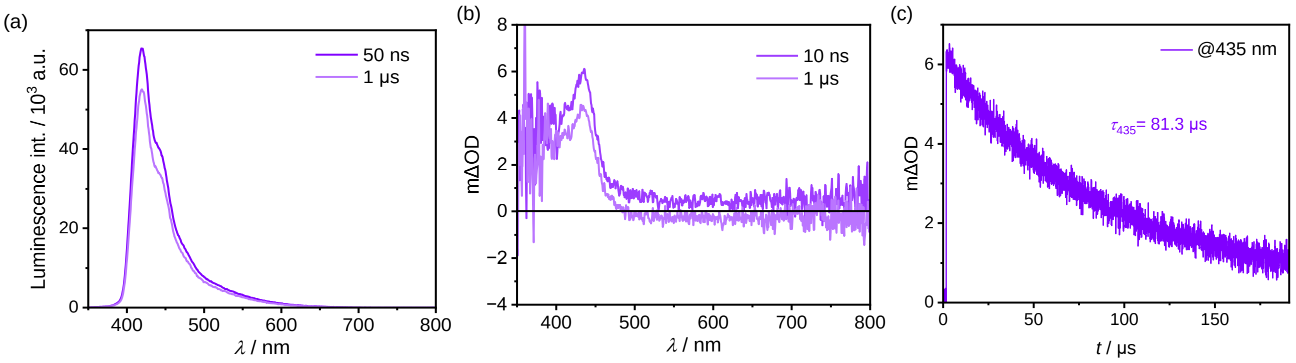

Figure S27. a) Luminescence spectra and b) transient absorption spectra of $[Fe(phtmeimb)_2]PF_6$ (100 µM)/PhAn (10 mM) in the presence of the mediator An (500 µM) in aerated DMSO at 293 K with different time delays after excitation at 532 nm with ns-pulsed laser (pulse energy ~ 12 mJ). c) Transient absorption decay at 435 nm of the sample from b). Mono-exponential fit of the decay gives a lifetime of 81.3 µs.

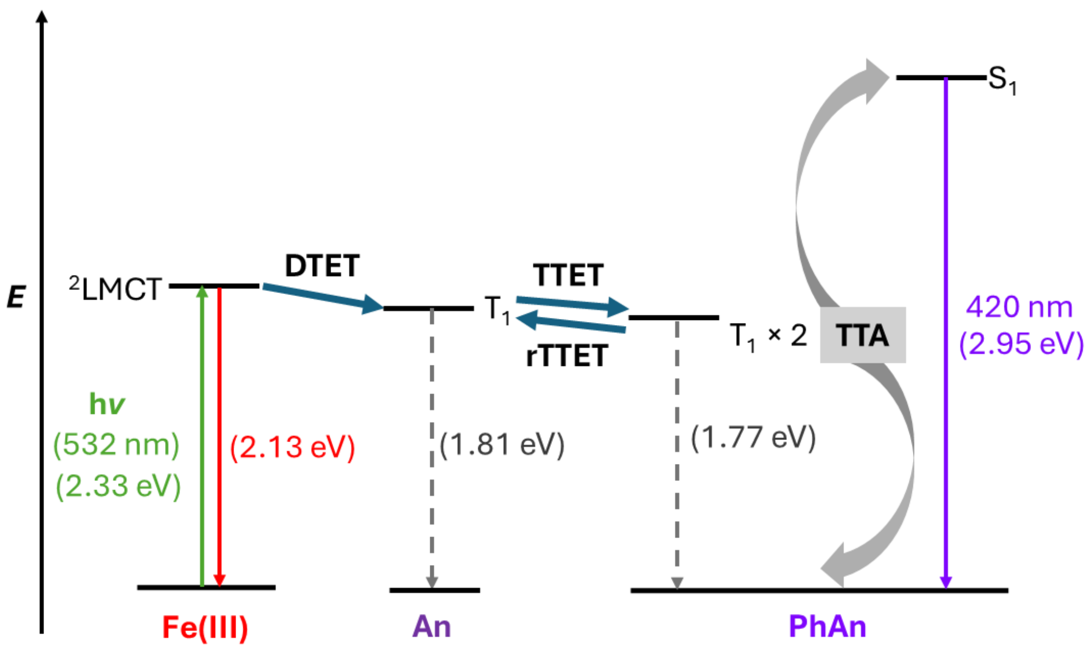

Figure S28. Energy-level diagram for the sTTA-UC occurring in the $[Fe(phtmeimb)_2]PF_6$/PhAn pair with An as the mediator. Selective excitation of the Fe(III) complex at 532 nm leads to delayed upconversion fluorescence from PhAn at 420 nm. DTET: doublet-triplet energy transfer; TTET: triplet-triplet energy transfer; rTTET: reverse triplet-triplet energy transfer; TTA: triplet-triplet annihilation.

Recent reports show that sTTA-UC system can be enhanced by introducing a mediator, which usually features a triplet excited state with lifetime longer than that of the photosensitizer and energy level in-between the photosensitizer and the annihilator.[20, 37] This strategy is

particularly attractive for the Fe(III) complex sensitized sTTA-UC, because the short ^{2}LMCT excited state lifetime is then to some extent compensated by the long triplet excited state lifetime of the mediator. For the [Fe(phtmeimb)$_2$]PF$_6$/PhAn upconversion pair, An can be introduced as a mediator between the Fe(III) photosensitizer and PhAn, due to the suitable energy level and the long lifetime of the T_1 state of An. The general mechanism of this system is as follows: Excitation of the sensitizer populates the ^{2}LMCT state of the Fe(III) complex, which is followed by a DTET to the T_1 state of An. Subsequent TTET from An to PhAn gives triplet excited PhAn, from which triplet-triplet annihilation occurs and gives the singlet excited PhAn and consequently delayed fluorescence. In the presence of An as the mediator, the transient absorption signal of the triplet PhAn at 435 nm is extended from 2.1 μs to 81.3 μs (Figure S26c and 27c). Considering the fact that the T_1 state of An is only 0.04 eV higher than that of PhAn, a reverse TTET from PhAn to An seems viable with thermal energy (Figure S28). This forms a so-called "triplet reservoir effect" between the An and PhAn, which is a popular strategy to extend triplet excited state lifetime. Consequently, An and PhAn engage in an excited state equilibrium due to their long-excited state lifetimes and similar excited state energies, and this accounts for the significantly elongated T_1 excited state lifetime of PhAn. However, efficient reverse TTET can act as a competing pathway to TTA, which could weaken the upconversion luminescence (section 9.1). The decay kinetics of upconversion luminescence amounts typically to half of the T_1-state lifetime of the annihilator.[38-40] This correlation agrees with our observation that the upconversion luminescence shows a slower decay in the presence of An as the mediator (Figure S26a and 27a).

7.4 ns - TA spectroscopy of the Fe(III)/DPA pair

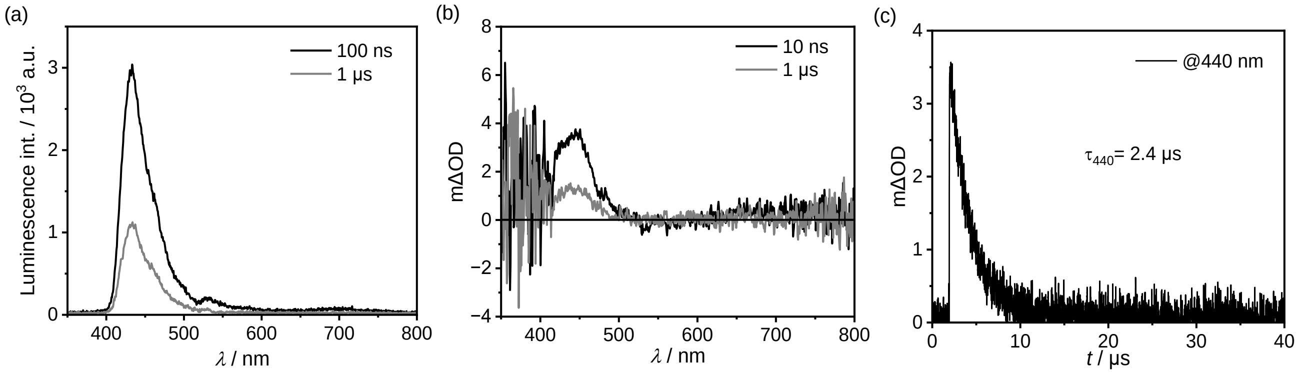

Figure S29. a) Luminescence spectra and b) UV-vis transient absorption spectra of [Fe(phtmeimb)$_2$]PF$_6$ (100 μM)/DPA (10 mM) in aerated DMSO at 293 K with different time delays after excitation at 532 nm with ns-pulsed laser (pulse energy ~ 12 mJ). c) Transient absorption decay at 440 nm of the sample from b). Mono-exponential fit of the decay gives a lifetime of 2.4 μs.

Selective excitation of the [Fe(phtmeimb)$_2$]PF$_6$/DPA pair in aerated DMSO at 532 nm gives the upconversion luminescence with a maximum at 430 nm from the singlet excited state (S$_1$) of DPA, which is observable 1 μs after laser excitation due to the delayed nature of upconversion (Figure S29a). UV-vis nanosecond-transient absorption spectra show the spectral feature of the T$_1$-excited state of DPA maximized at 440 nm (Figure S29b),[13] which follows a mono-exponential decay with a time constant of 2.4 μs (Figure S29c). This corresponds to the inherent long lifetime of the T$_1$-excited state of DPA and evidences the occurrence of DTET.[4, 13]

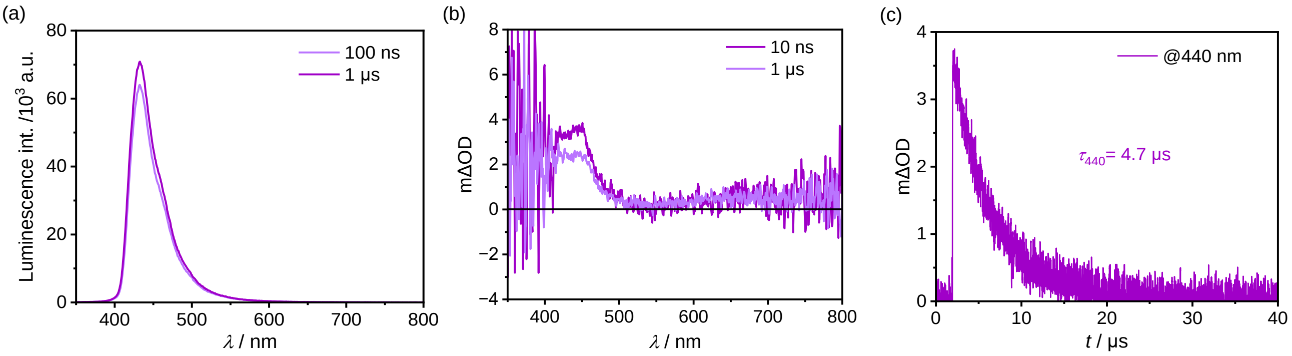

Figure S30. a) Luminescence spectra and b) UV-vis transient absorption spectra of [Fe(phtmeimb)$_2$]PF$_6$ (100 μM)/DPA (10 mM) in the presence of the mediator An (500 μM) in aerated DMSO at 293 K with different time delays after excitation at 532 nm with ns-pulsed laser (pulse energy ~ 12 mJ). c) Transient absorption decay at 440 nm of the sample from b). Mono-exponential fit of the decay gives a lifetime of 4.7 μs.

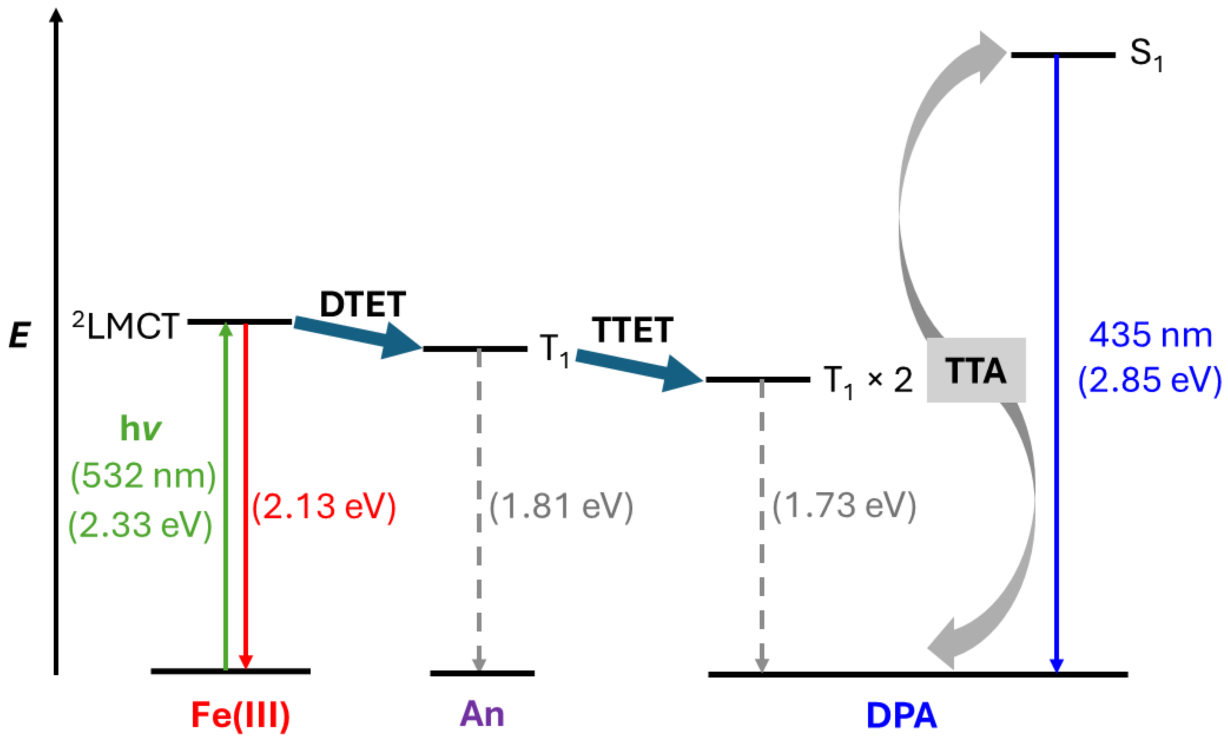

Figure S31. Energy-level diagram for the sTTA-UC occurring in the $[Fe(phtmeimb)_2]PF_6$/DPA pair with An as the mediator. Selective excitation of the Fe(III) complex at 532 nm leads to delayed upconversion fluorescence from DPA at 430 nm. DTET: doublet-triplet energy transfer; TTET: triplet-triplet energy transfer; TTA: triplet-triplet annihilation.

For the $[Fe(phtmeimb)_2]PF_6$/DPA upconversion pair, the presence of the An mediator extends the T_1- excited state lifetime from 2.4 to 4.7 μs for the DPA annihilator, which leads to slower decay of the upconversion luminescence (Figure S29a and S30a). In comparison to the An-mediated $[Fe(phtmeimb)_2]PF_6$/PhAn pair, this smaller change in lifetime is attributed to the larger energy gap between the T_1 states of An and DPA, which suppresses the reverse TTET and the formation of significant "triplet reservoir". Eventually, enhanced upconversion performance was observed for the An-mediated Fe(III)/DPA pair (section 9.2).

8. Photon upconversion studies

8.1 Excitation power density dependence of [Fe(phtmeimb)$_2$]PF$_6$

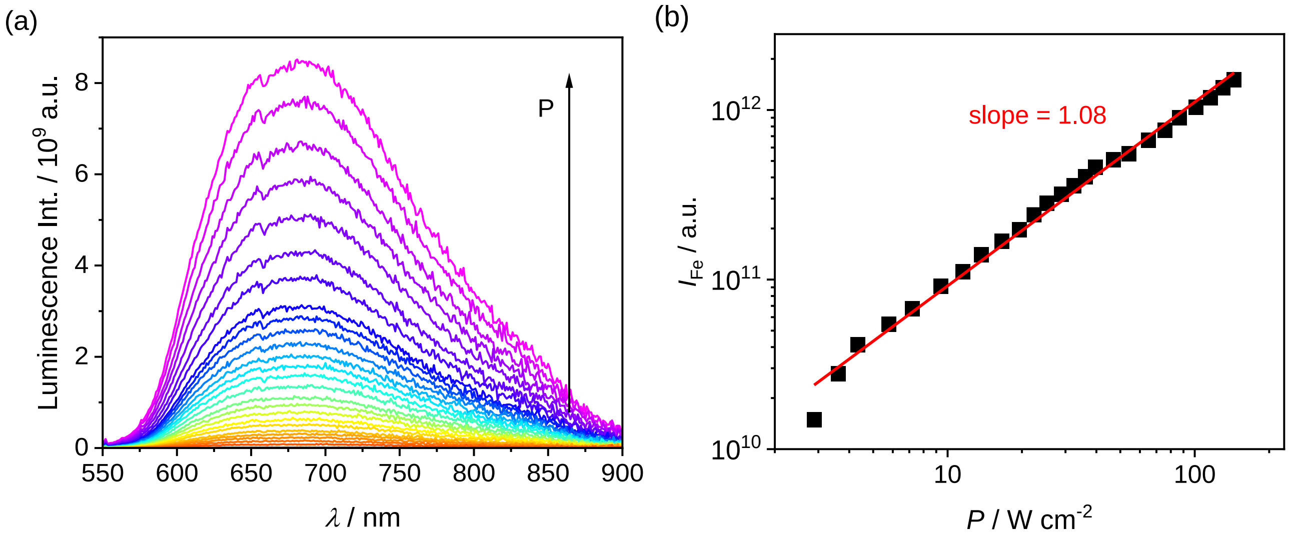

Figure S32. a) Luminescence spectra of 40 µM [Fe(phtmeimb)$_2$]PF$_6$ in aerated DMSO at 293 K with different excitation power (4 mW to 200 mW) from a tunable 532 nm-cw laser. A 495 nm long pass filter was placed between the laser and the sample. b) Excitation power density dependence of the luminescence intensity integral from 550 – 900 nm extracted from a), plotted double logarithmically against the excitation power density of the laser. The prompt luminescence of [Fe(phtmeimb)$_2$]PF$_6$ shows a linear dependence on the excitation power density of the 532 nm cw-laser.

8.2 Control experiments

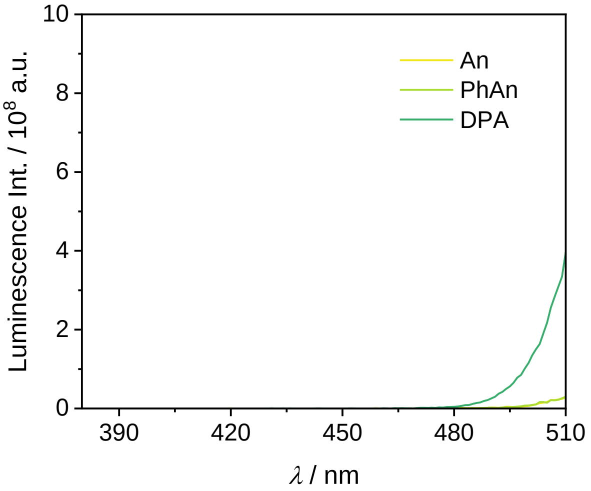

Figure S33. Luminescence spectra of An (20 mM), PhAn (20 mM), and DPA (10 mM) in aerated DMSO at 293 K, excited with a green 532 nm cw-laser at 200 mW. A 495 nm long pass filter was placed between the laser and the sample.

To ensure that all luminescence of anthracene derivatives annihilators comes from the TTA process rather than direct excitation, steady-state fluorescence measurements were conducted at 532 nm laser excitation with samples containing the same concentration of annihilator as in the upconversion measurements (An, 20 mM; PhAn, 20 mM and DPA, 10 mM) but without $[Fe(phtmeimb)_2]PF_6$, as shown in Figure S33. The characteristic anthracene fluorescence was not observed below 480 nm, and the slight rise of signals above 480 nm is attributed to the emission tail from the anthracene excimers formed at these high concentrations,[11, 12] as seen in Figure 2a ~ 560 nm in the main paper. Despite the longer irradiation wavelength at 532 nm, the observation of fluorescence signal arising from 480 nm is not attributable to an upconversion process, because the fluorescence is a tailed signal from the broad emission band of the excimers that is partially prompt with our 200 mW 532 nm cw-laser.[11, 12]

8.3 Fe(III) + An

Excitation power density dependence

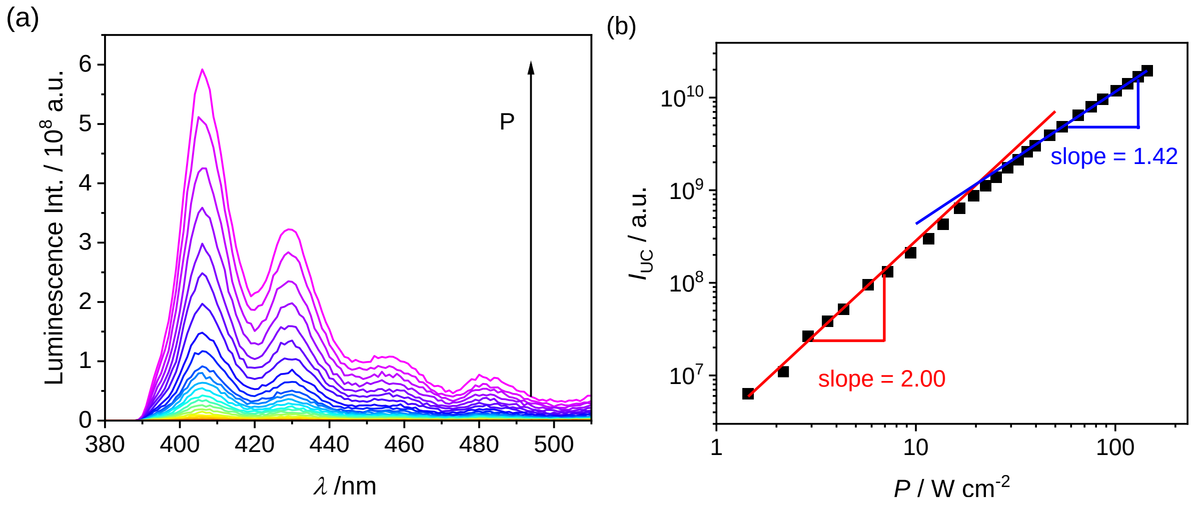

Figure S34. a) Upconversion luminescence intensity of $[Fe(phtmeimb)_2]PF_6$ (40 µM)/An (20 mM) in aerated DMSO excited with a green 532 nm cw-laser at different laser powers (2 mW to 200 mW). A 495 nm long pass filter was placed between the laser and the sample. b) Excitation power density dependence of the upconversion luminescence integral from 380 to 510 nm extracted from a) as a log-log plot. Linear fit gives a slope of 2.00 at low excitation power densities and 1.42 at high excitation power densities.

For the $[Fe(phtmeimb)_2]PF_6$ (40 µM)/An (20 mM) pair, the intrinsic luminescence band at 390 nm from the S_1-state of An (Figure S4a) becomes a weak shoulder (Figure S34a), due to the reabsorption phenomena with such high concentration of the annihilator.[40-42] Excitation power density study of the upconversion luminescence shows the biphotonic process (slope of 2.00) at low excitation power densities, which tends to reach a saturation at high power densities. However, the upconversion saturation was not achieved under our conditions, mainly due to the very short ^{2}LMCT excited state lifetime that shifts the I_{th} to high values.[40, 43]

Upconversion quantum yields

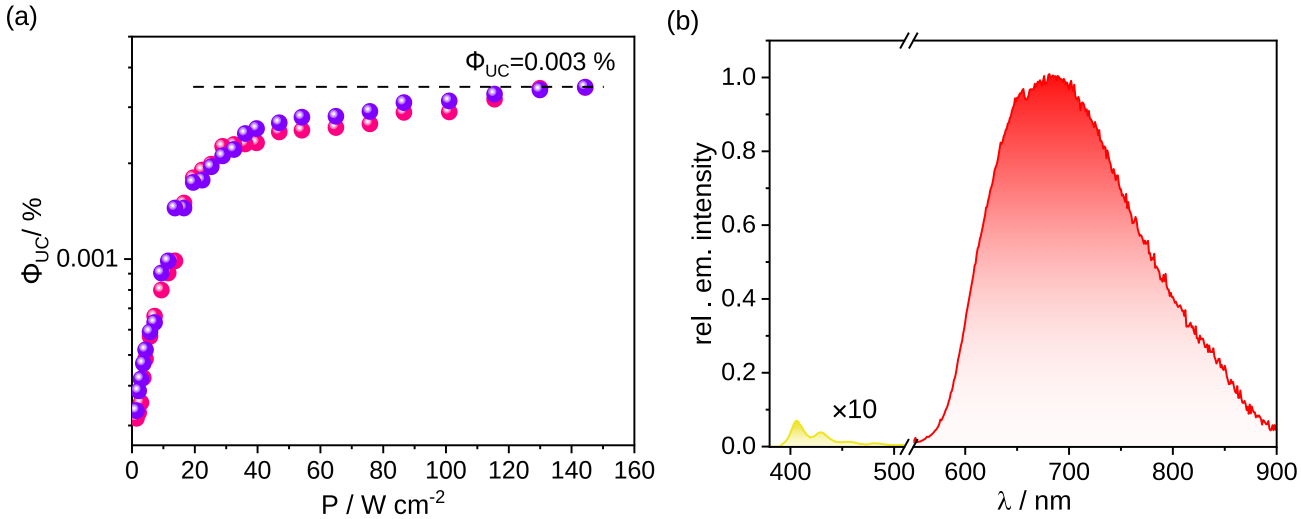

Figure S 35. a) Upconversion luminescence quantum yields (Φ_{UC}) of [Fe(phtmeimb)$_2$]PF$_6$ (40 μM)/An (20 mM) in aerated DMSO at 293 K as a function of the excitation power density (532 nm cw-laser). A 495 nm long pass filter was placed between the laser and the sample. These measurements were carried out independently for twice on different days. A maximal upconversion quantum yield Φ_{UC} of 0.003% was obtained for the [Fe(phtmeimb)$_2$]PF$_6$ (40 μM)/An (20 mM) pair under our conditions. b) Upconversion luminescence spectrum (chartreuse) of the [Fe(phtmeimb)$_2$]PF$_6$ (40 μM)/An (20 mM) pair and the prompt luminescence spectrum (red) of the [Fe(phtmeimb)$_2$]PF$_6$ (40 μM) in aerated DMSO at 293 K. Excitation occurred with a 532 nm cw-laser at the maximal power density of 144.4 W cm^{-2}.

Upconversion lifetime

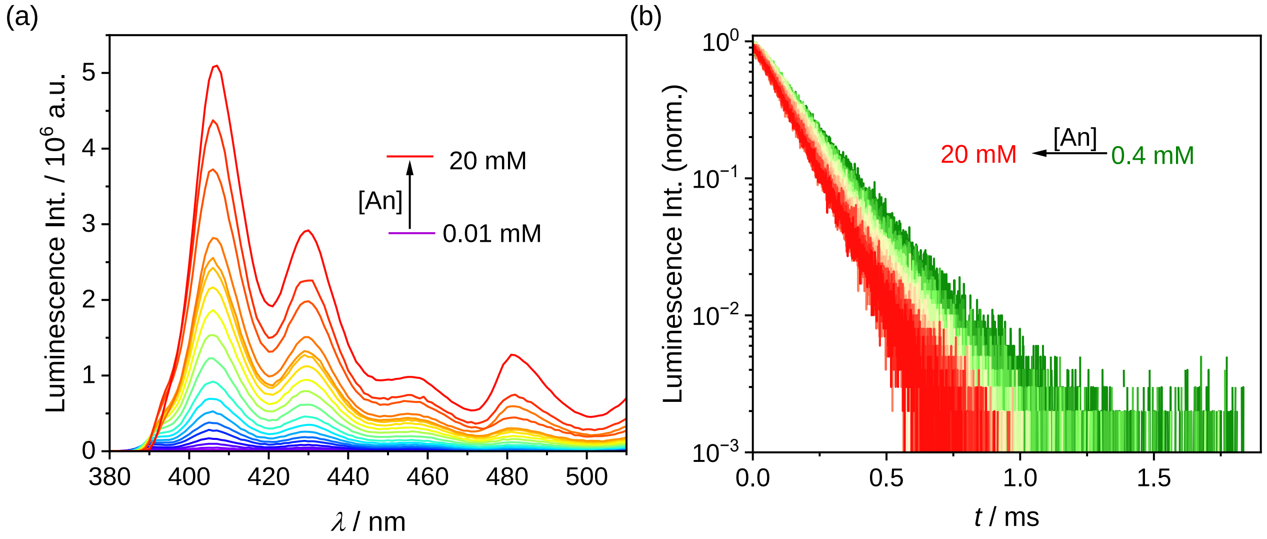

Figure S36. a) Upconversion luminescence spectra and b) normalized upconversion luminescence decays at 407 nm recorded from a solution containing [Fe(phtmeimb)$_2$]PF$_6$ (40 μM) with different concentrations of An (0.4 mM – 20 mM) in aerated DMSO at 293 K. Excitation occurred with a 532 nm cw-laser at 200 mW, a 495 nm long pass filter was placed between the laser and the sample. For the upconversion lifetime measurements, the 532 nm cw-laser was pulsed with a pulse width of 250 μs.

An increase in the annihilator concentration leads to enhanced upconversion luminescence, whereas the upconversion lifetime becomes shorter. At higher concentrations of annihilator, the encounter-based TTA process occurs more frequently. This leads to a faster decay of the T_1-excited state of the annihilator, which corresponds to a shorter upconversion lifetime due to their correlation mentioned above,[38-40] in line with our observations (Figure S36b).

8.4 Fe(III) + PhAn

Excitation power density dependence

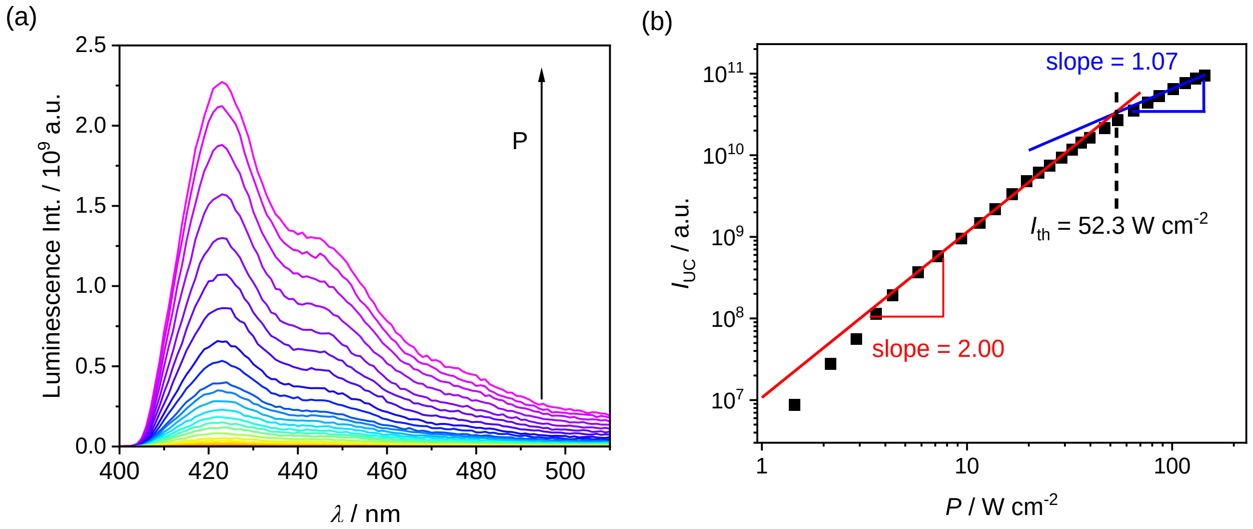

Figure S37. a) Upconversion luminescence intensity of $[Fe(phtmeimb)_2]PF_6$ (40 µM)/PhAn (20 mM) in aerated DMSO excited with a green 532 nm cw-laser at different laser powers (2 mW to 200 mW). A 495 nm long pass filter was placed between the laser and the sample. b) Excitation power density dependence of the upconversion luminescence integral from 400 to 510 nm extracted from a) as a log-log plot. Linear fit gives a slope of 2.00 at low excitation power densities and 1.07 at high excitation power densities. A threshold value I_{th} was determined to 52.3 W cm^{-2} at the intersection of the linear fits.

Selective excitation of the $[Fe(phtmeimb)_2]PF_6$ (40 µM)/PhAn (20 mM) pair in aerated DMSO at 532 nm with increasing power density leads to dramatically enhanced upconversion luminescence (Figure S37a). A log-log plots of the upconversion luminescence integral ($I_{400\text{-}510}$) as a function of the excitation power density P gives a slope of 2.00 at low excitation power densities, due to the biphotonic nature of TTA (second-order reaction). At high excitation power densities, the dependence becomes linear (slope of 1.07), indicating an upconversion saturation. A threshold value I_{th} at the intersection of the linear fits is found at 52.3 W cm^{-2}. This is higher than the I_{th} values of green-to-blue sTTA-UC systems sensitized by other ^{3}d metal complexes based on Zn(II),[43, 44] Cu(I),[45] Cr(III),[46] which are usually below 2 W cm^{-2} in solution, presumably owing to their µs-scaled excited state lifetimes.[40, 43]

Upconversion quantum yields

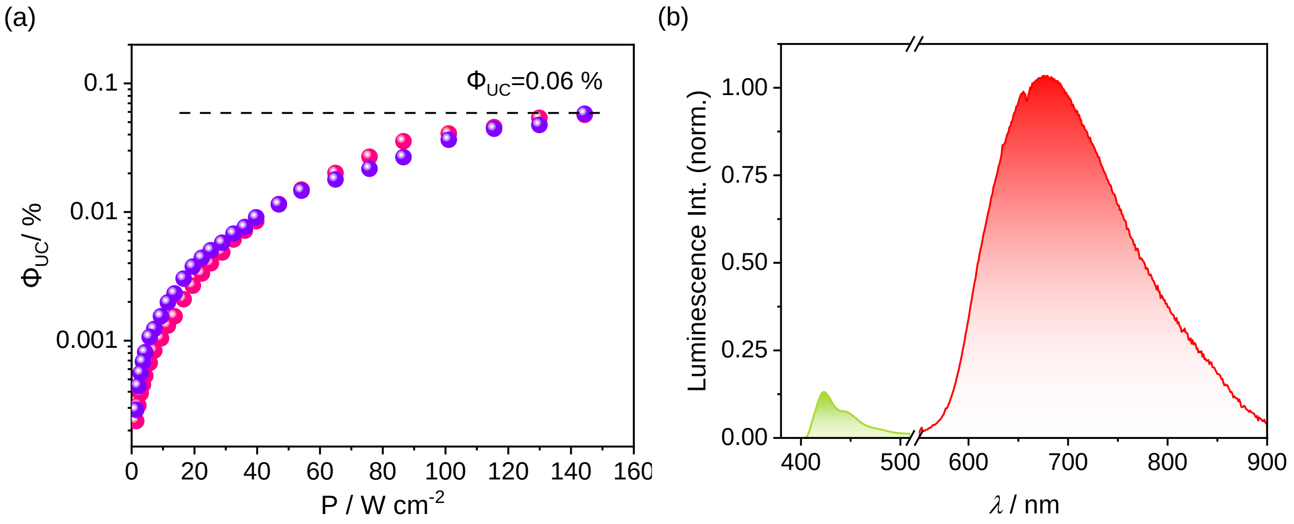

Figure S38. a) Upconversion luminescence quantum yield (Φ_{UC}) of [Fe(phtmeimb)$_2$]PF$_6$ (40 μM)/PhAn (20 mM) in aerated DMSO at 293 K as a function of the excitation power density (532 nm cw-laser). A 495 nm long pass filter was placed between the laser and the sample. These measurements were carried out independently for twice on different days. A maximal upconversion quantum yield Φ_{UC} of 0.06% was obtained for the [Fe(phtmeimb)$_2$]PF$_6$ (40 μM) /PhAn (20 mM) pair under our conditions. b) Upconversion luminescence spectrum (light green) of the [Fe(phtmeimb)$_2$]PF$_6$ (40 μM)/PhAn (20 mM) pair and the prompt luminescence spectrum (red) of the [Fe(phtmeimb)$_2$]PF$_6$ (40 μM) in aerated DMSO at 293 K. Excitation occurred with a 532 nm cw-laser at the maximal power density of 144.4 W cm^{-2}.

Upconversion lifetime

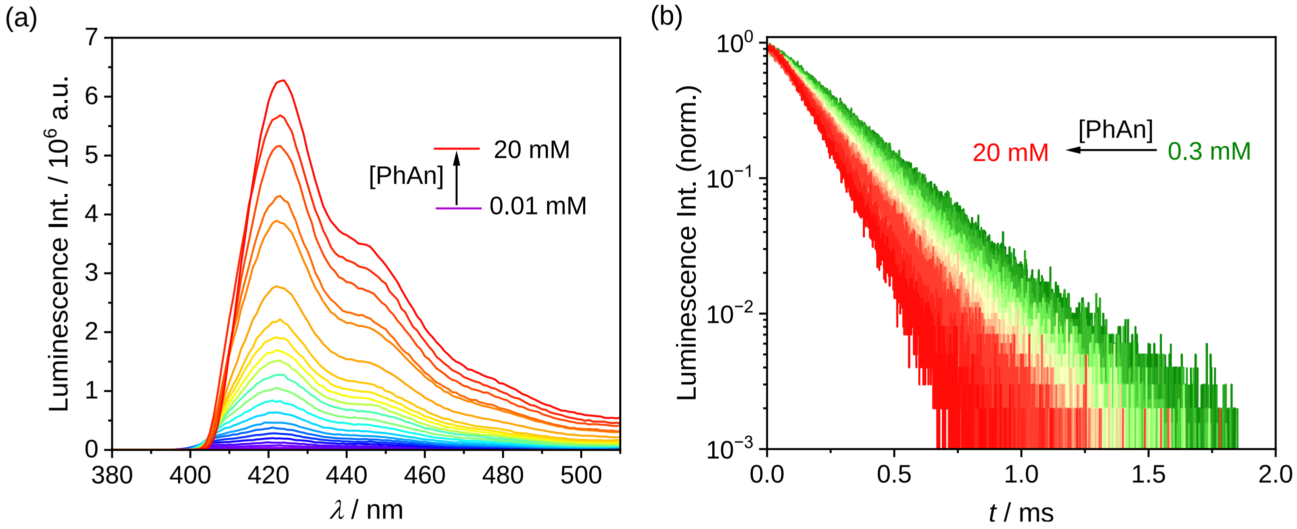

Figure S39. a) Upconversion luminescence spectra and b) normalized upconversion luminescence decay at 420 nm recorded from a solution containing [Fe(phtmeimb)$_2$]PF$_6$ (40 μM) with different concentrations of PhAn (0.3 mM – 20 mM) in aerated DMSO at 293 K. Excitation occurred with a cw-laser at 200 mW, a 495 nm long pass filter was placed between the laser and the sample. For the upconversion lifetime measurements, the 532 nm cw-laser was pulsed to a pulse width of 250 μs.

For the [Fe(phtmeimb)$_2$]PF$_6$/PhAn pair, an increase in the annihilator concentration leads to enhanced upconversion luminescence, whereas the upconversion lifetime becomes shorter, as observed and discussed with the [Fe(phtmeimb)$_2$]PF$_6$/An pair (section 8.3).

8.5 Fe(III) + DPA

Excitation power density dependence

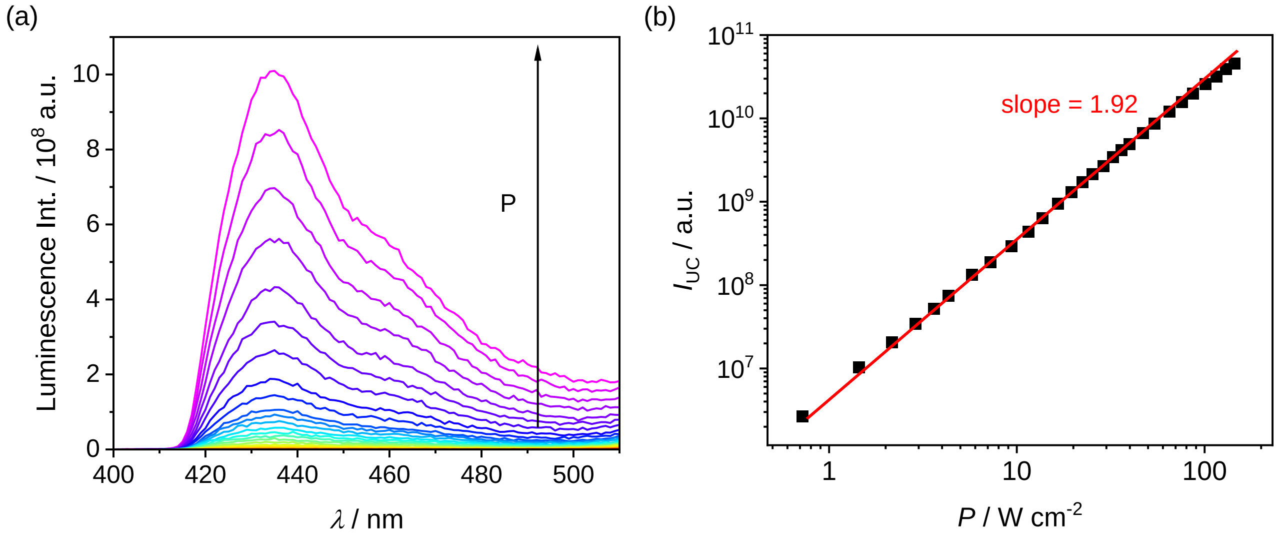

Figure S40. a) Upconversion luminescence intensity of $[Fe(phtmeimb)_2]PF_6$ (40 μM) /DPA (10 mM) in aerated DMSO excited with a green 532 nm cw-laser at different laser powers (1 mW to 200 mW). A 495 nm long pass filter was placed between the laser and the sample. b) Excitation power density dependence of the upconversion luminescence integral from 410 to 510 nm extracted from a) as a log-log plot. Linear fit of the plot gives a slope of 1.92, indicating a biphotonic nature of the upconversion. For the $[Fe(phtmeimb)_2]PF_6$ (40 μM)/DPA (10 mM) pair, no upconversion saturation is observed, presumably due to the very short ^{2}LMCT excited state lifetime of the sensitizer.[40, 43]

Upconversion quantum yields

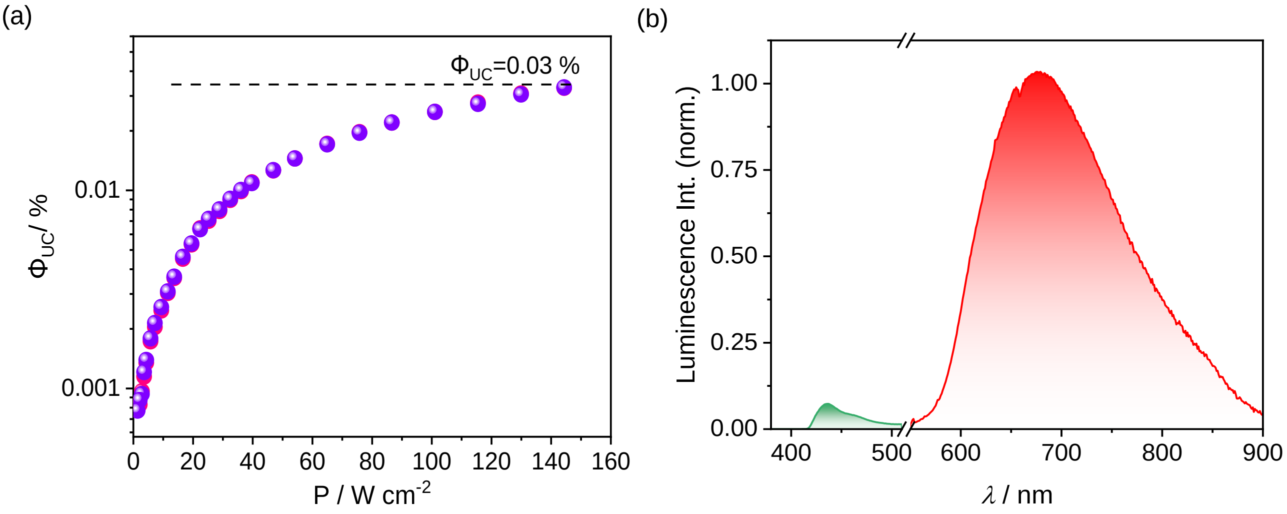

Figure S41. a) Upconversion luminescence quantum yield(Φ_{UC}) of [Fe(phtmeimb)$_2$]PF$_6$ (40 μM)/DPA (10 mM) in aerated DMSO at 293 K as a function of the excitation power density (532 nm cw-laser). A 495 nm long pass filter was placed between the laser and the sample. These measurements were carried out independently for twice on different days. A maximal upconversion quantum yield Φ_{UC} of 0.03% was obtained for the [Fe(phtmeimb)$_2$]PF$_6$ (40 μM) /DPA (10 mM) pair under our conditions. b) Upconversion luminescence spectrum of the [Fe(phtmeimb)$_2$]PF$_6$ (40 μM)/DPA (10 mM) pair (green) and the prompt luminescence spectrum (red) of the [Fe(phtmeimb)$_2$]PF$_6$ (40 μM) in aerated DMSO at 293 K. Excitation occurred with a 532 nm cw-laser at the maximal power density of 144.4 W cm^{-2}.

Upconversion lifetime

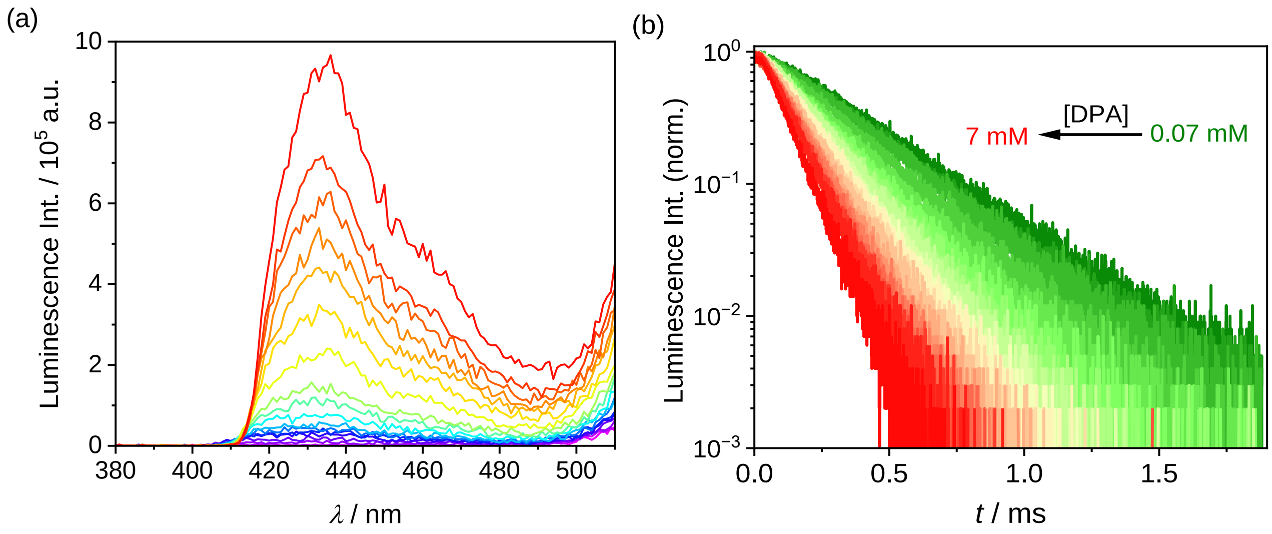

Figure S42. a) Upconversion luminescence spectra and b) normalized upconversion luminescence decay at 430 nm recorded from a solution containing [Fe(phtmeimb)$_2$]PF$_6$ (40 μM) with different concentrations of DPA (0.07 mM – 7 mM) in aerated DMSO at 293 K. Excitation occurred with a cw-laser at 200 mW, a 495 nm long pass filter was placed between the laser and the sample. For the upconversion lifetime measurements, the 532 nm cw-laser was pulsed with a pulse width of 250 μs.

For the [Fe(phtmeimb)$_2$]PF$_6$/DPA pair, an increase in the annihilator concentration leads to enhanced upconversion luminescence, whereas the upconversion lifetime becomes shorter, as observed with the [Fe(phtmeimb)$_2$]PF$_6$/An and [Fe(phtmeimb)$_2$]PF$_6$/PhAn pairs due to the enhanced TTA process (section 8.3 and 8.4).

Table S2. Photon upconversion parameters of the $[Fe(phtmeimb)_2]PF_6$/anthracenes pairs in the presence and absence of mediator. Excitation occurred at 532 nm with a cw-laser.

	Mediator	λ_{em} ^{a}/nm NM	ΔE/eV	Φ_{UC} ^{b}/%	τ_{UC} ^{c}/μs	k_q / M^{-1} s^{-1}	Φ_{DTET} ^{d} /%
Fe(III)/An	none	407	-0.72	0.003	109	1.13×10^{10}	12.33
Fe(III)/PhAn	none	420	-0.62	0.06	119	7.04×10^{10}	14.47
	An			0.04	102	-	-
Fe(III)/DPA	none	435	-0.52	0.03	110	1.82×10^{11}	22.49 (22.92)
	An			0.19	108	-	-
	PhAn			0.16	97	-	-

a: The wavelength of upconversion luminescence maximum, b: Upconversion luminescence quantum yield (Φ_{UC}) of $[Fe(phtmeimb)_2]PF_6$ (40 μM)/annihilators (An (20 mM), PhAn (20 mM) or DPA (10 mM)) in the presence and absence of a mediator (10 mM) in aerated DMSO at 293 K. Excitation occurred with a 532 nm cw-laser at the maximal power density of 144.4 W cm^{-2}. A 495 nm long pass filter was placed between the laser and the sample. c: Upconversion luminescence decays of the same samples used in Φ_{UC} measurement at λ_{em} in deaerated DMSO at 293 K. d: The DTET efficiency derived from the luminescence intensity-based quenching at the given quencher concentrations (section 5.1- 5.3). The value in parentheses is calculated from the excited state lifetime quenching (section 7.1).

The maximal upconversion quantum yields Φ_{UC} for the Fe(III)/anthracenes pairs ranges from 0.003% for An to 0.06% for PhAn under our conditions (Table S2). For the Fe(III)/An pair, photodimerization of An via upconversion and the intrinsic low fluorescence quantum yield of An can diminish the observed upconversion quantum yield.[4, 47] For the $[Fe(phtmeimb)_2]PF_6$/DPA pair, the Φ_{UC} of 0.03% is lower than that obtained with PhAn, likely due to the solubility-limited lower concentration of DPA in DMSO than the other annihilators. These low Φ_{UC} values are in general attributed to the short ^{2}LMCT excited state lifetime of the Fe(III)-based photosensitizer and the following low DTET efficiencies (Table S2).

9. sTTA-UC with mediator

9.1 Fe(III) + PhAn with An as the mediator

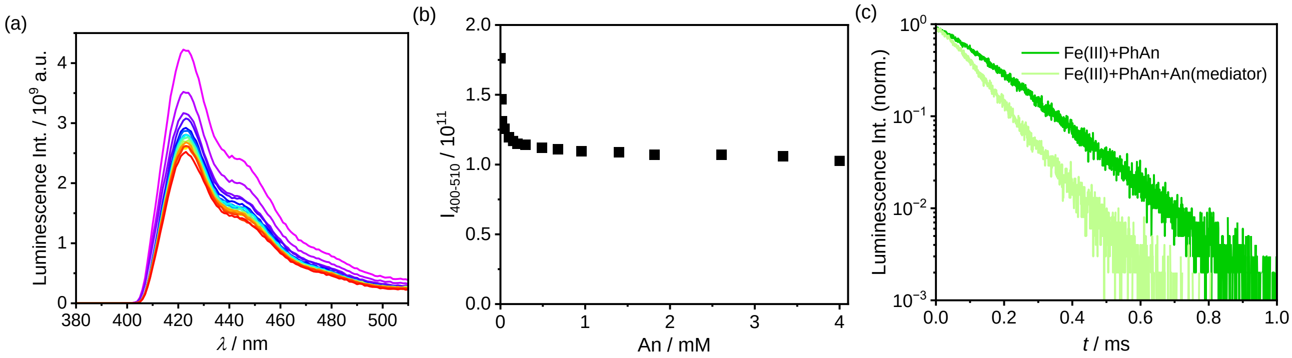

Figure S43. a) Upconversion luminescence spectra of $[Fe(phtmeimb)_2]PF_6$ (40 µM) /PhAn (20 mM) in the presence of An as the mediator with different concentrations (0 – 4 mM) in aerated DMSO at 293 K. Excitation occurred with a 532 nm cw-laser at the maximal power (200 mW). b) Upconversion luminescence integral ($I_{400\text{-}510}$) as a function of the concentration of An mediator extracted from a). c) Upconversion luminescence decays of $[Fe(phtmeimb)_2]PF_6$ (40 µM)/PhAn (20 mM) at 420 nm in the absence of An (green solid trace) and in the presence of 10 mM An (bright green solid trace).

Upconversion performance

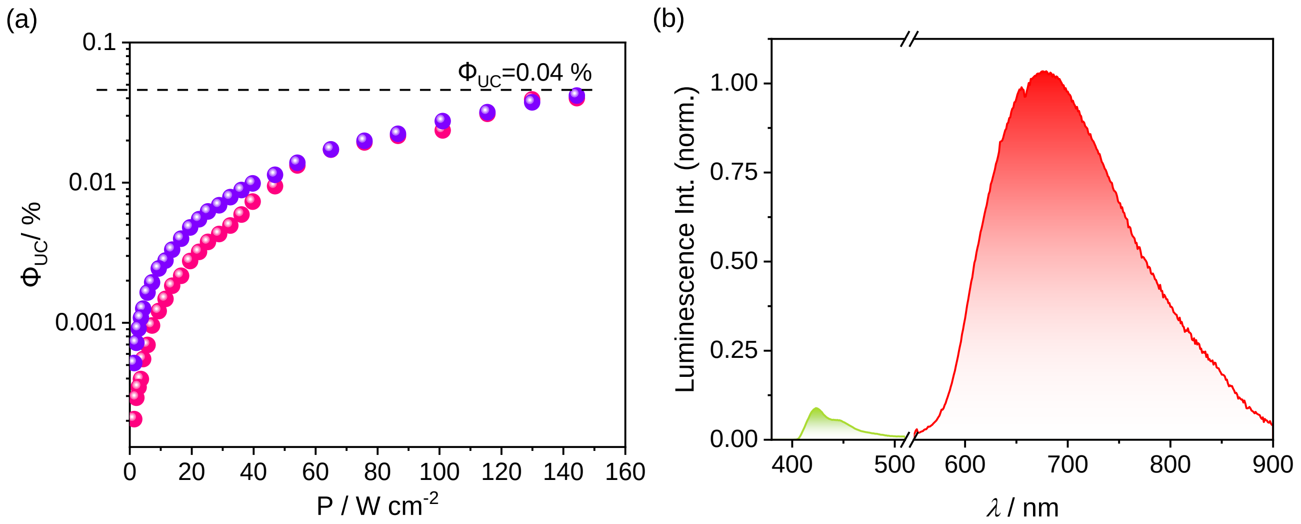

Figure S 44. a) Upconversion luminescence quantum yield (Φ_{UC}) of [Fe(phtmeimb)$_2$]PF$_6$ (40 μM) / PhAn (20 mM) in the presence of An (10 mM) as the mediator in aerated DMSO at 293 K as a function of the excitation power density (532 nm cw-laser). A 495 nm long pass filter was placed between the laser and the sample. These measurements were carried out independently for twice on different days. A maximal upconversion quantum yield Φ_{UC} of 0.04% was obtained for the [Fe(phtmeimb)$_2$]PF$_6$ (40 μM)/PhAn (20 mM) pair in the presence of 10 mM An. b) Upconversion luminescence spectrum (light green) of the [Fe(phtmeimb)$_2$]PF$_6$ (40 μM)/PhAn (20 mM) pair in the presence of An(10 mM) and the prompt luminescence spectrum (red) of the [Fe(phtmeimb)$_2$]PF$_6$ (40 μM) in aerated DMSO at 293 K. Excitation occurred with a 532 nm cw-laser at the maximal power density of 144.36 W cm^{-2}. No fluorescence feature of An is detected in the upconversion luminescence spectra under these conditions.

For the [Fe(phtmeimb)$_2$]PF$_6$/PhAn pair, the upconversion luminescence from PhAn decreases upon addition of An as the mediator (Figure S43 a and b). This is attributed to the reverse TTET (rTTET) from the PhAn to An due to their small energy gap (0.04 eV), which is seen as a further deactivation pathway of the T$_1$-excited state of PhAn (Figure S28). This is further reflected by the discrepancy in the faster upconversion luminescence decay (Figure S43c) and the slower transient absorption decay (Figure S27c) of the T$_1$-excited state of PhAn in the presence of An. The upconversion quantum yield Φ_{UC} drops from 0.06% to 0.04% upon the addition of An (Figure S38a and 44a). Evidently, the formation of the “triplet reservoir” between the T$_1$ states of the mediator and the annihilator diminishes the upconversion performance. For the upconversion luminescence spectra, no spectral features from An fluorescence is observed under these conditions.

9.2 Fe(III) + DPA with An as the mediator

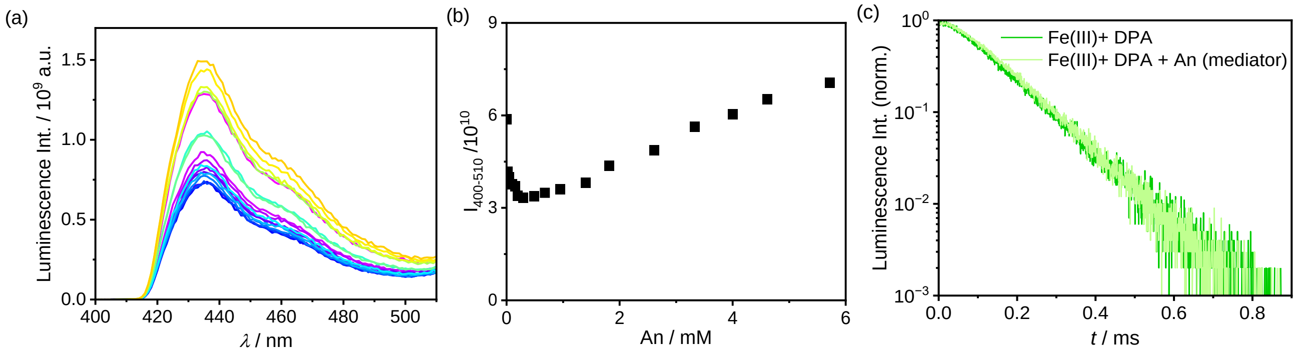

Figure S45. a) Upconversion luminescence spectra of $[Fe(phtmeimb)_2]PF_6$ (40 µM) /DPA (10 mM) in the presence of An as the mediator with different concentrations (0 – 5.71 mM) in aerated DMSO at 293 K. Excitation occurred with a 532 nm cw-laser at the maximal power (200 mW). b) Upconversion luminescence integral ($I_{410\text{-}510}$) as a function of the concentration of An mediator extracted from a). c) Upconversion luminescence decays of $[Fe(phtmeimb)_2]PF_6$ (40 µM)/DPA (10 mM) at 430 nm in the absence of An (green solid trace) and in the presence of 10 mM An (bright green solid trace).

Upconversion performance

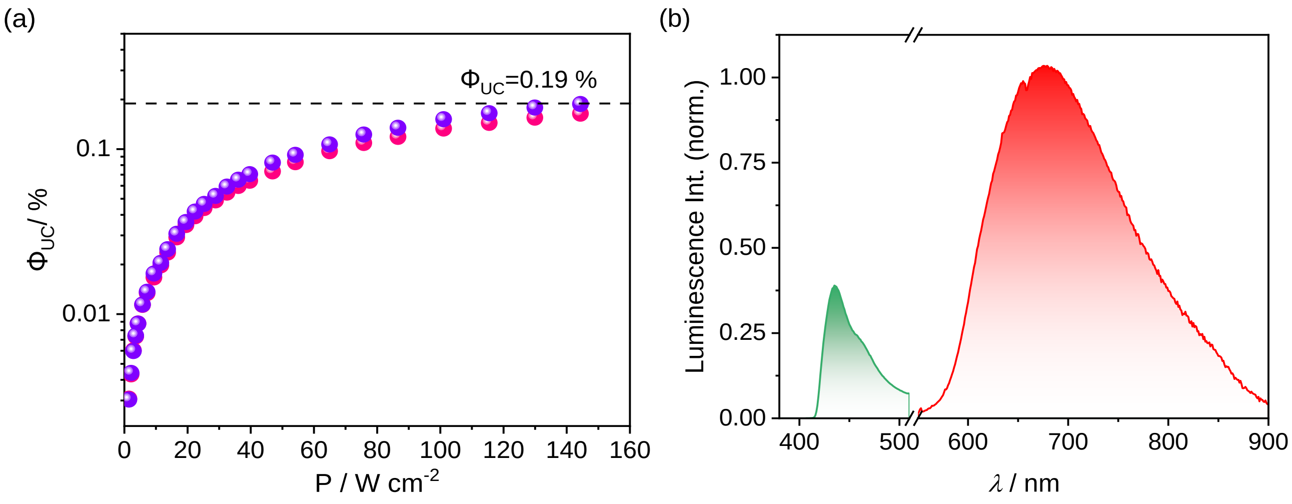

Figure S46. a) Upconversion luminescence quantum yield (Φ_{UC}) of [Fe(phtmeimb)$_2$]PF_6 (40 µM)/DPA (10 mM) in the presence of An (10 mM) as the mediator in aerated DMSO at 293 K as a function of the excitation power density (532 nm cw-laser). A 495 nm long pass filter was placed between the laser and the sample. These measurements were carried out independently for twice on different days. A maximal upconversion quantum yield Φ_{UC} of 0.19% was obtained for the [Fe(phtmeimb)$_2$]PF_6 (40 µM)/DPA (10 mM) pair in the presence of 10 mM An. b) Upconversion luminescence spectrum (green) of the [Fe(phtmeimb)$_2$]PF_6 (40 µM) / DPA (10 mM) pair in the presence of An (10 mM) and the prompt luminescence spectrum (red) of the [Fe(phtmeimb)$_2$]PF_6 (40 µM) in aerated DMSO at 293 K. Excitation occurred with a 532 nm cw-laser at the maximal power density of 144.4 W cm^{-2}. No fluorescence feature of An is detected in the upconversion luminescence spectra under these conditions.

For the [Fe(phtmeimb)$_2$]PF_6/DPA pair, an increase in the concentration of the An mediator leads initially to a slight drop of the upconversion luminescence, which starts to increase with An concentration above 0.3 mM (Figure S45a and b). The initial drop in upconversion luminescence is likely assigned to the competing DTET from the Fe(III) complex to the T_1-state of An (section 5.1). At high An concentration (above 0.3 mM), this phenomenon is gradually compensated by the longer T_1-state lifetime of An than the ^{2}LMCT excited state lifetime of the Fe(III) complex, which allows more efficient encounters for TTET with DPA. Consequently, the upconversion luminescence quantum yield Φ_{UC} is improved to 0.19% in the presence of 10 mM An (Figure S41a and 46a), which exceeds the Φ_{UC} value obtained without the mediator by a factor of 6.3. Apparently, the energy level of the T_1-state of mediator plays an essential role in affecting the upconversion performance, and this should not be too close to that of the annihilator to avoid unwanted reserve energy transfer. No fluorescence features of An were observed in the upconversion luminescence spectra and decay kinetics under these conditions.

9.3 Fe(III) + DPA with PhAn as the mediator

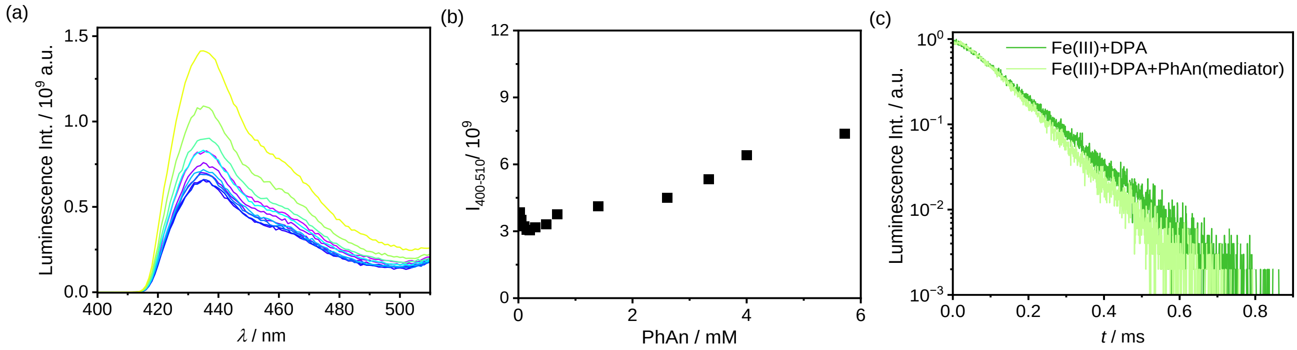

Figure S47. a) Upconversion luminescence spectra of $[Fe(phtmeimb)_2]PF_6$ (40 μM)/DPA (10 pmM) in the presence of PhAn as the mediator with different concentrations (0 – 5.71 mM) in aerated DMSO at 293 K. Excitation occurred with a 532 nm cw-laser at the maximal power (200 mW). b) Upconversion luminescence integral ($I_{410-510}$) as a function of the concentration of PhAn mediator extracted from a). c) Upconversion luminescence decays of $[Fe(phtmeimb)_2]PF_6$(40 μM)/DPA (10 mM) at 430 nm in the absence of PhAn (green solid trace) and in the presence of 10 mM PhAn (bright green solid trace).

Upconversion performance

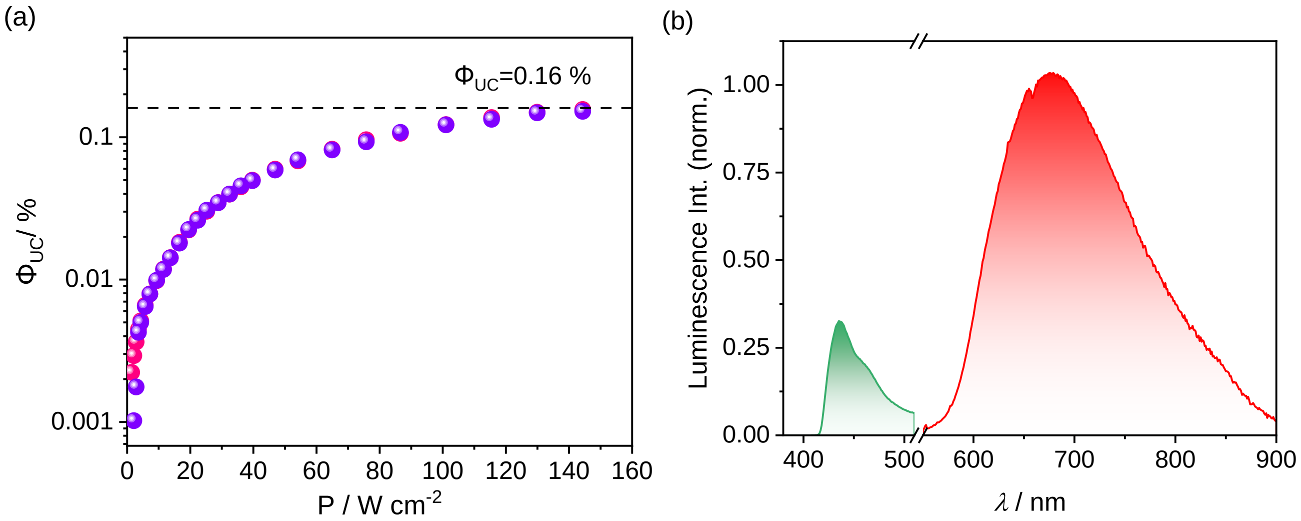

Figure S 48. a) Upconversion luminescence quantum yield (Φ_{UC}) of [Fe(phtmeimb)$_2$]PF$_6$ (40 µM) / DPA (20 mM) in the presence of PhAn (10 mM) as the mediator in aerated DMSO at 293 K as a function of the excitation power density (532 nm cw-laser). A 495 nm long pass filter was placed between the laser and the sample. These measurements were carried out independently for twice on different days. A maximal upconversion quantum yield Φ_{UC} of 0.16% was obtained for the [Fe(phtmeimb)$_2$]PF$_6$ (40 µM)/DPA (10 mM) pair in the presence of 10 mM PhAn. b) Upconversion luminescence spectrum (green) of the [Fe(phtmeimb)$_2$]PF$_6$ (40 µM)/DPA (10 mM) pair in the presence of PhAn(10 mM) and the prompt luminescence spectrum (red) of the [Fe(phtmeimb)$_2$]PF$_6$ (40 µM) in aerated DMSO at 293 K. Excitation occurred with a 532 nm cw-laser at the maximal power density of 144.4 W cm^{-2}.

For the [Fe(phtmeimb)$_2$]PF$_6$/DPA pair, addition of PhAn as the mediator leads to comparable upconversion luminescence enhancement as observed with An (Figure S45 and 46, section 9.2). No fluorescence features of PhAn was observed in the upconversion luminescence spectra under these conditions.

10. Photostability

10.1 Fe(III) + DPA

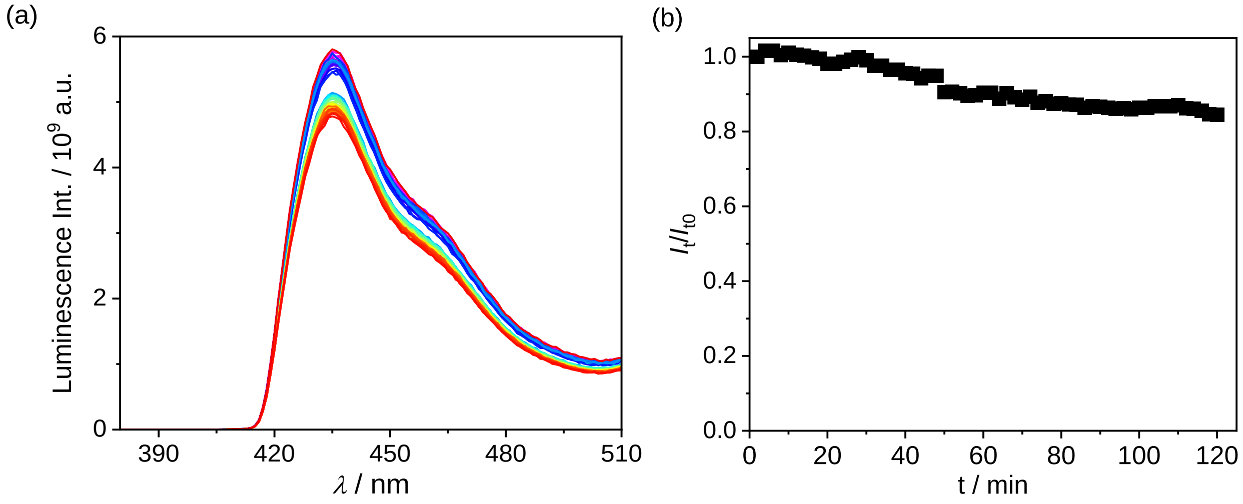

Figure S49. Photostability study of the upconversion pair [Fe(phtmeimb)$_2$]PF$_6$ (40 µM) /DPA (10 mM) in aerated DMSO at 20 °C. a) Upconversion luminescence spectra of the upconversion sample recorded under continuous irradiation with a 532 nm cw laser (~ 144 W cm^{-2}) for two hours. b) Relative upconversion luminescence intensity I/I$_0$ as a function of irradiation time, where I$_0$ is the initial upconversion luminescence intensity prior to laser irradiation. After two hours of laser irradiation, the upconversion luminescence remains at 85% of the initial intensity, indicating high photostability of the upconversion system under the applied conditions.

10.2 Fe(III) + DPA with An as the mediator

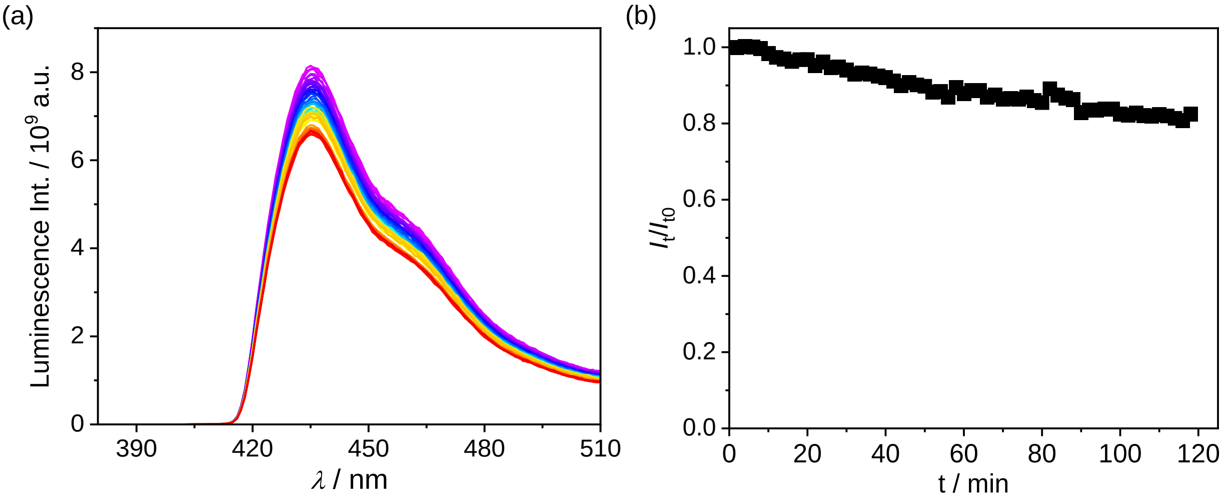

Figure S50. Photostability study of the upconversion pair $[Fe(phtmeimb)_2]PF_6$ (40 µM) /DPA (10 mM) in the presence of An as the mediator in aerated DMSO at 20 °C. a) Upconversion emission spectra of the upconversion sample recorded under the 532 nm cw laser (200 mW, ~ 144 W cm^{-2}) irradiation over 2 hours. b) Relative emission intensity I/I_0 as a function of irradiation time, where I_0 is the initial emission intensity prior to laser irradiation. After two hours of laser irradiation, the upconversion signal remains at 85% of the initial emission intensity, indicating high photostability of the upconversion system under the applied conditions.

11. Photocatalytic polymerizations

11.1 Reaction mechanism

Photopolymerization is an essential method to produce polymer materials,[48, 49] especially the acrylate polymers, which are widely used in industry and popular for biomedicines, due to their light transparency, elasticity, weatherability, and high biocompatibility.[50-52] Photocatalytic radical polymerization allows the synthesis of these polymers in a spatially and temporally controlled manner by light,[40, 53-56] and using low-energetic visible light to drive these reactions is particularly attractive for 3D printing.[57-59]

DPA is a well-known blue emitter for sTTA-UC,[60, 61] but the utilization as a photoredox catalyst remains underexplored, and the extreme few examples require irradiation with high-energy photons at ~ 395 nm.[62, 63] Particularly, DPA radical anion has an strong reducing power $E^0_{1/2}$ (DPA/DPA$^{\bullet -}$) = -1.94 V vs SCE in the ground state,[13] which enables the reduction of an alkyl halide-based initiator, such as ethyl 2-bromopropionate (EBP) as shown in Figure S51.[64, 65]

According our previous finding, the long excited state lifetime can boost the excited state reactivity for electron- or energy transfer with small driving forces.[66] It is in principle feasible to use long-lived photoactive ^{1}DPA* generated by sTTA upconversion to initiate photopolymerization. We decide to use our Fe(III)/DPA upconversion pair for green-light driving photocatalytic radical polymerization of acrylate monomers, including trimethylolpropane triacrylate (TMPTA, section 11.2) and poly(ethylenglycol)diacrylate (PEGDA, section 11.3).

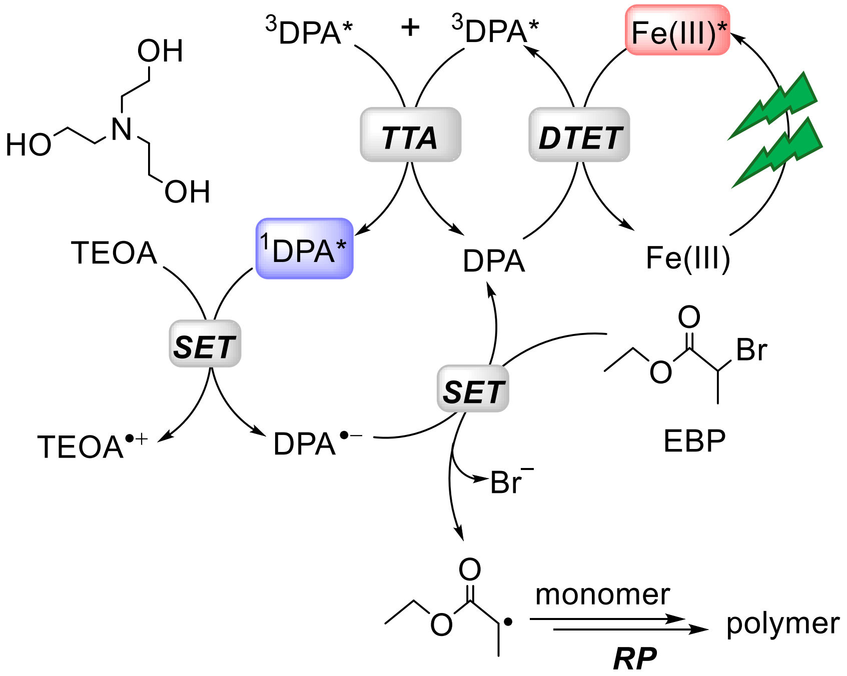

Figure S51. The feasible photopolymerization mechanism catalyzed by [Fe(phtmeimb)$_2$]PF$_6$ (70 μM)/DPA (7 mM) with green light irradiation and the molecular structure of TEOA and EBP. DTET: doublet-triplet energy transfer; TTA: triplet-triplet annihilation; SET: single electron transfer; RP: radical polymerization.

In photoredox catalysis, the reduction potential of an electronically excited chromophore (E^0(*A/ A$^{\bullet-}$)) is usually estimated by adding the excited state energy (E^{00}, in units of eV, divided by the elementary charge *e*) to the ground-state reduction potential (E^0(A/A$^{\bullet-}$)) using the following equation: [67]

$$E^0(^*A/A^{\bullet-}) = E^0(A/A^{\bullet-}) + E^{00}/e \qquad \text{(Eq. S4)}$$

DPA has a halfwave reduction potential $E^0_{1/2}$ (DPA/DPA$^{\bullet-}$) = – 1.94 V vs SCE in the ground states and the singlet excited state (S_1) of 3.00 eV (the first fluorescence emission band at 413 nm, Figure S6a).[13] Therefore, the excited state reduction potential is calculated to be

E^0(*A/A$^{\bullet-}$) = 1.06 V vs SCE. Triethanolamine (TEOA) is an amine-based electron donor with a halfwave redox potential of E^0(D$^+$/D) = 0.90 V vs SCE.[13] A driving force of ΔG_{ET} = – 0.16 eV for single electron transfer (SET) from the singlet excited state of the DPA to TEOA is estimated according to Eq.S5.[68]

$$\Delta G_{ET} = e \times [E^0_{1/2}\ (TEOA^{\bullet+}/TEOA) - E^0(^1DPA^*/DPA^{\bullet-})] \qquad (Eq.\ S5)$$

Therefore, electron transfer from the singlet excited state of DPA (^{1}DPA*) to TEOA is an exothermic process. Figure S51 illustrates the plausible reaction mechanism for photocatalytic polymerization process using the Fe(III)/DPA upconversion pair and green light irradiation. In an aerated DMSO solution containing the acrylate monomer TMPTA or PEGDA, EBP as the initiator, TEOA as the additive, and the Fe(III)/DPA pair, irradiation with a 532 nm green laser (200 mW) forms the ^{1}DPA* in the laser beam via sTTA-UC. Therefore, formation of the DPA radical anion (DPA$^{\bullet-}$) can be in principle accessed via photoinduced electron transfer from the exothermic reductive quenching by TEOA.[63] The formed DPA$^{\bullet-}$ reduces subsequently the C—Br bond of the EBP initiator, which gives a bromide ion and a propagating alkyl radical for radical polymerization (Figure S51).[64, 65] Meanwhile, DPA is recovered to its ground state and the catalytic circle is closed.

In the presence of TEOA, the ^{2}LMCT excited state of the Fe(III) complex can be principally quenched via electron transfer with an estimated driving force of ΔG_{ET} = – 0.47 eV according to Eq.S6.[10] However, no polymer was formed under the identical conditions when DPA is omitted (section 11.2 and 11.3), likely due to the short excited state lifetime of the Fe(III) complex that kinetically hinders the reactions.[66]

$$\Delta G_{ET} = e \times [\ E^0_{1/2}\ (TEOA^{\bullet+}/TEOA) - E^0(Fe(III)^*/Fe(II))\ ] \qquad (Eq.\ S6)$$

In the presence of An as the mediator, no clear polymer was formed with the Fe(III)/DPA pair under laser irradiation for 5 min (irradiation time used for photopolymerization reactions in section 11.2 and 11.3). This is likely attributed to the photoredox activity of the mediator ^{3}An* that hinders the polymerization reactions. For instance, ^{3}An* has a redox potential of – 0.14 V vs SCE,[13] which can be reductively quenched by DPA$^{\bullet-}$ with a large driving force of ~ – 1.80 eV. This could hinder the reaction of DPA$^{\bullet-}$ with the initiator EBP for polymerization. Although the formed An$^{\bullet-}$ has similar redox potential as DPA$^{\bullet-}$ that can reduce the EBP initiator, the introduction of An seems to hinder the overall reaction rate and efficiency. Indeed, for the Fe(III)/DPA pair with An as the mediator, longer irradiation time up to 30 min was needed to form the polymer, but this reaction is significantly faster in the absence of An. Therefore, the following photopolymerization reactions (section 11.2 and 11.3) are performed without the mediator.

To explore the photoredox catalytic reactivity of ^{1}DPA* for singlet electron transfer with TEOA,

the prompt fluorescence lifetime of DPA was measured with 390 nm excitation in the presence of TEOA of different concentrations. However, no clear quenching in the DPA fluorescence lifetime was observed (Figure S52b). In contrast, selective excitation of the $[Fe(phtmeimb)_2]PF_6$ (70 μM)/DPA (7 mM) pair in aerated DMSO at 532 nm gives the initial upconversion luminescence lifetime of 131 μs (Figure S52a), which is clearly quenched by the addition of TEOA. This gives a quenching rate constant k_q of 3.20×10^6 M^{-1} s^{-1} (Figure 52a, inset), which is three orders of magnitude below the diffusion limit of DMSO ($k_{diff.}$ = 3.3×10^9 M^{-1} s^{-1} at room temperature).[13] Considering the small driving force ΔG_{ET} = – 0.16 eV for electron transfer, this k_q value seems reasonable according to the Rehm-Weller theory.[69, 70] In the presence of 70 mM TEOA (concentration used for polymerization below), the quenching rate v_q is calculated to 2.24×10^5 s^{-1}. This exceeds the inherent decay rate (zero-order reaction) of the upconverted ^{1}DPA* ($v_0 = k_0 = 1 / \tau_{UC} = 7.69 \times 10^3$ s^{-1}) by a factor of 29 and leads to a quenching efficiency of ~ 62% (Figure S52a). For the prompt DPA fluorescence with a lifetime of 1.10 ns in aerated DMSO (Figure S6b), the inherent decay rate of ^{1}DPA* ($v_0 = k_0 = 1 / \tau_0 = 9.09 \times 10^8$ s^{-1}) is more than 4,000 times faster than the above obtained quenching rate v_q. Consequently, fluorescence quenching with the prompt ^{1}DPA* was kinetically hindered.

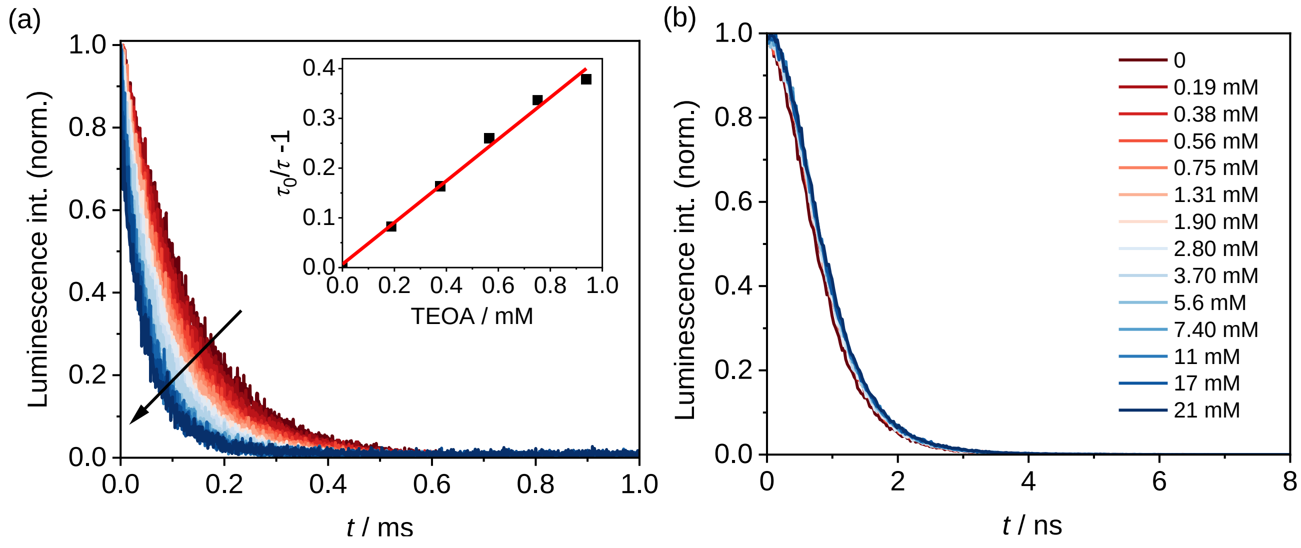

Figure S52. Luminescence lifetime-based Stern-Volmer quenching of DPA fluorescence by TEOA accessed via a) sTTA-UC and via b) direct excitation. a) Normalized upconversion luminescence decays at 430 nm recorded from a solution containing $[Fe(phtmeimb)_2]PF_6$ (40 μM)/DPA(7 mM) with different concentrations of TEOA (0 mM–100 mM) in aerated DMSO at 293 K. Inset: Stern-Volmer plot derived from the quenched luminescence intensity from a) plotted as a function of the TEOA concentration; linear fitting of the data in a) gives the Stern-Volmer constant K_{SV}, and the initial quenching rate constant k_q ($k_q = K_{SV}/\tau_0$, τ_0 = 131 μs from the lifetime obtained in a). Excitation occurred with a 532 nm cw-laser at 200 mW, a 495 nm long pass filter was placed between the laser and the sample. For the upconversion lifetime measurements, the 532 nm cw-laser was pulsed with a pulse width of 250 μs. The luminescence decays were fitted mono exponentially. b) Normalized luminescence decays at 430 nm recorded from a solution containing DPA (10 μM) with different concentrations of TEOA (0 mM – 21 mM) in aerated DMSO at 293 K. Excitation occurred with a pulsed LED at 390 nm.

UC-driving photopolymerization mostly relies a highly emissive upconverted state, which undergoes radiative or non-radiative energy transfer to activate a photoinitiator for polymerization.[40, 57, 58, 71-75] In this study, we develop a new strategy to take the full benefits of photon upconversion, which includes the low-energy visible light for irradiation, the catalytic properties of the singlet excited annihilator for electron transfer, and the long-lived nature of the upconverted excited state for boosted excited state reactivity, to initiate efficient photopolymerization (section 11.2 and 11.3). The proof-of-principle experiment in Figure S51 performs a new strategy for sTTA-UC catalyzed radical polymerization with low-cost iron and low-energy visible light under aerobic conditions, for which only very few examples have been

reported so far, and they rely mainly on photosensitizers based on Pt(II) or Pd(II).[76-78] In comparison to organophotocatalysts used for controlled polymer synthesis via direct excitation,[79-83] the upconversion-driving radical polymerization with the Fe(III)/DPA pair has the following advantages: i) the used Fe(III)-based photosensitizer features visible light absorption band with lower energy than the most organic photoredox catalysts requiring purple or blue light irradiation, and the low-energy visible irradiation light is less harmful and can avoid photodamage as well as inner-filter effects caused by direct excitation with high-energy photons;[84-87] ii) the substantially longer upconversion lifetime (above 100 μs) of DPA sensitized by the Fe(III) complex boosts the excited state reactivity of the catalyst for single-electron transfer (Figure S52) and therefore improves the photocatalytic efficiency. This could be kinetically hindered for many organic photoredox catalysts due to their inherently short excited state lifetimes via direct excitation. Many efforts have been made to replace the heavy metal-based photoredox catalysts with organophotocatalysts for more sustainable polymer synthesis.[82, 88] Iron, as the most abundant transition metal, is an environmentally friendly, non-toxic, and bio-essential element.[89] This makes the use of iron complexes suitable for many applications including biological studies.[90, 91] Additionally, the low amount of the Fe(III)-based photosensitizer up to 50 ppm (section 11.2 and 11.3) makes the removal of the complex in radical polymerization unnecessary. Overall, our study makes fundamentally an important step toward efficient photochemical reactions using abundant iron.

11.2 Trimethylolpropane triacrylate (TMPTA)

Trimethylolpropane triacrylate (TMPTA) is a trifunctional acrylate monomer that is frequently used as polymer building block in industry.[92] For the photopolymerization of TMPTA, the reaction mixture of 1 mL contained 378.9 μl TMPTA (1.41 mmol), 9.18 μL TEAO (70 μmol), 9.08 μL ethyl 2-bromopropionate (EBP, 70 μmol) and 602.84 μL DMSO, in which the upconversion pair[Fe(phtmeimb)$_2$]PF$_6$ (70 μM)/DPA (7 mM) was added. The loading amount of the Fe(III) photosensitizer is 16 ppm relatively to the acrylate groups in the monomer. The control sample was prepared in the absence of either [Fe(phtmeimb)$_2$]PF$_6$ (70 μM), DPA (7 mM), TEOA(70 mM) or light. Irradiation of the samples occurred under ambient conditions with a 532 nm cw-laser (200 mW) in a dark environment.

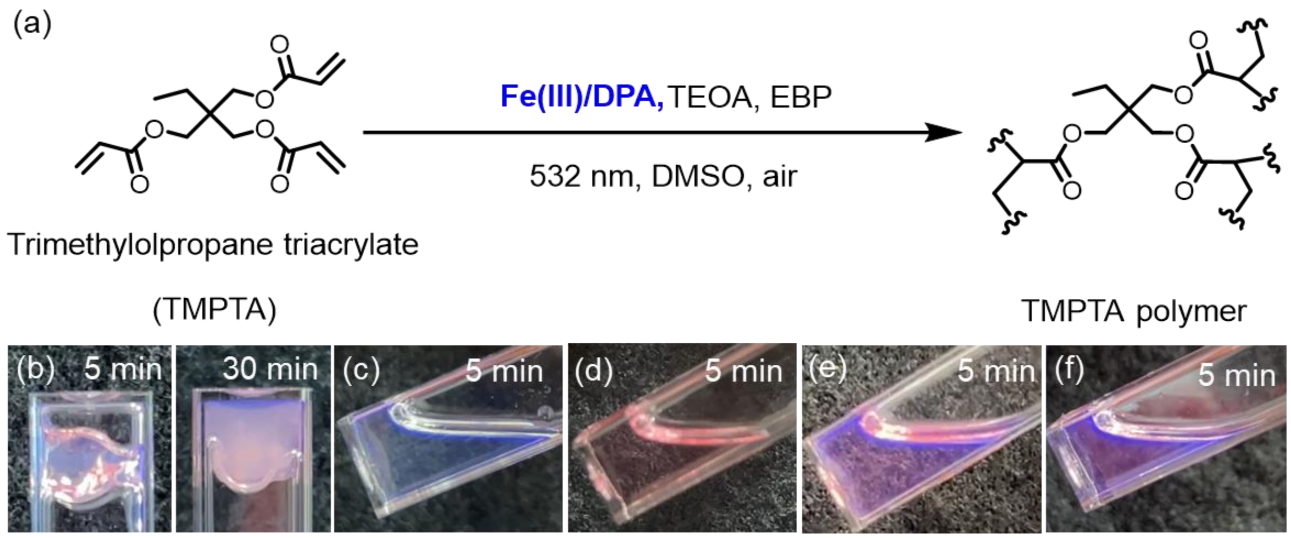

Figure S53. (a) Reaction scheme of photocatalytic polymerization of TMPTA in DMSO with green light under aerobic condition, along with TMPTA polymerization images of b) sample with all the components, sample in the absence of either c) [Fe(phtmeimb)$_2$]PF$_6$, d) DPA, e) TEOA or f) light. The images were recorded after irradiation with a 532 nm cw-laser (200 mW) for the indicated time.

After irradiation of the reaction mixtures containing [Fe(phtmeimb)$_2$]PF$_6$ (70 μM)/DPA (7 mM) in a dark environment for 5 minutes with green light, TMPTA polymerization was initiated and the liquid solutions transformed into a free-standing gel of TMPTA polymer (Figure S53b,left). After 30 min irradiation with green light, more than half of the reaction mixture was converted to a polymer gel (Figure S53b, right). Control experiments, in which either the Fe(III) complex, DPA, TEOA, or light was omitted, no polymerization was observed after 5 min of irradiation (Figure S53c – f).

11.3 Polyethylene glycol diacrylate (PEGDA)

Poly(ethylenglycol)diacrylate (PEGDA) features two terminal acrylate groups on the long polyethylene glycol chain, which is interesting for tissue engineering applications.[93] For the photopolymerization of PEGDA, the reaction mixture of 1 mL contained 440 μL PEGDA (M_n = 700 g / mol, ~ 0.71 mmol), 9.18 μL TEAO (70 μmol), 9.08 μL ethyl 2-bromopropionate (EBP, 70 μmol) and 602.84 μL DMSO, in which the upconversion pair [Fe(phtmeimb)$_2$]PF$_6$ (70 μM)/DPA (7 mM) was added. The loading amount of the Fe(III) photosensitizer is 50 ppm relatively to the acrylate groups in the monomer. The control sample was prepared in the absence of either [Fe(phtmeimb)$_2$]PF$_6$ (70 μM), DPA (7 mM), TEOA(70 mM), or light. Irradiation of the samples occurred under ambient conditions with a 532nm cw-laser (200 mW) in a dark environment.

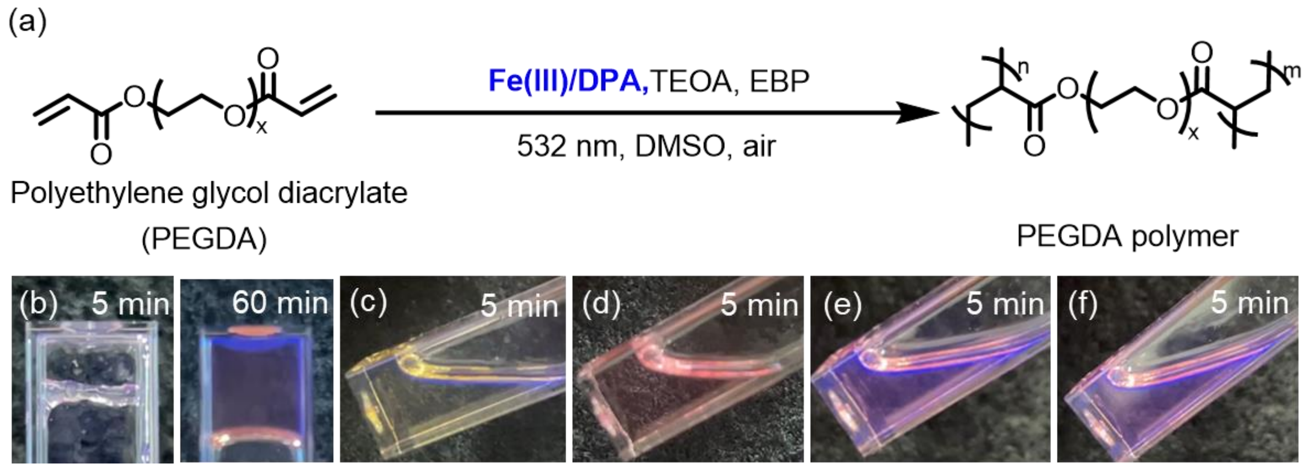

Figure S54. (a) Reaction scheme of photocatalytic polymerization of PEDGA in DMSO with green light under aerobic condition, along with PEGDA polymerization images of b) sample with all the components, sample in the absence of either c) [Fe(phtmeimb)$_2$]PF$_6$, d) DPA, e) TEOA or f) light. The images were recorded with 5 min of irradiation with a 532 nm cw-laser (200 mW). The images were recorded after irradiation with a 532 nm cw-laser (200 mW) for the indicated time.

After irradiation of the reaction mixtures containing [Fe(phtmeimb)$_2$]PF$_6$ (70 μM)/DPA (7 mM) in a dark environment for 5 minutes with green light, PEGDA polymerization was initiated, generating a polymer stick along the light beam (Figure S54b,left). This indicates a spatial control of the polymer synthesis, which is highly attractive stereolithographic 3D printing.[58, 59, 73, 94] After 60 min irradiation with green light, the whole reaction mixture was converted to a free-standing gel (Figure S54b, right). Control experiments, in which either the Fe(III) complex, DPA, TEOA, or light was omitted, no polymerization was observed after 5 min of irradiation (Figure S54c – f).

Driven by the Fe(III)/DPA upconversion pair, photopolymerizations of acrylates monomer (TMPTA and PEGDA) were rapidly initiated within 5 min light irradiation, and a longer irradiation time up to one hour led to a complete conversion of the reaction mixture into a free-standing polymer gel (Figure S54b). Such rapid initiation of visible-light-mediated photopolymerization has been recently achieved with Cu(I)-catalyzed atom-transfer radical polymerizations[95] and Zn(II)-catalyzed radical polymerizations,[76] whereas the most organophotocatalysts typically require multiple hours or days of light irradiation to achieve a high conversion of monomer.[79-83] More quantitative analysis for evaluating the photocatalytic performance of the Fe(III)-sensitized upconversion pair for various polymerization reactions is planned in the near future.

12. NMR data

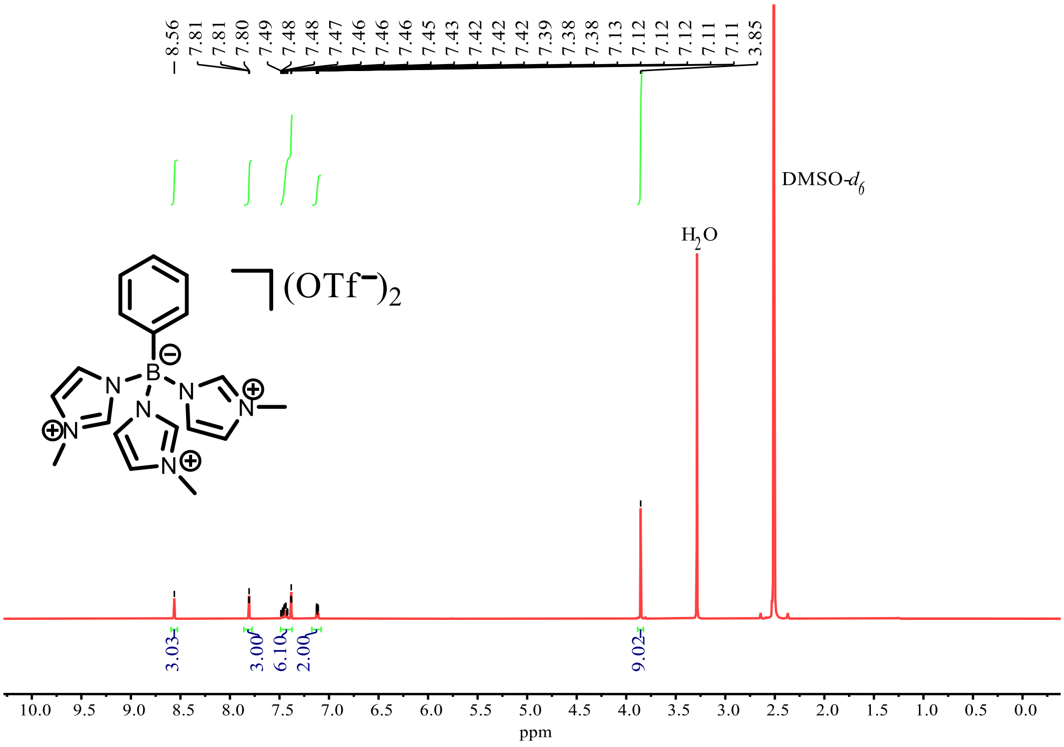

Figure S55. ^{1}H NMR spectrum of L-OTf measured in DMSO-d_6 at 25 °C.

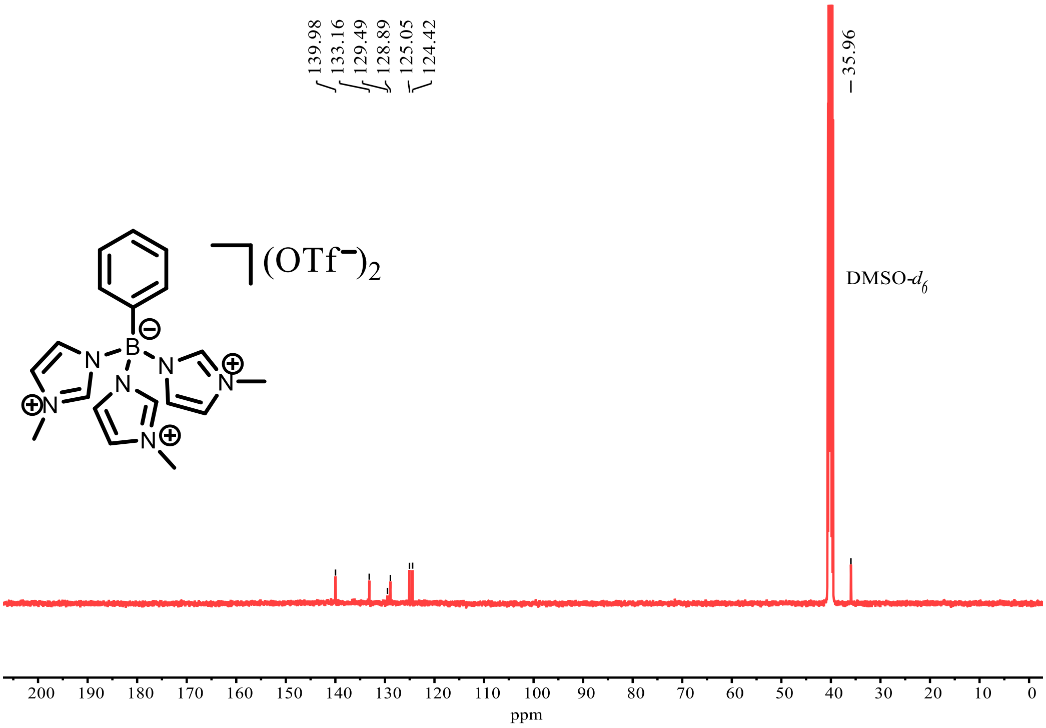

Figure S56. ^{13}C NMR spectrum of L-OTf measured in DMSO-d_6 at 25 °C.

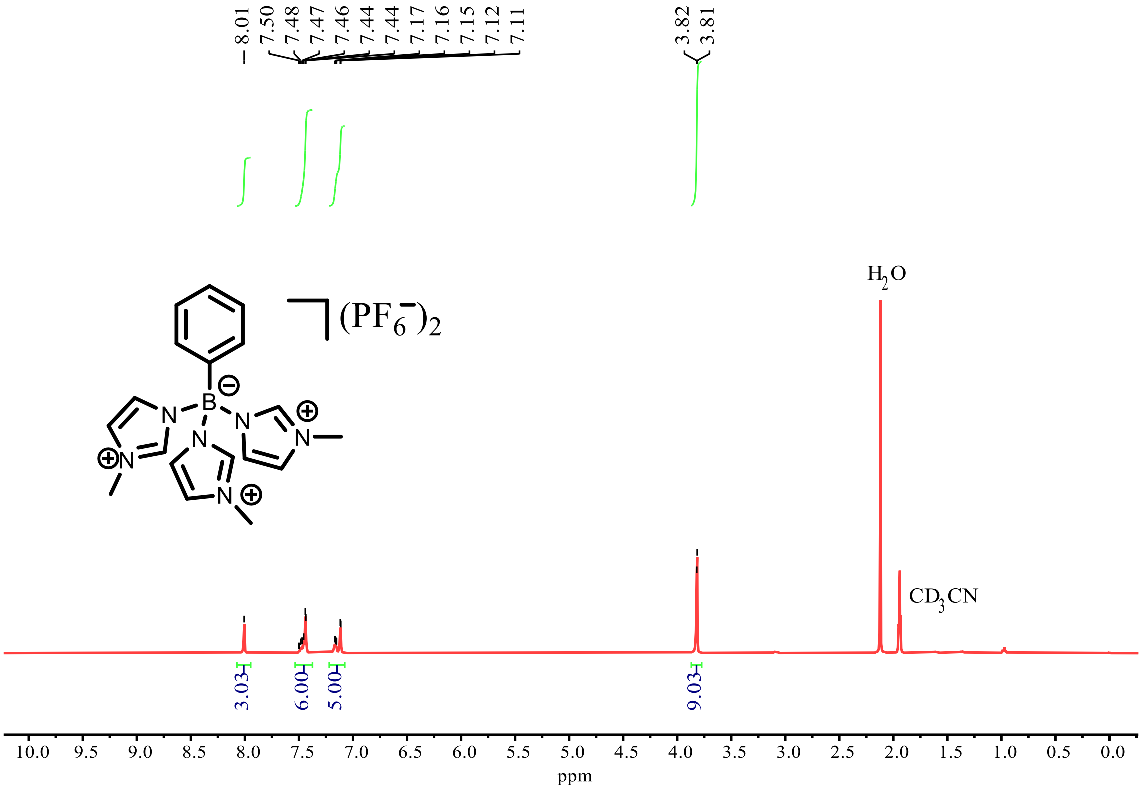

Figure S57. ^{1}H NMR spectrum of L-PF$_6$ measured in acetonitrile-d_3 at 25 °C.

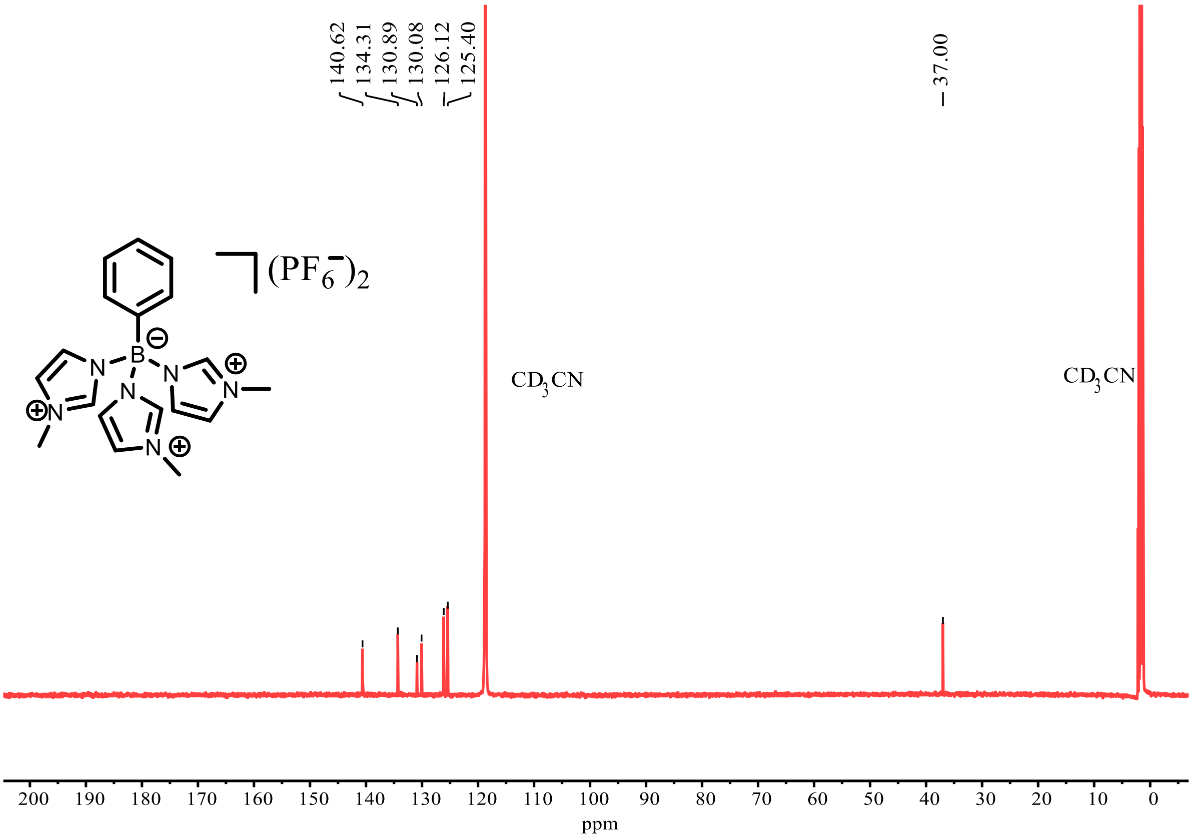

Figure S58. ^{13}C NMR spectrum of L-PF$_6$ measured in acetonitrile-d_3 at 25 °C.

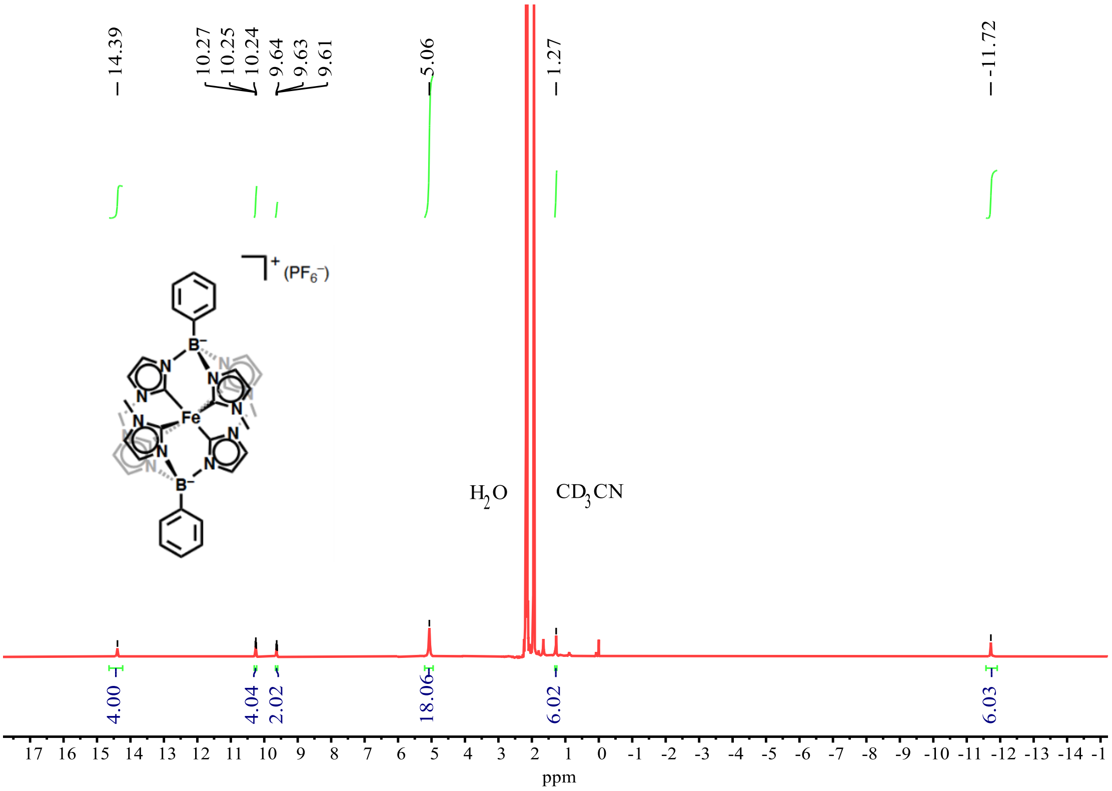

Figure S59. ^{1}H NMR spectrum of [FeIII(phtmeimb)$_2$]PF$_6$ measured in acetonitrile-d_3 at 25 °C.

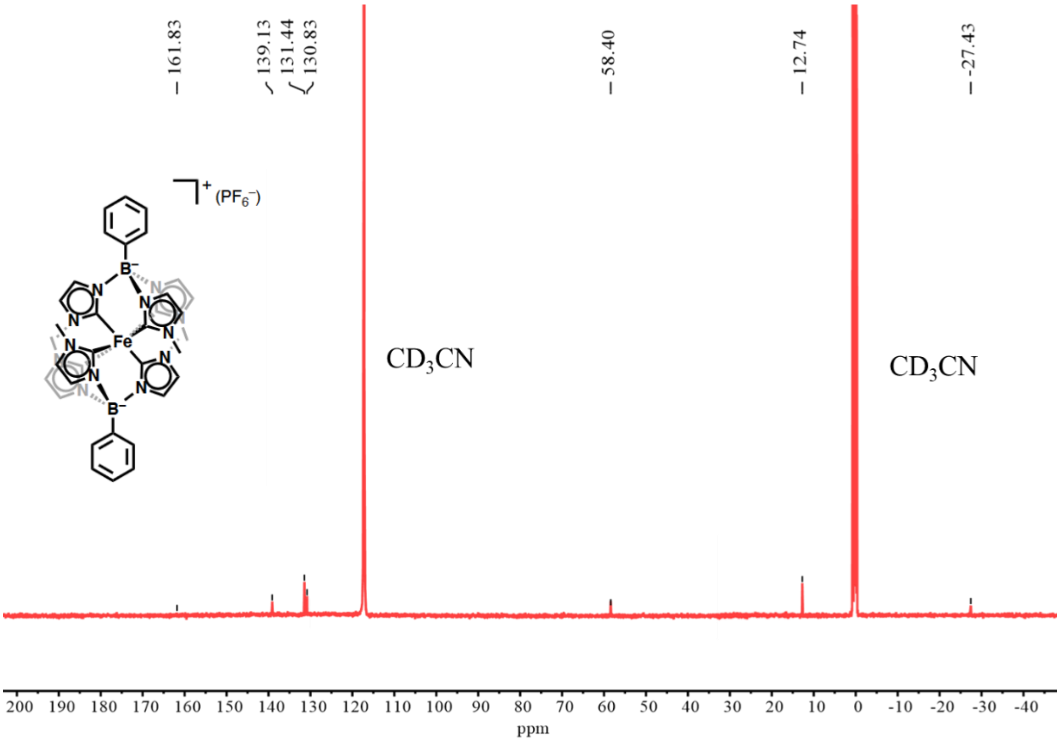

Figure S60. ^{13}C NMR spectrum of [FeIII(phtmeimb)$_2$]PF$_6$ measured in acetonitrile-d_3 at 25 °C.

13. References

(1) Wang, J.-W.; Huang, H.-H.; Wang, P.; Yang, G.; Kupfer, S.; Huang, Y.; Li, Z.; Ke, Z.; Ouyang, G. Co-facial π–π Interaction Expedites Sensitizer-to-Catalyst Electron Transfer for High-Performance CO2 Photoreduction. *JACS Au* **2022**, *2,* 1359-1374.

(2) Thordarson, P. Determining association constants from titration experiments in supramolecular chemistry. *Chem. Soc. Rev.* **2011**, *40,* 1305-1323.

(3) Suzuki, K.; Kobayashi, A.; Kaneko, S.; Takehira, K.; Yoshihara, T.; Ishida, H.; Shiina, Y.; Oishi, S.; Tobita, S. Reevaluation of absolute luminescence quantum yields of standard solutions using a spectrometer with an integrating sphere and a back-thinned CCD detector. *Phys. Chem. Chem. Phys.* **2009**, *11,* 9850-9860.

(4) Wang, C.; Reichenauer, F.; Kitzmann, W. R.; Kerzig, C.; Heinze, K.; Resch-Genger, U. Efficient Triplet-Triplet Annihilation Upconversion Sensitized by a Chromium(**III**) Complex via an Underexplored Energy Transfer Mechanism. *Angew. Chem. Int. Ed.* **2022**, *61,* e202202238.

(5) Thoi, V. S.; Kornienko, N.; Margarit, C. G.; Yang, P.; Chang, C. J. Visible-light photoredox catalysis: selective reduction of carbon dioxide to carbon monoxide by a nickel N-heterocyclic carbene-isoquinoline complex. *J. Am. Chem. Soc.* **2013**, *135,* 14413-14424.

(6) *Gaussian 16 Rev. B.01*; Wallingford, CT, 2016.

(7) Mulliken, R. S. Criteria for the Construction of Good Self-Consistent-Field Molecular Orbital Wave Functions, and the Significance of LCAO-MO Population Analysis. *J. Chem. Phys.* **1962**, *36,* 3428-3439.

(8) Bonaccorsi, R.; Scrocco, E.; Tomasi, J. Molecular SCF Calculations for the Ground State of Some Three-Membered Ring Molecules: (CH2)3, (CH2)2NH, (CH2)2NH2+, (CH2)2O, (CH2)2S, (CH)2CH2, and N2CH2. *J. Chem. Phys.* **1970**, *52,* 5270-5284.

(9) Forshaw, A. P.; Bontchev, R. P.; Smith, J. M. Oxidation of the Tris(carbene)borate Complex PhB(MeIm)3MnI(CO)3 to MnIV[PhB(MeIm)3]2(OTf)2. *Inorg. Chem.* **2007**, *46,* 3792-3794.

(10) Kjær, K. S.; Kaul, N.; Prakash, O.; Chábera, P.; Rosemann, N. W.; Honarfar, A.; Gordivska, O.; Fredin, L. A.; Bergquist, K.-E.; Häggström, L.; et al. Luminescence and reactivity of a charge-transfer excited iron complex with nanosecond lifetime. *Science* **2019**, *363,* 249-253.

(11) Nandi, A.; Manna, B.; Ghosh, R. Interplay of exciton-excimer dynamics in 9,10-diphenylanthracene nanoaggregates and thin films revealed by time-resolved spectroscopic studies. *Phys. Chem. Chem. Phys.* **2019**, *21,* 11193-11202.

(12) Manna, B.; Nandi, A.; Nath, S.; Agarwal, N.; Ghosh, R. Comparative studies of photophysics and exciton dynamics of different diphenylanthracene (DPA) nanoaggregates. *J. Photochem. Photobiol. A* **2020**, *400,* 112700.

(13) Montalti, M.; Credi, A.; Prodi, L.; Gandolfi, M. T. *Handbook of Photochemistry*; CRC Press, 2006.

(14) Farrán, A.; Deshayes, K. D. Free Energy Dependence of Intermolecular Triplet Energy Transfer: Observation of the Inverted Region. *J. Phys. Chem.* **1996**, *100,* 3305–3307.

(15) S. De Kreijger; F. Glaser; Troian-Gautier, L. From Photons to Reactions: Key Concepts

in Photoredox Catalysis. *Chem Catal.* **2024**, 101110.
(16) Jin, Y.; Zhang, Q.; Wang, L.; Wang, X.; Meng, C.; Duan, C. Convenient C(sp^3)–H bond functionalisation of light alkanes and other compounds by iron photocatalysis. *Green Chem.* **2021**, *23,* 6984– 6989.
(17) Nielsen, M. K.; Shields, B. J.; Liu, J.; Williams, M. J.; Zacuto, M. J.; Doyle, A. G. Mild, Redox-Neutral Formylation of Aryl Chlorides through the Photocatalytic Generation of Chlorine Radicals. *Angew. Chem. Int. Ed.* **2017**, *56,* 7191-7194.
(18) Tolba, M.; Elmansi, H. Studying the quenching resulted from the formation of an association complex between olsalazine or sulfasalazine with acriflavine. *R. Soc. Open. Sci.* **2021**, *8,* 210110.
(19) Gillespie, J. E.; Fanourakis, A.; Phipps, R. J. Strategies That Utilize Ion Pairing Interactions to Exert Selectivity Control in the Functionalization of C-H Bonds. *J. Am. Chem. Soc.* **2022**, *144,* 18195-18211.
(20) Glaser, F.; Schmitz, M.; Kerzig, C. Coulomb interactions for mediator-enhanced sensitized triplet-triplet annihilation upconversion in solution. *Nanoscale* **2023**, *16,* 123-137.
(21) Alonso, R.; Bach, T. A chiral thioxanthone as an organocatalyst for enantioselective [2+2] photocycloaddition reactions induced by visible light. *Angew. Chem. Int. Ed.* **2014**, *53,* 4368-4371.
(22) Wu, S.; Žurauskas, J.; Domański, M.; Hitzfeld, P. S.; Butera, V.; Scott, D. J.; Rehbein, J.; Kumar, A.; Thyrhaug, E.; Hauer, J.; et al. Hole-mediated photoredox catalysis: tris(p-substituted)biarylaminium radical cations as tunable, precomplexing and potent photooxidants. *Org. Chem. Front.* **2021**, *8,* 1132-1142.
(23) Wang, J. W.; Huang, H. H.; Wang, P.; Yang, G.; Kupfer, S.; Huang, Y.; Li, Z.; Ke, Z.; Ouyang, G. Co-facial π–π Interaction Expedites Sensitizer-to-Catalyst Electron Transfer for High-Performance CO_2 Photoreduction. *JACS Au* **2022**, *2,* 1359-1374.
(24) Aydogan, A.; Bangle, R. E.; Cadranel, A.; Turlington, M. D.; Conroy, D. T.; Cauët, E.; Singleton, M. L.; Meyer, G. J.; Sampaio, R. N.; Elias, B.; et al. Accessing Photoredox Transformations with an Iron(III) Photosensitizer and Green Light. *J. Am. Chem. Soc.* **2021**, *143,* 15661-15673.
(25) Wigner, E. Nachr. Akad. Wiss. Goettingen. *Math Physik, Kl, IIa* **1927**, 375.
(26) Lee, A. R.; Enos, C. S.; Brenton, A. G. Collisional excitation of CO: a study of the wigner spin rule. *Int. J. Mass Spectrom. Ion Processes* **1991**, *104,* 49-62.
(27) Guo, D.; Knight, T. E.; McCusker, J. K. Angular Momentum Conservation in Dipolar Energy Transfer. *Science* **2011**, *334,* 1684-1687.
(28) S. Trippmacher; S. Demeshko; A. Prescimone; F. Meyer; O. S. Wenger; Wang, C. Ferromagnetically coupled chromium(III) dimer shows luminescence and sensitizes photon upconversion. *Chem. Eur. J.* **2024**, *30,* e202400856.
(29) Talukder, M. M.; Cue, J. M. O.; Miller, J. T.; Gamage, P. L.; Aslam, A.; McCandless, G. T.; Biewer, M. C.; Stefan, M. C. Ligand Steric Effects of α-Diimine Nickel(II) and Palladium(II) Complexes in the Suzuki–Miyaura Cross-Coupling Reaction. *ACS Omega* **2020**, *5,* 24018-24032.
(30) Yuan, Y.-X.; Jia, J.-H.; Song, Y.-P.; Ye, F.-Y.; Zheng, Y.-S.; Zang, S.-Q. Fluorescent TPE Macrocycle Relayed Light-Harvesting System for Bright Customized-Color Circularly

Polarized Luminescence. *J. Am. Chem. Soc.* **2022**, *144,* 5389-5399.
(31) Chwastek, M.; Cmoch, P.; Szumna, A. Dodecameric Anion-Sealed Capsules based on Pyrogallol[5]arenes and Resorcin[5]arenes. *Angew. Chem. Int. Ed.* **2021**, *60,* 4540-4544.
(32) Wu, H.-L.; zhang, M.-Y.; Zhou, T.; Zhang, L.-P.; Qi, Q.-Y.; Yang, G.-Y.; Yang, B.; Li, Z.-T. Six-Cyclic Crown Ether-Type Pillar[5]arene: Enhanced Binding Ability to Bispyridinium Derivatives. *Chem. Asian J.* **2024**, *19,* e202400554.
(33) Zhou, Y.; Tang, H.; Wu, H.; Jiang, X.; Wang, L.; Cao, D. Supramolecular cyclization induced emission enhancement in a pillar[5]arene probe for discrimination of spermine. *Chin. Chem. Lett.* **2024**, *35,* 108626.
(34) Cheung, P. L.; Kapper, S. C.; Zeng, T.; Thompson, M. E.; Kubiak, C. P. Improving Photocatalysis for the Reduction of CO2 through Non-covalent Supramolecular Assembly. *J. Am. Chem. Soc.* **2019**, *141,* 14961-14965.
(35) Rosemann, N. W.; Lindh, L.; Bolano Losada, I.; Kaufhold, S.; Prakash, O.; Ilic, A.; Schwarz, J.; Warnmark, K.; Chabera, P.; Yartsev, A.; et al. Competing dynamics of intramolecular deactivation and bimolecular charge transfer processes in luminescent Fe(iii) N-heterocyclic carbene complexes. *Chem. Sci.* **2023**, *14,* 3569-3579.
(36) Genovese, D.; Cingolani, M.; Rampazzo, E.; Prodi, L.; Zaccheroni, N. Static quenching upon adduct formation: a treatment without shortcuts and approximations. *Chem. Soc. Rev.* **2021**, *50,* 8414-8427.
(37) Hou, L.; Olesund, A.; Thurakkal, S.; Zhang, X.; Albinsson, B. Efficient Visible-to-UV Photon Upconversion Systems Based on CdS Nanocrystals Modified with Triplet Energy Mediators. *Adv. Funct. Mater.* **2021**, *31,* 2106198.
(38) Li, H.; Wang, C.; Glaser, F.; Sinha, N.; Wenger, O. S. Metal-Organic Bichromophore Lowers the Upconversion Excitation Power Threshold and Promotes UV Photoreactions. *J. Am. Chem. Soc.* **2023**, *145,* 11402–11414.
(39) Edhborg, F.; Olesund, A.; Albinsson, B. Best practice in determining key photophysical parameters in triplet-triplet annihilation photon upconversion. *Photochem. Photobiol. Sci.* **2022**, *21,* 1143-1158.
(40) Wang, C.; Wegeberg, C.; Wenger, O. S. First-Row d^6 Metal Complex Enables Photon Upconversion and Initiates Blue Light-Dependent Polymerization with Red Light. *Angew. Chem. Int. Ed.* **2023**, *62,* e202311470.
(41) Olesund, A.; Johnsson, J.; Edhborg, F.; Ghasemi, S.; Moth-Poulsen, K.; Albinsson, B. Approaching the Spin-Statistical Limit in Visible-to-Ultraviolet Photon Upconversion. *J. Am. Chem. Soc.* **2022**, *144,* 3706-3716.
(42) Gray, V.; Dzebo, D.; Lundin, A.; Alborzpour, J.; Abrahamsson, M.; Albinsson, B.; Moth-Poulsen, K. Photophysical characterization of the 9,10-disubstituted anthracene chromophore and its applications in triplet–triplet annihilation photon upconversion. *J. Mater. Chem. C* **2015**, *3,* 11111-11121.
(43) Durandin, N. A.; Isokuortti, J.; Efimov, A.; Vuorimaa-Laukkanen, E.; Tkachenko, N. V.; Laaksonen, T. Critical Sensitizer Quality Attributes for Efficient Triplet–Triplet Annihilation Upconversion with Low Power Density Thresholds. *J. Phys. Chem. C* **2019**, *123,* 22865-22872.
(44) Durandin, N. A.; Isokuortti, J.; Efimov, A.; Vuorimaa-Laukkanen, E.; Tkachenko, N. V.;

Laaksonen, T. Efficient photon upconversion at remarkably low annihilator concentrations in a liquid polymer matrix: when less is more. *Chem. Commun.* **2018**, *54,* 14029-14032.
(45) McCusker, C. E.; Castellano, F. N. Efficient Visible to Near-UV Photochemical Upconversion Sensitized by a Long Lifetime Cu(I) MLCT Complex. *Inorg. Chem.* **2015**, *54,* 6035-6042.
(46) Wei, Y.; Li, Y.; Li, Z.; Xu, X.; Cao, X.; Zhou, X.; Yang, C. Efficient Triplet-Triplet Annihilation Upconversion in Solution and Hydrogel Enabled by an S-T Absorption Os(II) Complex Dyad with an Elongated Triplet Lifetime. *Inorg. Chem.* **2021**, *60,* 19001-19008.
(47) Klán, P.; Wirz, J. *Photochemistry of Organic Compounds: From Concepts to Practice*; Wiley, 2009.
(48) Szymaszek, P.; Tomal, W.; Świergosz, T.; Kamińska-Borek, I.; Popielarz, R.; Ortyl, J. Review of quantitative and qualitative methods for monitoring photopolymerization reactions. *Polym. Chem.* **2023**, *14,* 1690-1717.
(49) Lang, M.; Hirner, S.; Wiesbrock, F.; Fuchs, P. A Review on Modeling Cure Kinetics and Mechanisms of Photopolymerization. *Polymers* **2022**, *14,* 2074.
(50) Chiulan, I.; Heggset, E. B.; Voicu, Ş. I.; Chinga-Carrasco, G. Photopolymerization of Bio-Based Polymers in a Biomedical Engineering Perspective. *Biomacromolecules* **2021**, *22,* 1795-1814.
(51) Ajekwene, K. Properties and Applications of Acrylates. In *Acrylate Polymers for Advanced Applications*, Serrano-Aroca, Á., Deb, S. Eds.; IntechOpen, 2020.
(52) Corsaro, C.; Neri, G.; Santoro, A.; Fazio, E. Acrylate and Methacrylate Polymers' Applications: Second Life with Inexpensive and Sustainable Recycling Approaches. *Materials* **2021**, *15,* 282.
(53) Rocheva, V. V.; Koroleva, A. V.; Savelyev, A. G.; Khaydukov, K. V.; Generalova, A. N.; Nechaev, A. V.; Guller, A. E.; Semchishen, V. A.; Chichkov, B. N.; Khaydukov, E. V. High-resolution 3D photopolymerization assisted by upconversion nanoparticles for rapid prototyping applications. *Sci. Rep.* **2018**, *8,* 3663.
(54) Wang, K.; Peña, J.; Xing, J. Upconversion Nanoparticle-Assisted Photopolymerization. *Photochem. Photobiol.* **2020**, *96,* 741-749.
(55) Wu, C.; Corrigan, N.; Lim, C. H.; Liu, W.; Miyake, G.; Boyer, C. Rational Design of Photocatalysts for Controlled Polymerization: Effect of Structures on Photocatalytic Activities. *Chem. Rev.* **2022**, *122,* 5476-5518.
(56) Zivic, N.; Bouzrati-Zerelli, M.; Kermagoret, A.; Dumur, F.; Fouassier, J. P.; Gigmes, D.; Lalevée, J. Photocatalysts in Polymerization Reactions. *ChemCatChem* **2016**, *8,* 1617-1631.
(57) Bagheri, A.; Jin, J. Photopolymerization in 3D Printing. *ACS Appl. Polym. Mater.* **2019**, *1,* 593-611.
(58) Sanders, S. N.; Schloemer, T. H.; Gangishetty, M. K.; Anderson, D.; Seitz, M.; Gallegos, A. O.; Stokes, R. C.; Congreve, D. N. Triplet fusion upconversion nanocapsules for volumetric 3D printing. *Nature* **2022**, *604,* 474-478.
(59) Wei, L.; Yang, C.; Wu, W. Recent advances of stereolithographic 3D printing enabled by photon upconversion technology. *Curr. Opin. Green Sust. Chem.* **2023**, *43,* 100851.
(60) Serevičius, T.; Komskis, R.; Adomėnas, P.; Adomėnienė, O.; Kreiza, G.; Jankauskas, V.; Kazlauskas, K.; Miasojedovas, A. n.; Jankus, V.; Monkman, A.; et al. Triplet–Triplet

Annihilation in 9,10-Diphenylanthracene Derivatives: The Role of Intersystem Crossing and Exciton Diffusion. *J. Phys. Chem. C* **2017**, *121,* 8515-8524.
(61) Fan, C.; Wei, L.; Niu, T.; Rao, M.; Cheng, G.; Chruma, J. J.; Wu, W.; Yang, C. Efficient Triplet-Triplet Annihilation Upconversion with an Anti-Stokes Shift of 1.08 eV Achieved by Chemically Tuning Sensitizers. *J. Am. Chem. Soc.* **2019**, *141,* 15070-15077.
(62) Hu, A.; Chen, Y.; Guo, J. J.; Yu, N.; An, Q.; Zuo, Z. Cerium-Catalyzed Formal Cycloaddition of Cycloalkanols with Alkenes through Dual Photoexcitation. *J. Am. Chem. Soc.* **2018**, *140,* 13580-13585.
(63) Neumeier, M.; Chakraborty, U.; Schaarschmidt, D.; de la Pena O'Shea, V.; Perez-Ruiz, R.; Jacobi von Wangelin, A. Combined Photoredox and Iron Catalysis for the Cyclotrimerization of Alkynes. *Angew. Chem. Int. Ed.* **2020**, *59,* 13473-13478.
(64) Allushi, A.; Jockusch, S.; Yilmaz, G.; Yagci, Y. Photoinitiated Metal-Free Controlled/Living Radical Polymerization Using Polynuclear Aromatic Hydrocarbons. *Macromolecules* **2016**, *49,* 7785-7792.
(65) Parkatzidis, K.; Rolland, M.; Truong, N. P.; Anastasaki, A. Tailoring polymer dispersity by mixing ATRP initiators. *Polym. Chem.* **2021**, *12,* 5583-5588.
(66) Hammecke, H.; Fritzler, D.; Vashistha, N.; Jin, P.; Dietzek-Ivanšić, B.; Wang, C. 100 µs Luminescence Lifetime Boosts the Excited State Reactivity of a Ruthenium(II)-Anthracene Complex in Photon Upconversion and Photocatalytic Polymerizations with Red Light. *Chem. Eur. J.* **2024**. e202402679.
(67) Bürgin, T. H.; Glaser, F.; Wenger, O. S. Shedding Light on the Oxidizing Properties of Spin-Flip Excited States in a CrIII Polypyridine Complex and Their Use in Photoredox Catalysis. *J. Am. Chem. Soc.* **2022**, *144,* 14181-14194.
(68) Bock, C. R.; Connor, J. A.; Gutierrez, A. R.; Meyer, T. J.; Whitten, D. G.; Sullivan, B. P.; Nagle, J. K. Estimation of excited-state redox potentials by electron-transfer quenching. Application of electron-transfer theory to excited-state redox processes. *J. Am. Chem. Soc.* **1979**, *101,* 4815-4824.
(69) Rehm, D.; Weller, A. Kinetik und Mechanismus der Elektronübertragung bei der Fluoreszenzlöschung in Acetonitril. *Berichte der Bunsengesellschaft für physikalische Chemie* **1969**, *73,* 834-839.
(70) Rehm, D.; Weller, A. Kinetics of Fluorescence Quenching by Electron and H-Atom Transfer. *Isr. J. Chem.* **1970**, *8,* 259-271.
(71) Caron, A.; Noirbent, G.; Gigmes, D.; Dumur, F.; Lalevée, J. Near-Infrared PhotoInitiating Systems: Photothermal Effect vs. Triplet-Triplet Annihilation -based UpConversion Polymerization. *Macromol. Rapid. Commun.* **2021**, *42,* 2100047.
(72) Limberg, D. K.; Kang, J. H.; Hayward, R. C. Triplet-Triplet Annihilation Photopolymerization for High-Resolution 3D Printing. *J. Am. Chem. Soc.* **2022**, *144,* 5226-5232.
(73) Wong, J.; Wei, S.; Meir, R.; Sadaba, N.; Ballinger, N. A.; Harmon, E. K.; Gao, X.; Altin-Yavuzarslan, G.; Pozzo, L. D.; Campos, L. M.; et al. Triplet Fusion Upconversion for Photocuring 3D-Printed Particle-Reinforced Composite Networks. *Adv. Mater.* **2023**, *35,* e2207673.
(74) Ding, C.; Wang, J.; Zhang, W.; Pan, X.; Zhang, Z.; Zhang, W.; Zhu, J.; Zhu, X. Platform

of near-infrared light-induced reversible deactivation radical polymerization: upconversion nanoparticles as internal light sources. *Polym. Chem.* **2016**, *7,* 7370-7374.
(75) Wang, K.; Pena, J.; Xing, J. Upconversion Nanoparticle-Assisted Photopolymerization. *Photochem. Photobiol.* **2020**, *96,* 741-749.
(76) Awwad, N.; Bui, A. T.; Danilov, E. O.; Castellano, F. N. Visible-Light-Initiated Free-Radical Polymerization by Homomolecular Triplet-Triplet Annihilation. *Chem* **2020**, *6,* 3071-3085.
(77) Ravetz, B. D.; Pun, A. B.; Churchill, E. M.; Congreve, D. N.; Rovis, T.; Campos, L. M. Photoredox catalysis using infrared light via triplet fusion upconversion. *Nature* **2019**, *565,* 343-346.
(78) Liang, W.; Nie, C.; Du, J.; Han, Y.; Zhao, G.; Yang, F.; Liang, G.; Wu, K. Near-infrared photon upconversion and solar synthesis using lead-free nanocrystals. *Nat. Photon.* **2023**, *17,* 346-353.
(79) Miyake, G. M.; Theriot, J. C. Perylene as an Organic Photocatalyst for the Radical Polymerization of Functionalized Vinyl Monomers through Oxidative Quenching with Alkyl Bromides and Visible Light. *Macromolecules* **2014**, *47,* 8255-8261.
(80) Jordan C. Theriot; Chern-Hooi Lim; Haishen Yang; Matthew D. Ryan; Charles B. Musgrave, G. M. M. Organocatalyzed atom transfer radical polymerization driven by visible light. *Science* **2016**, *352,* 1082-1086.
(81) Discekici, E. H.; Anastasaki, A.; Read de Alaniz, J.; Hawker, C. J. Evolution and Future Directions of Metal-Free Atom Transfer Radical Polymerization. *Macromolecules* **2018**, *51,* 7421-7434.
(82) Corbin, D. A.; Miyake, G. M. Photoinduced Organocatalyzed Atom Transfer Radical Polymerization (O-ATRP): Precision Polymer Synthesis Using Organic Photoredox Catalysis. *Chem. Rev.* **2022**, *122,* 1830-1874.
(83) Bortolato, T.; Simionato, G.; Vayer, M.; Rosso, C.; Paoloni, L.; Benetti, E. M.; Sartorel, A.; Leboeuf, D.; Dell'Amico, L. The Rational Design of Reducing Organophotoredox Catalysts Unlocks Proton-Coupled Electron-Transfer and Atom Transfer Radical Polymerization Mechanisms. *J. Am. Chem. Soc.* **2023**, *145,* 1835-1846.
(84) Glaser, F.; Kerzig, C.; Wenger, O. S. Sensitization-initiated electron transfer via upconversion: mechanism and photocatalytic applications. *Chem. Sci.* **2021**, *12,* 9922-9933.
(85) Glaser, F.; Wenger, O. S. Sensitizer-controlled photochemical reactivity via upconversion of red light. *Chem. Sci.* **2023**, *14,* 149-161.
(86) Majek, M.; Faltermeier, U.; Dick, B.; Perez-Ruiz, R.; Jacobi von Wangelin, A. Application of Visible-to-UV Photon Upconversion to Photoredox Catalysis: The Activation of Aryl Bromides. *Chem. Eur. J.* **2015**, *21,* 15496-15501.
(87) Richards, B. S.; Hudry, D.; Busko, D.; Turshatov, A.; Howard, I. A. Photon Upconversion for Photovoltaics and Photocatalysis: A Critical Review. *Chem. Rev.* **2021**, *121,* 9165-9195.
(88) Amos, S. G. E.; Garreau, M.; Buzzetti, L.; Waser, J. Photocatalysis with organic dyes: facile access to reactive intermediates for synthesis. *Beilstein J. Org. Chem.* **2020**, *16,* 1163-1187.
(89) Zoroddu, M. A.; Aaseth, J.; Crisponi, G.; Medici, S.; Peana, M.; Nurchi, V. M. The essential metals for humans: a brief overview. *J. Inorg. Biochem.* **2019**, *195,* 120-129.
(90) Nazanin Abbaspour, R. H.; Kelishadi, R. Review on iron and its importance for human

health. *J. Res. Med. Sci.* **2014**, *19,* 164-174
(91) Abhijnakrishna, R.; Magesh, K.; Ayushi, A.; Velmathi, S. Advances in the Biological Studies of Metal-Terpyridine Complexes: An Overview From 2012 to 2022. *Coord. Chem. Rev.* **2023**, *496*.
(92) Kirkland, D.; Fowler, P. A review of the genotoxicity of trimethylolpropane triacrylate (TMPTA). *Mutat. Res. Gen. Tox. En.* **2018**, *828,* 36-45.
(93) Kotturi, H.; Abuabed, A.; Zafar, H.; Sawyer, E.; Pallipparambil, B.; Jamadagni, H.; Khandaker, M. Evaluation of Polyethylene Glycol Diacrylate-Polycaprolactone Scaffolds for Tissue Engineering Applications. *J. Funct. Biomater.* **2017**, *8,* 39.
(94) Schloemer, T.; Narayanan, P.; Zhou, Q.; Belliveau, E.; Seitz, M.; Congreve, D. N. Nanoengineering Triplet-Triplet Annihilation Upconversion: From Materials to Real-World Applications. *ACS Nano* **2023**, *17,* 3259-3288.
(95) Hu, X.; Szczepaniak, G.; Lewandowska-Andralojc, A.; Jeong, J.; Li, B.; Murata, H.; Yin, R.; Jazani, A. M.; Das, S. R.; Matyjaszewski, K. Red-Light-Driven Atom Transfer Radical Polymerization for High-Throughput Polymer Synthesis in Open Air. *J. Am. Chem. Soc.* **2023**, *145,* 24315-24327.
